# Potential mechanisms of Guizhi decoction against hypertension based on network pharmacology and Dahl salt-sensitive rat model

**DOI:** 10.1186/s13020-021-00446-x

**Published:** 2021-04-27

**Authors:** Jiye Chen, Yongjian Zhang, Yongcheng Wang, Ping Jiang, Guofeng Zhou, Zhaoyu Li, Jinlong Yang, Xiao Li

**Affiliations:** 1 First Clinical Medical College, Shandong University of Traditional Chinese Medicine, Jinan, 250014 China; 2 Department of Cardiovascular, Affiliated Hospital of Shandong University of Traditional Chinese Medicine, Jinan, 250011 China

**Keywords:** Guizhi decoction, Network pharmacology, Hypertension, Molecular docking, Molecular mechanism

## Abstract

**Background:**

Guizhi decoction (GZD), a classical Chinese herbal formula, has been widely used to treat hypertension, but its underlying mechanisms remain elusive. The present study aimed to explore the potential mechanisms and therapeutic effects of GZD on hypertension by integrating network pharmacology and experimental validation.

**Methods:**

The active ingredients and corresponding targets were collected from the Traditional Chinese Medicine Systems Pharmacology database and Analysis Platform (TCMSP). The targets related to hypertension were identified from the CTD, GeneCards, OMIM and Drugbank databases. Multiple networks were constructed to identify the key compounds, hub targets, and main biological processes and pathways of GZD against hypertension. The Surflex-Dock software was used to validate the binding affinity between key targets and their corresponding active compounds. The Dahl salt-sensitive rat model was used to evaluate the therapeutic effects of GZD against hypertension.

**Results:**

A total of 112 active ingredients, 222 targets of GZD and 341 hypertension-related targets were obtained. Furthermore, 56 overlapping targets were identified, five of which were determined as the hub targets for experimental verification, including interleukin 6 (IL-6), C–C motif chemokine 2 (CCL2), IL-1*β*, matrix metalloproteinase 2 (MMP-2), and MMP-9. Pathway enrichment analysis results indicated that 56 overlapping targets were mainly enriched in several inflammation pathways such as the tumor necrosis factor (TNF) signaling pathway, Toll-like receptor (TLR) signaling pathway and nuclear factor kappa-B (NF-κB) signaling pathway. Molecular docking confirmed that most active compounds of GZD could bind tightly to the key targets. Experimental studies revealed that the administration of GZD improved blood pressure, reduced the area of cardiac fibrosis, and inhibited the expression of IL-6, CCL2, IL-1*β*, MMP-2 and MMP-9 in rats.

**Conclusion:**

The potential mechanisms and therapeutic effects of GZD on hypertension may be attributed to the regulation of cardiac inflammation and fibrosis.

**Supplementary Information:**

The online version contains supplementary material available at 10.1186/s13020-021-00446-x.

## Background

Hypertension is one of the most common cardiovascular diseases worldwide [1]. Although substantial progress in the diagnosis and therapy of hypertension has been made in recent years, it remains one of the most severe public health issues in the world. It is predicted that more than 1.5 billion people worldwide will suffer from hypertension by 2025, and this situation is expected to worsen over the next decade with the increasing global and ageing populations [2, 3]. Furthermore, long-lasting hypertension can lead to myocardial infarction, stroke, chronic kidney dysfunction, heart failure, and other complications, which are the major causes of disability and premature death in humans [4]. Generally, the pathological mechanisms of hypertension include the overactivation of the renin–angiotensin–aldosterone system (RAAS) and sympathetic nervous system (SNS), and sodium and water retention [2, 4]. Hence, the first-line treatments for hypertension primarily include diuretics, *β-*blockers, calcium channel blockers, angiotensin-converting enzyme inhibitors, and angiotensin II receptor blockers [5]. However, some antihypertensive medications are reportedly associated with numerous side effects including peripheral edema, anemia, persistent cough, and decreased sexual function [2, 4, 5]. Therefore, it is necessary to develop more feasible and safer therapeutic strategies for the management of hypertension.

Cumulative valuable research demonstrated that Chinese medicinal formulas, such as Xiao Yao San, Banxia Baizhu Tianma decoction, and Niuhuang Jiangya preparation, have satisfactory efficacy and minimal side effects in the treatment of hypertension [6–8]. Therefore, several scholars are paying attention to the scientific research value of traditional Chinese medicine (TCM) in the prevention and management of hypertension [6]. According to the TCM therapeutic theory of “regulating Ying-Wei in case of heart damage”, we identified an association between the dysfunction of Ying-Wei and hypertension-related pathological characteristics. TCM pathophysiology and experimental evidence indicate that Spleen and Kidney Yang Deficiency syndrome is the main TCM syndrome related to salt-sensitive hypertension and the main pathological feature of hypertensive Ying-Wei disharmony [9–11]. Guizhi decoction (GZD) is a classical TCM prescription for the treatment of Ying-Wei disharmony and Yin and Yang imbalance [10, 11]. GZD is composed of five Chinese medicinal herbs: *Cinnamomi ramulus* (Guizhi, GZ), *Paeoniae radix alba* (Baishao, BS), *licorice* (Gancao, GC), *Zingiber officinale roscoe* (Shengjiang, SJ), and *Jujubae fructus* (Dazao, DZ). Previously, we found that GZD could effectively reduce blood pressure, prevent myocardial fibrosis, restore balance in the autonomic nervous system, and inhibit the expression of pro-inflammatory cytokines in the Dahl salt-sensitive rats [11]. Additionally, we found that GZD significantly improved heart rate variability and vagus nerve activity, which are associated with the progression of hypertension [10]. A meta-analysis confirmed the beneficial effect and safety of GZD in the treatment of diabetic cardiac autonomic neuropathy [12]. Zheng et al. reported that GZD associated formulas exerted significant effects on the attenuation of moderate-severe painful diabetic peripheral neuropathy [13]. These findings suggest that GZD presents significant advantages on cardiovascular diseases and metabolic diseases.

However, the underlying mechanisms and targets of GZD against hypertension are not fully understood, which have impeded its clinical practice. Further research should be conducted to provide scientific evidences regarding the clinical usage of GZD in hypertension treatment. TCM prescriptions are a complicated system involving multiple biological targets and pathways. This makes it difficult to elucidate their active ingredients and therapeutic mechanisms [14]. Network pharmacology is an innovative approach based on computational systems pharmacology, which can accurately decipher the associations among the drugs, targets, and diseases at a systematic and comprehensive level [15]. Network pharmacology has many same characteristics with TCM, such as the systematic and holistic views. Therefore, the application of network pharmacology can provide a scientific basis for the in-depth study of TCM prescriptions [14]. Increasing evidence supports the reliability of the network pharmacology method, which may be an effective way to study the pharmacological mechanisms of TCM prescriptions [16, 17].

**Fig. 1:**
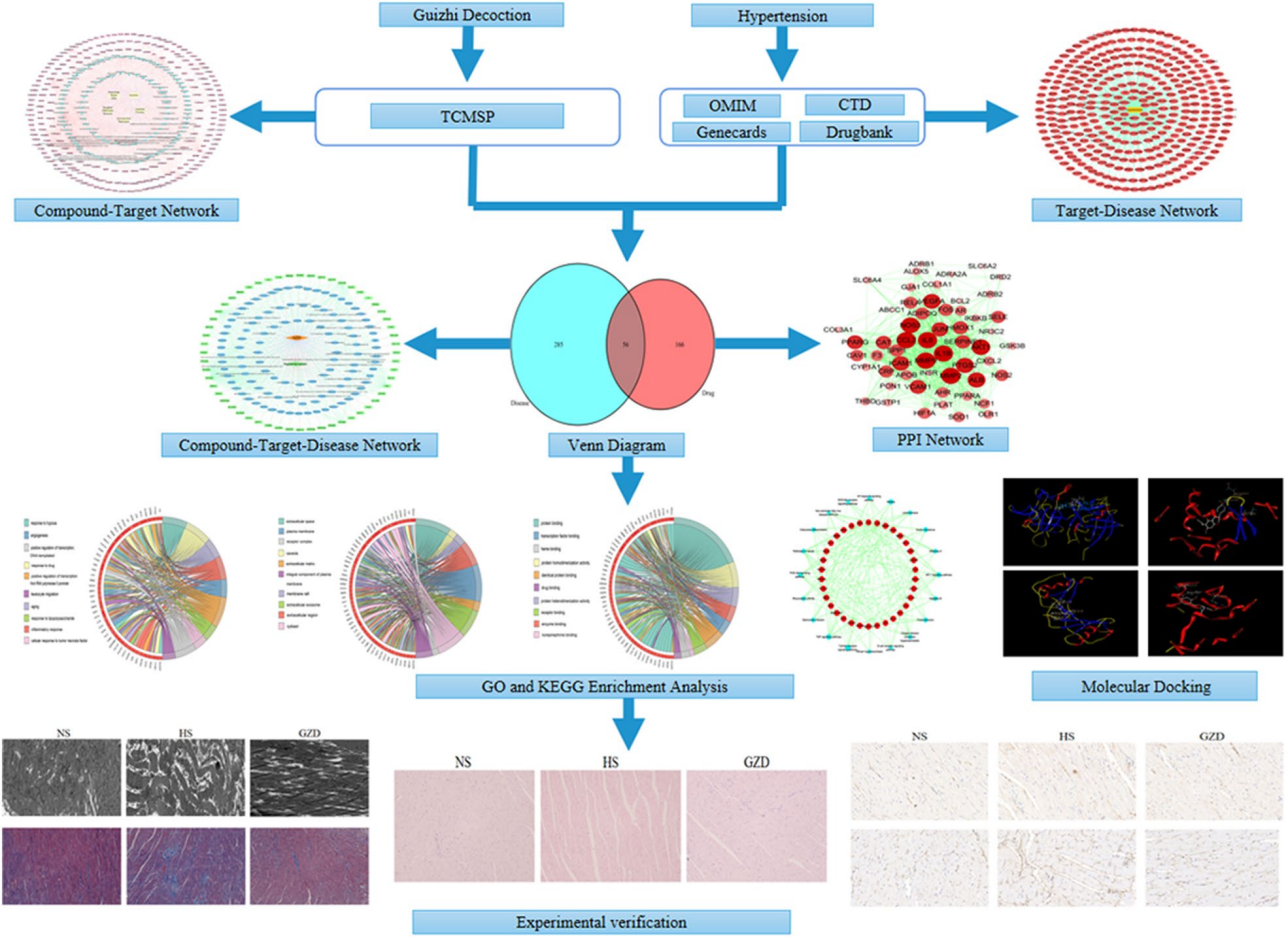
The flowchart of this study based on network pharmacology and experimental validation for deciphering the therapeutic effects and potential mechanisms of GZD against hypertension

In this study, network pharmacology analysis was used to visualize and elucidate the complex relationships among the key compounds, targets, main biological processes, and disease. Moreover, molecular docking experiment was used to validate the binding affinity between key targets and their corresponding active compounds. Finally, the therapeutic effect of GZD on hypertension was validated by an experimental model. The detailed flowchart is illustrated in Fig. 1.

## Materials and methods

### Identification of active compounds and prediction of corresponding targets of Guizhi decoction

The active constituents of the five herbal medicines in GZD were acquired from the Traditional Chinese Medicine Systems Pharmacology database and Analysis Platform (TCMSP) (http://lsp.nwu.edu.cn/tcmsp.php). This database provides comprehensive and accurate compound information, including the herbal ingredients’ chemical structural data, drug half-life, oral bioavailability (OB), intestinal epithelial permeability, and drug-likeness (DL) [18]. According to the recommended drug screening criteria of the TCMSP database, chemical constituents with OB ≥ 30% and DL ≥ 0.18 may present ideal pharmacological activities, and they were selected as the active ingredients for further analysis. Subsequently, we screened the targets of active ingredients in GZD through the TCMSP database. The target names were imported into the UniProt database (http://www.uniprot.org/) with the species selected as “Homo sapiens,” and the gene names of the targets were obtained from the UniProt database.

### Identification of hypertension-related targets

“Hypertension” was used as the keyword to extract the hypertension-related targets from the Online Mendelian Inheritance in Man (OMIM, https://omim.org/), Comparative Toxicogenomics Database (CTD, http://ctdbase.org/), GeneCards (http://www.genecards.org/), and Drugbank (https://www.drugbank.ca/) databases. The disease-related targets obtained were standardized as gene names from the UniProt database with the species selected as “Homo sapiens.” A Venn diagram was drawn using an online website (http://bioinformatics.psb.ugent.be/Webtools/Venn/) to obtain the overlapping targets between the hypertension-related targets and active compound-related targets, which may be the potential targets of GZD in hypertension treatment.

### Construction of protein–protein interaction network and screening of hub targets

The Search Tool for the Retrieval of Interacting Genes (STRING) database (https://string-db.org/) can explore and analyze direct and indirect interactions between proteins [16]. Based on the overlapping targets of GZD and hypertension, we constructed the protein–protein interaction (PPI) network by using the STRING 11.0 database with the species limited to “Homo sapiens” and confidence score > 0.4 [19]. The degree value was used to select the putative targets for molecular docking and experimental verification.

### Gene ontology and pathway enrichment analyses

For better clarification of the potential biological processes and pathways of GZD in the treatment of hypertension in this study, we utilized the Database for Annotation Visualization and Integrated Discovery (DAVID, https://david.ncifcrf.gov/) to conduct gene ontology (GO) function enrichment analysis and the Kyoto Encyclopedia of Genes and Genomes (KEGG) pathway enrichment analysis. The GO terms and pathway terms with p-value < 0.05 were considered as significant enrichment entries.

### Network construction and analysis

In this study, multiple networks were established to visualize and analyze the complicated interconnection of compounds, targets, and disease using Cytoscape 3.7.1 software (https://cytoscape.org/; version 3.7.1). Based on the above results, the compound–target (C–T) network, target–disease (T–D) network, compound–target–disease (C–T–D) network, target–pathway (T–P) network, and compound–target–pathway (C–T–P) network were constructed using Cytoscape software. In these networks, nodes of different colors and shapes represented different active compounds, potential targets, or signal pathways, and the edges represented the connections between the nodes.

### Molecular docking

The Surflex-Dock program in SYBYL 2.1 software (Certara Inc., USA) was used to verify the results of network pharmacology screening by docking the key targets with their active compounds and positive anti-hypertension drugs. The Surflex-Dock program is one of the most efficient ligand-receptor docking techniques and virtual screening programs with favorable features of high precision, high true-positive rate, and fast speed [20]. Common antihypertensive agents, valsartan, candesartan, captopril, enalapril, furosemide, metoprolol, nifedipine, amlodipine, bisoprolol, and hydrochlorothiazide were used as positive drugs for molecular docking. Seventeen key targets with degrees > 30 in the PPI network were selected for docking simulation, and these targets were mapped to 80 active compounds for molecular docking. The three-dimensional structures of the active compounds were downloaded from the PubChem database (https://pubchem.ncbi.nlm.nih.gov/). The structures of the key target proteins were downloaded from the Protein Data Bank database (http://www.pdb.org/) and modified through the Surflex-Dock software [21]. The complex ligand and water molecules in protein receptors were removed, hydrogen atoms were added to the receptor, and amino acids were optimized and patched [22]. After molecular docking with the default parameters, the docking score values were generated for each compound docking with key targets. The docking score could be used to estimate the binding capacity between the targets and their active compounds.

### Experimental animals and protocol

Six-week-old male, specific pathogen-free (SPF) grade Dahl salt-sensitive rats (body weight, 160–180 g) were provided by the Charles River Animal Laboratory (Beijing, China, Certificate No. 2016-0006). The rats were reared in the SPF room at a temperature of 20 ± 2 ℃ and 50 ± 10% humidity on a 12 h light/dark cycle. After acclimatization for 1 week, systolic blood pressure of all rats was measured weekly using a 12-channel tail-cuff blood pressure system (MRBP, IITC Life Science Instruments, USA). The tail-artery pressure was averaged from five successive measurements. All experimental protocols used in this study were performed in accordance with the Institutional Animal Care and Use Committee of Shandong University of Traditional Chinese Medicine (Permit Number: SDVTCM2018071501).

### Preparation of Guizhi decoction and intervention

The herbal medicines were supplied by the Affiliated Hospital of Shandong University of Traditional Chinese Medicine (Jinan, China) and verified by Prof. Feng Li. *Cinnamomi ramulus*, *Paeoniae radix alba*, and *licorice* were mixed in the standard ratio of 3:2:2, and were subjected to reflux extraction twice with 10-times the volume of distilled water for 1 h each. The extracts were then mixed thoroughly and concentrated to a relative density of 1.20–1.25 (70–80 ℃). The solution with 1.5 g/mL of the initial herb was applied in subsequent experiments. After 1 week of acclimatization, the rats were randomly allocated to three groups (n = 8): NS group (normal-salt diet), HS group (high-salt diet), and GZD group. The NS group was fed  with low-salt (0.3% NaCl) diet throughout the experimental period. At 7 weeks of age, the HS group and GZD group were fed with high-salt (8% NaCl) diet progressively developed hypertension. Five weeks after 8% NaCl diet, the mean value of the SBP ≥ 180 mmHg indicated that the model of salt-sensitive hypertension was established successfully according to previous studies [9, 11, 23, 24]. At 12 weeks of age, the NS group and HS group were fed with physiological saline at 2 mL/day, while 2 mL/4.0 g of GZD crude drug/kg/day was administered to the GZD group according to the results of a previous study [11]. All treatments were administered by gastric gavage once daily for 4 weeks.

### Collection of left ventricle tissue

After 4 weeks of the drug treatment, all the rats were anesthetized by intraperitoneal injection of 20 mg/kg pentobarbital sodium. The left ventricle of each rat was carefully isolated and cut into three parts. One part was fixed in 6% paraformaldehyde solution for morphological examination. The second part was fixed in 2.5% glutaraldehyde solution and observed under transmission electron microscope. The last part was placed in liquid nitrogen for western blot analysis and quantitative real-time polymerase chain reaction (qRT- PCR).

### Immunohistochemistry analysis

To further confirm the effects of GZD on cardiac fibrosis, immunohistochemical staining was performed to evaluate the expression levels of collagens I and III in the left ventricle. Collagens I and III are two important molecular mediators of collagen deposition and extracellular matrix (ECM) alterations during the pathological fibrotic process [24]. Furthermore, Collagens I and III also play a relatively important role in the PPI network. Therefore, we selectively measured the expression levels of collagens I and III. Briefly, the tissue sections were placed into 2% hydrogen peroxide for 25 min at room temperature. The slices were subsequently sealed with 5% rabbit serum for 30 min. The sections were incubated with anti-collagen I primary antibody (1:500, ab34710, Abcam) and anti-collagen III primary antibody(1:100, ab6310, Abcam) at 4 ℃ overnight and then washed three times with PBS. Next, the corresponding secondary antibody were incubated with the sections for 50 min. Finally, the sections were immersed in diaminobenzidine and then counterstained with hematoxylin. Five non-overlapping images were randomly selected and observed under the optical microscope. The expression levels of collagen I and III were calculated and quantifed by ImageJ software (NIH, MD, USA).

### Histological examination and transmission electron microscopy

The left ventricles were cut into 4-µm sized sections, and they were subjected to hematoxylin–eosin (H&E) staining, or Masson staining to evaluate inflammatory cell infiltration or cardiac fibrosis, respectively [11]. At least five randomized fields were selected from each tissue sample and evaluated the degree of myocardial injury. Histological evaluation of myocardium in rats was performed using ZEN 1.01.0 Imaging analysis software (Carl Zeiss Microscopy GmbH, German). Additionally, transmission electron microscopy was used to reveal the extent of myocardial damage.

### Real-time PCR

In order to verify the reliability of network pharmacology analysis, qRT-PCR was performed to examine the mRNA expression levels of hub targets in the PPI network. Total RNA was isolated from the left ventricle using TRIzol (Invitrogen, USA), and reverse transcription was performed with the PrimeScript RT reagent kit with gDNA Eraser (Takara, Japan) for 5 min at 85 ℃ according to the manufacturer’s instructions. The Light Cycler 480 SYBR Premix Ex Taq II (Roche, Germany) was used to perform qRT-PCR. The reaction conditions were 94 ℃ for 2 min, 94 ℃ for 30 s, and 60 ℃ for 30 s, and 40 cycles were performed in total. Each RNA sample was performed in triplicate, and the results were normalized with *β*-actin. Relative quantification analysis was performed using the 2^−ΔΔCT^ method.

Primer sequences (synthesized by Accurate Biotechnology Co., Ltd) were designed as follows: IL-6: 5′-ATTGTATGAACAGCGATGATGCAC-3′/5′-CCAGGTAGAAACGGAACTCCAGA-3′; IL-1*β*: 5′-CCCTGAACTCAACTGTGAAATAGCA-3′/5′-CCCAAGTCAAGGGCTTGGAA-3′; CCL2: 5′-CTATGCAGGTCTCTGTCACGCTTC-3′/5′-CAGCCGACTCATTGGGATCA-3′; MMP-2: 5′-ACCTTGACCAGAACACCATCGAG-3′/5′-CAGGGTCCAGGTCAGGTGTGTA-3′; MMP-9: 5′-AGCCGGGAACGTATCTGGA-3′/5′-TGGAAACTCACACGCCAGAAG-3′; and β-actin: 5′-GGAGATTACTGCCCTGGCTCCTA-3′/5′-GACTCATCGTACTCCTGCTTGCTG-3′.

### Western blot analysis

Western blot analysis was performed to assess the protein expression levels of the hub targets in the PPI network. The left ventricles were lysed by adding radioimmunoprecipitation assay lysis (RIPA) buffer (Cat. No. P0013B, Beyotime Biotechnology) and phenylmethylsulfonyl fluoride (PMSF, Cat. No. ST506, Beyotime Biotechnology). The protein concentration was measured using the bicinchoninic acid assay kit (Cat. No. P0010, Beyotime Biotechnology). Equal amounts of protein lysates were separated via appropriate concentration of sodium dodecyl sulfate–polyacrylamide gel electrophoresis (Cat. No. P0012A, Beyotime Biotechnology). The proteins were then transferred to the polyvinylidene difluoride membrane and cultured in 5% non-fat dry milk in Tris buffered saline-Tween 20 (TBST) buffer for 60 min at room temperature. Subsequently, the membranes were incubated with the CCL2 (Cat. No. ab25124, Abcam, 1:2000), IL-6 (Cat. No. ab9324, Abcam, 1:1000), IL-1*β* (Cat. No. ab205924, Abcam, 1:1000), MMP-2 (Cat. No. 10373-2-AP, Proteintech, 1:1000), MMP-9 (Cat. No. 10375-AP, Proteintech, 1:1000), and *β*-actin (Cat. No. ab8226, Abcam, 1:5000) antibodies overnight at 4 ℃. The membranes were rinsed five times in TBST, and then incubated with secondary antibodies for 1 h at room temperature. The protein bands were infiltrated with enhanced chemiluminescence, and then visualized using the FluorChem Q 3.4 system (ProteinSimple, USA).

### Statistical analysis

Data were expressed as the mean ± standard deviation. The differences between groups were analyzed by one-way analysis of variance and independent t-tests using Statistical Package for the Social Sciences 21.0 software (IBM SPSS, Chicago, IL, USA). A p-value < 0.05 indicated statistical significance.

## Results

### Screening of the active compounds and targets prediction

A total of 146 active ingredients of five herbal medicines in GZD were identified based on threshold values of OB ≥ 30% and DL ≥ 0.18, including 7 compounds in GZ, 13 compounds in BS, 92 compounds in GC, 5 compounds in SJ, and 29 compounds in DZ. After deleting the duplicate data, 112 active ingredients were selected for further analysis. The detailed informations of active ingredients are listed in Additional file 1. Moreover, 222 targets of active compounds of GZD were obtained from the TCMSP database and the gene names of these targets were collected via the Uniprot database (Additional file 2).

### Compound–target network construction and analysis

**Fig. 2:**
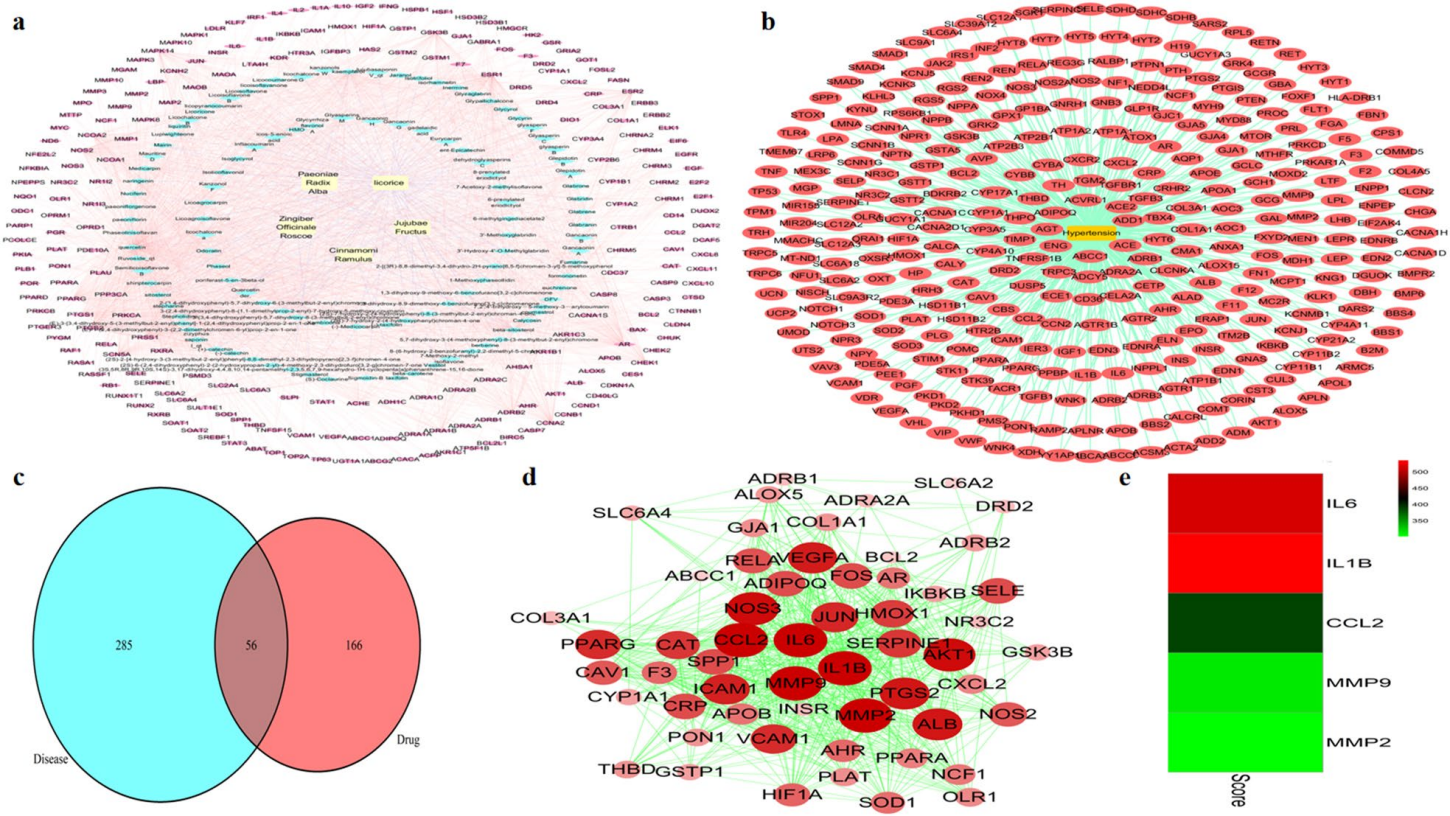
Guizhi decoction potential target-hypertension target network and analysis. **a** Compound–target (C–T) network of Guizhi decoction (GZD): The yellow node represents each herb of GZD, the blue node represents the active compound, and the purple node represents the target of the active compound. **b** Disease-target (D–T) network of hypertension: The red node represents the hypertension-related target. **c** The Venn diagram shows 56 overlapping targets between hypertension-related targets and active compound-related targets. **d** Protein-protein interaction (PPI) network of the 56 overlapping targets is shown here. **e** The heatmap of score between hypertension and hub target

In order to reflect the interactions intuitively between the active compounds of GZD and their potential targets from a systematic and holistic view, the C–T network was constructed by mapping 112 active compounds to their 222 corresponding potential targets. As shown in Fig. 2a, the network consisted of 339 nodes (5 herbal medicines nodes, 112 active compound nodes, and 222 compound-associated target nodes) and 1932 interaction edges. In the network, quercetin (degree 272), *β*-sitosterol (degree 104), and kaempferol (degree 102) presented the maximum interactions with potential targets, indicating that these active compounds with high degree values could play an important role in the potential pharmacological effects of GZD. The C–T network revealed intimate communications between active compounds and related targets, which provided a reference to further investigate the pharmacological mechanisms of GZD.

### Screening of hypertension-related targets and construction of disease–target network

A total of 477 targets associated with hypertension were identified from the CTD, GeneCards, OMIM, and Drugbank databases. We selected the top 200 results related to hypertension with the highest relevance from GeneCards as major targets of hypertension. After duplicate targets were deleted, 341 hypertension-related targets were finally obtained. These targets were then uploaded to Cytoscape 3.7.1 for visualization. The D–T network consisted of 342 nodes (1 hypertension node and 341 hypertension-target nodes) and 477 edges. As shown in Fig. 2b, the top 14 targets with the highest degree values (degree ≥ 100) were recognized as the most crucial targets in the D–T network, namely INS, ALB, AKT1, IL-6, VEGFA, TNF, NOS3, REN, EDN1, AGT, TP53, ACE, FN1, and IGF1. Therefore, these targets may be the potential targets for the treatment of hypertension.

### Construction of PPI network

PPI network was established to better interpret the mechanisms of GZD in hypertension treatment by using STRING software. As shown in Fig. 2c, we obtained 56 overlapping targets after merging hypertension-related targets and active compound-related targets. A PPI network was then established by importing the overlapping targets into the STRING database. As shown in Fig. 2d, the network consisted of 56 nodes and 627 edges with an average node degree of 22.40, network centralization of 0.464, and an average number of neighbors of 22.393. Based on the degree principle of each target, IL-6, CCL2, IL-1*β*, MMP-9, and MMP-2 were determined as the hub targets for experimental verification. In addition, we assessed the associations between hub targets and hypertension via CTD database. As shown in Fig. 2e, we found that these hub targets are closely related to hypertension based on inference score.

### Construction of compound–target–disease network

**Fig. 3:**
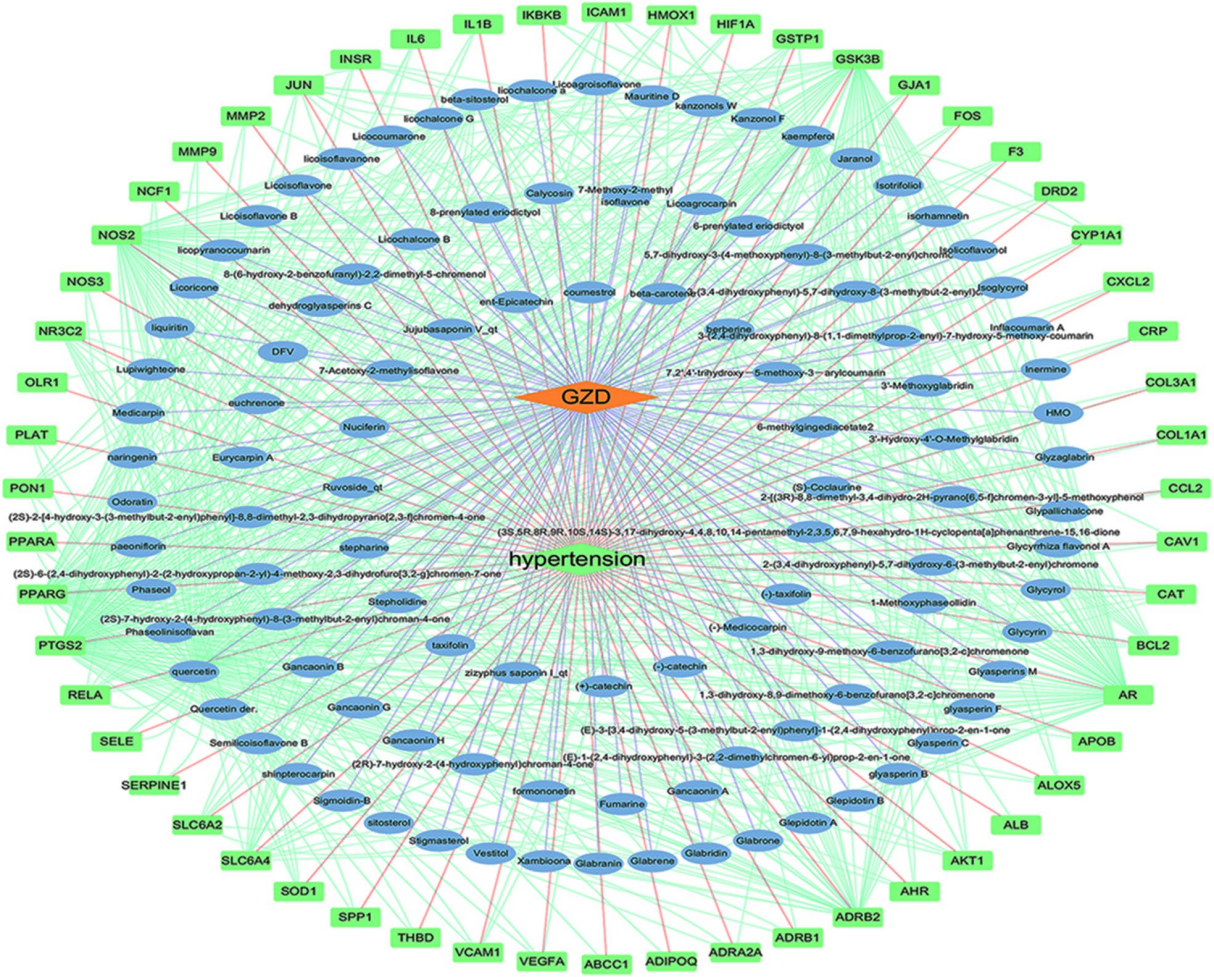
Compound–target–disease (C–T–D) network of Guizhi decoction (GZD) in the treatment of hypertension. The blue circle represents the active compound of GZD in the treatment of hypertension, and the green square pattern represents the overlapping targets

In order to illustrate the complex relationship among the active compounds, overlapping targets, and hypertension, we constructed the C–T–D network. The network comprised of 165 nodes (107 active compounds, 56 potential targets, 1 formula, and 1 disease) and 762 edges. In this network, the key active compounds of GZD against hypertension mainly included quercetin (degree = 84), kaempferol (degree = 40), and *β*-sitosterol (degree = 24), which exhibited degree values higher than that of other compounds. As shown in Fig. 3, the same target could interact with different active compounds, and the same compound could act on multiple targets. These results suggested that GZD exhibits multi-component and multi-target characteristics in the treatment of hypertension.

### Gene ontology enrichment analysis and target-pathway network construction

**Fig. 4:**
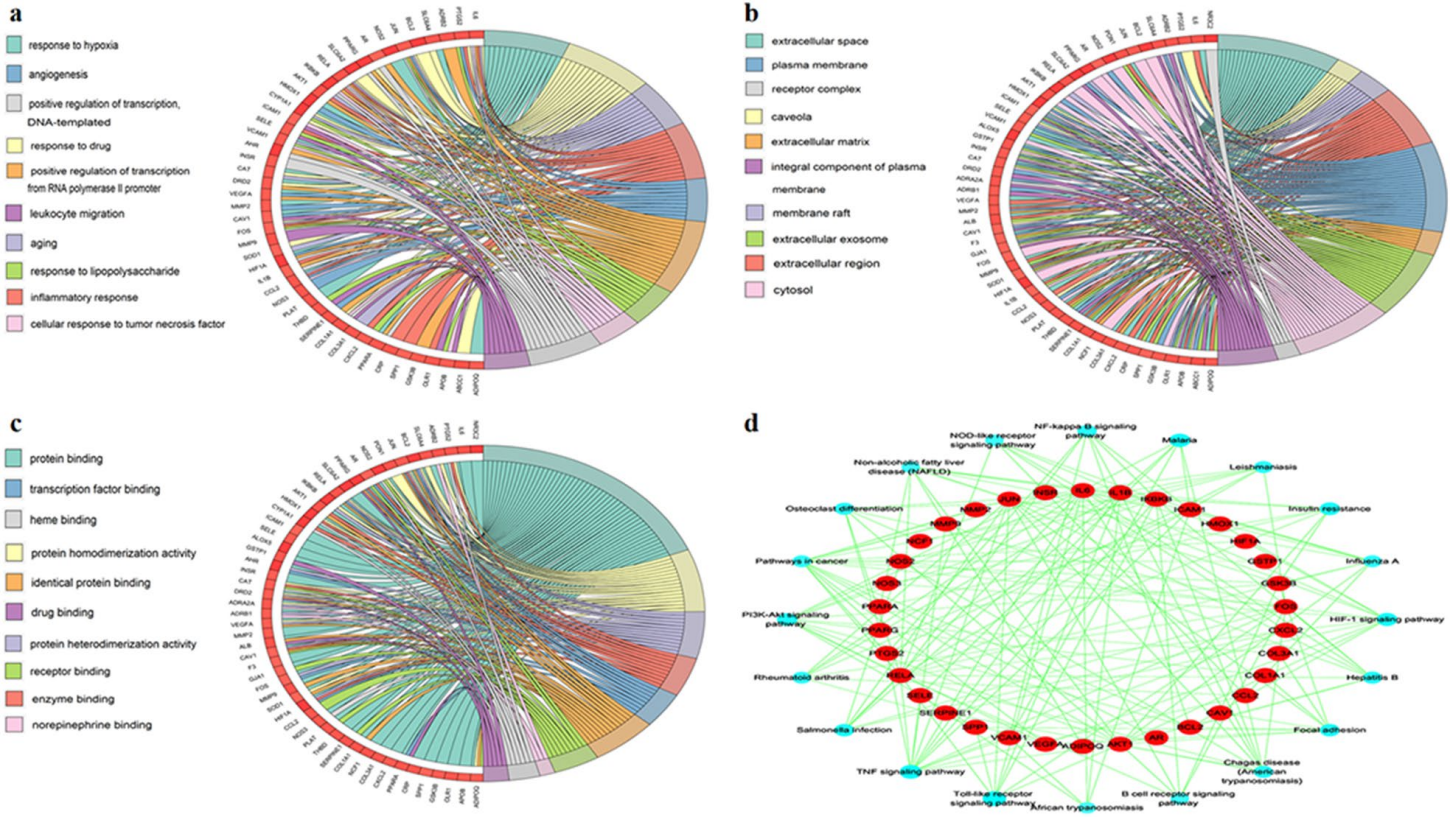
Results of Gene Ontology and Kyoto Encyclopedia of Genes and Genomes (KEGG) enrichment analysis. **a** The top 10 significantly enriched terms in biological process (BP); **b** The top 10 significantly enriched terms in cellular component (CC); **c** The top 10 significantly enriched terms in molecular function (MF); **d** Target–pathway network

To reveal the functional role of the 56 overlapping targets, GO functional analyses were conducted in the DAVID database. The results of GO enrichment analysis included 254 biological processes (BP), 27 cell components (CC), and 35 molecular functions (MF) with a threshold value of p < 0.05. The top 10 GO analysis results are shown in Fig. 4a–c. The BP results mainly comprised of response to hypoxia, response to drug, aging, inflammatory response, and angiogenesis. The CC analysis indicated that the overlapping targets were mainly related to extracellular space, caveola, membrane raft, extracellular region, and plasma membrane. The MF results mainly included protein binding, protein homodimerization activity, protein heterodimerization activity, enzyme binding, and transcription factor binding.

The results of the KEGG enrichment analysis showed that 63 pathways meeting the threshold value of p < 0.05 were significantly enriched. After sorting according to the p-value, the 20 pathways and overlapping targets were used to construct the T-P network using Cytoscape 3.7.1 software. As depicted in Fig. 4d, the KEGG pathways of GZD against hypertension were mainly related to the tumor necrosis factor (TNF) signaling pathway, hypoxia-inducible factor 1 (HIF-1) signaling pathway, Toll-like receptor (TLR) signaling pathway, insulin resistance, PI3K-AKT signaling pathway, and nuclear factor kappa-B (NF-ĸB) signaling pathway.

### Compound–target–pathway network construction

**Fig. 5:**
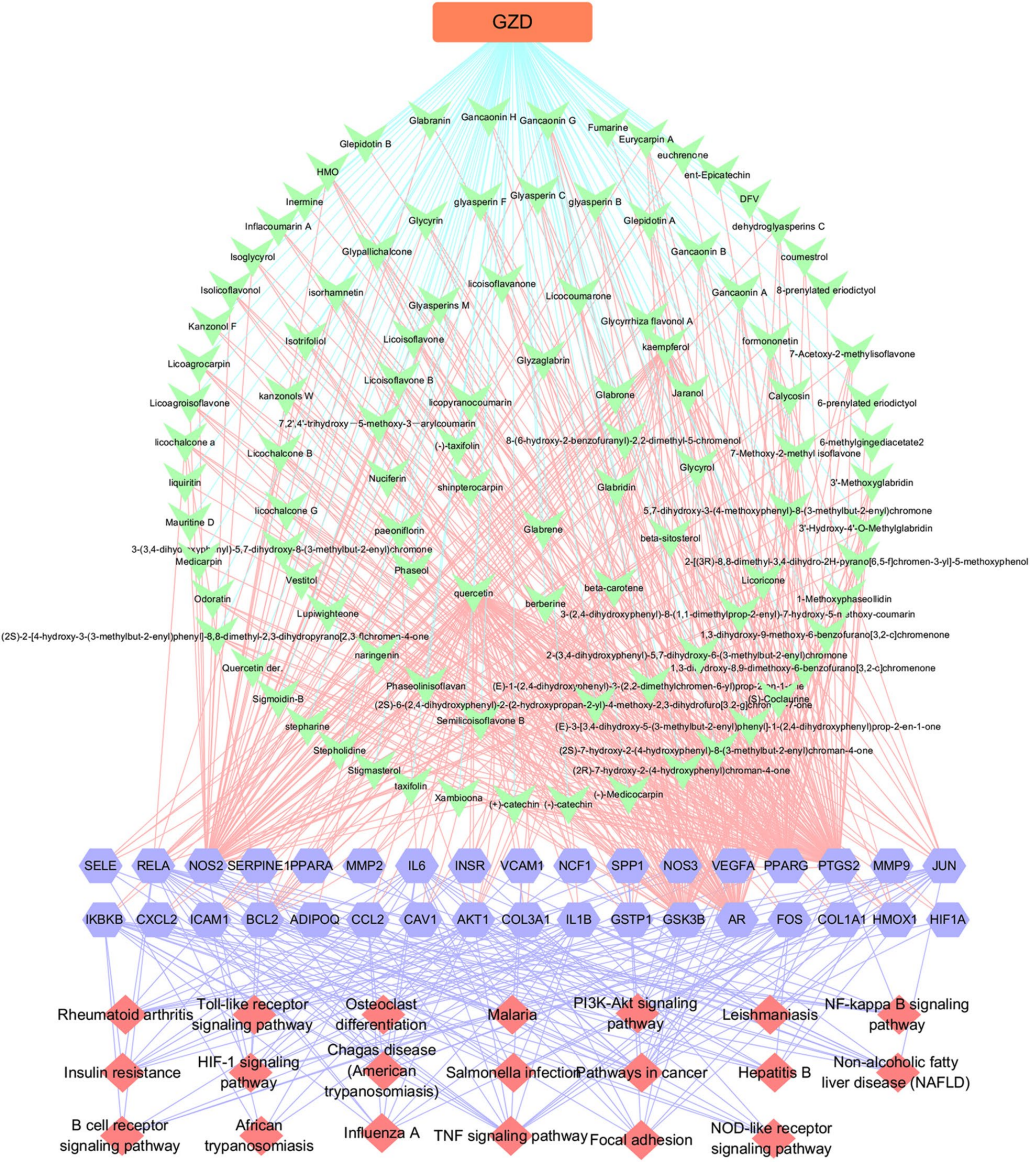
Compound–target–pathway (C–T–P) network of Guizhi decoction (GZD) in the treatment of hypertension. The green node represents the active compound of GZD, the blue node represents the target, the red node represents the pathway

In order to better elucidate the relationship among the GZD, active compounds, targets and pathways, the C–T–P network was further constructed. As shown in Fig. 5, flavonoids, sterols and coumarins were identified as major constituents of GZD for the treatment of hypertension. These are known to exert anti-diabetic, anti-hypertensive, anti-oxidative, lipid-lowering and anti-inflammatory effects. We found that some important flavonoids in GZD, such as kaempferol and quercetin, can act on AKT1, PTGS2, JUN, ICAM1 and PPARG. *β*-sitosterol, stigmasterol and other sterols in GZD can regulate the expression of the PTGS2. Multiple coumarins, such as inflacoumarin A and licopyranocoumarin in GZD can act on PPARG and PTGS2. These results indicated that these active components in GZD can exert their synergistic therapeutic effects through multiple targets and pathways.

### Docking results analysis

**Fig. 6:**
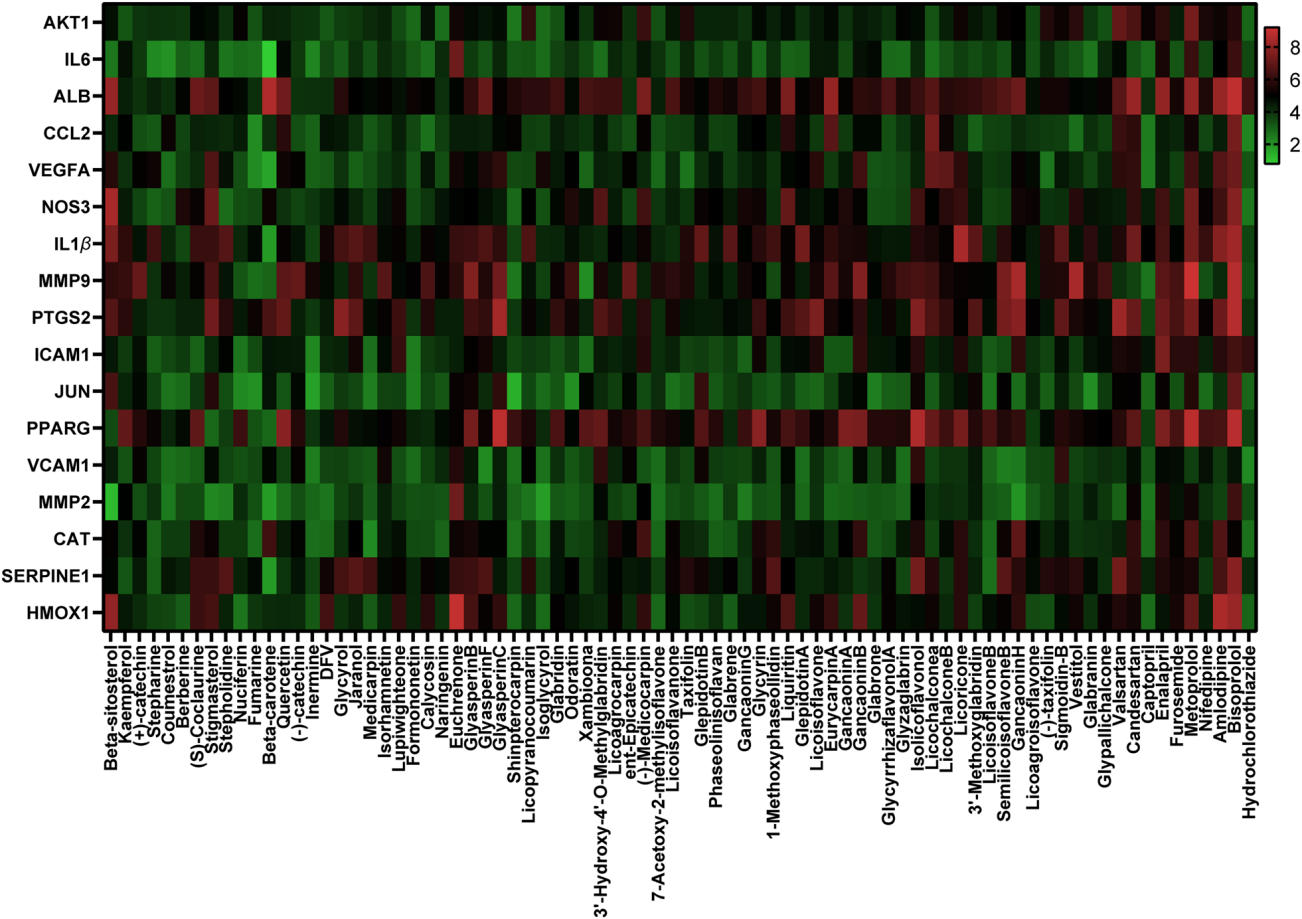
Heat maps of the docking scores for hub targets combined with active compounds in Guizhi decoction (GZD)

**Fig. 7:**
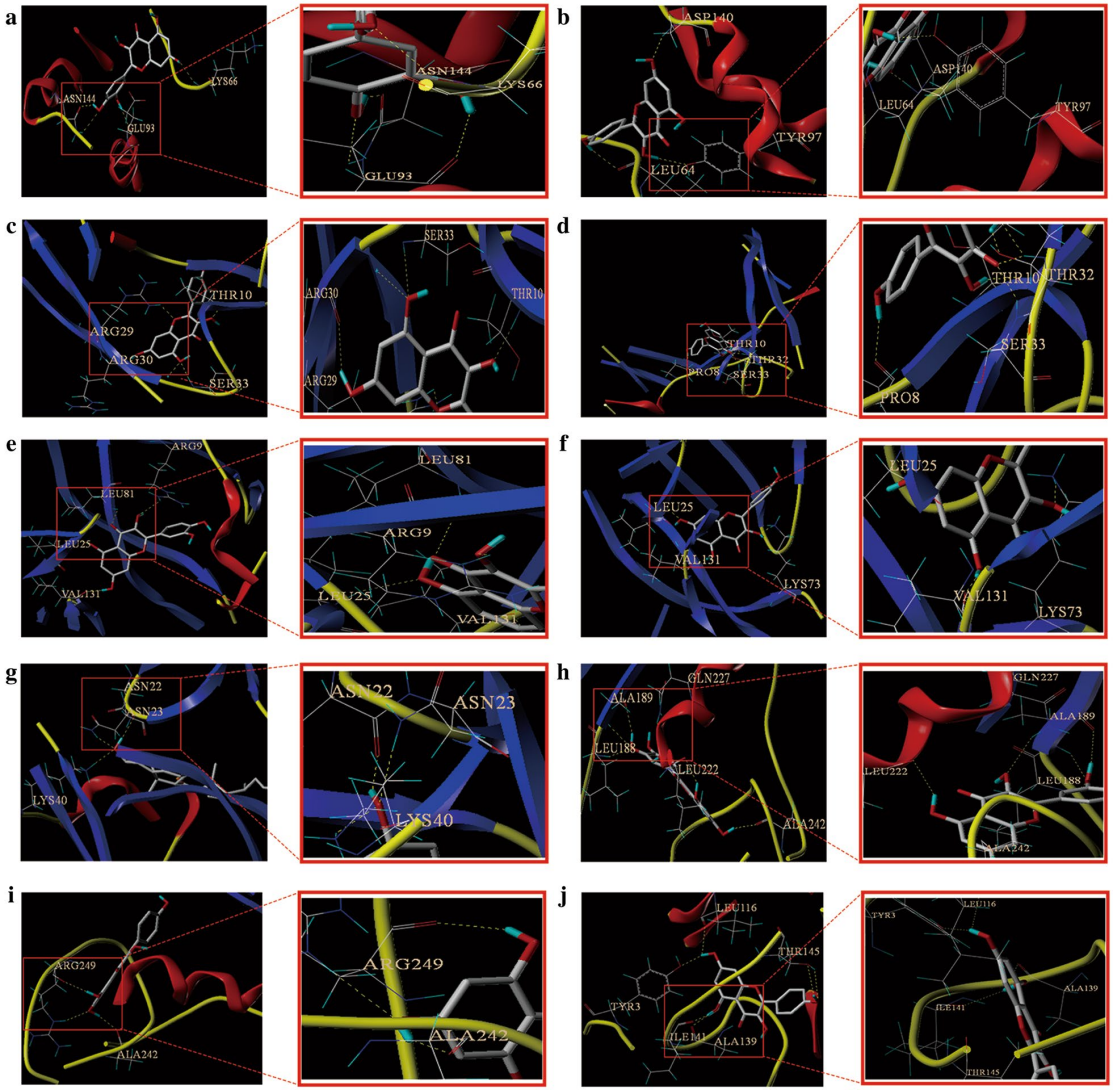
Action modes of active compounds with key targets. **a** Quercetin & IL-6; **b** Kaempferol & IL-6; **c** Quercetin & CCL2; **d** Kaempferol & CCL2; **e** Quercetin & IL-1*β*; **f** Kaempferol & IL-1*β*; **g** *β*-sitosterol & IL-1*β*; **h** Quercetin & MMP-9; **i** Kaempferol & MMP-9; **j** Kaempferol & MMP-2

Docking scores (ranging from 0 to 10) of the 17 key targets are shown in Fig. 6, representing the binding ability from weak to strong. The docking scores and the spatial structure suggested that multiple active compounds of GZD were able to bind tightly to the key targets, which further indicated that the specific therapeutic effect of GZD in the treatment of hypertension, especially for *β*-sitosterol, kaempferol, and quercetin, were very close to the Vina scores of furosemide and nifedipine. These results indicated that quercetin, *β*-sitosterol, and kaempferol are more easier to combine with the key targets and play an effective role in hypertension. As shown in Fig. 7, quercetin, *β*-sitosterol, and kaempferol could bind to the hub targets stably through the analysis of hydrogen bonds and their binding sites. The binding sites and hydrogen bond number were shown in Table 1.

**Table 1:** Docking scores and relevant results of hub targets and key compounds of GZD

Compound	Compound 2D structure	Target	Structure with initial ligand	Total score	Amino acid residue	The number of hydrogen bond
Quercetin		IL-6		4.81	GLU93, LYS66, ASN144	3
Kaempferol		IL-6		4.66	LEU64, ASP140	2
Quercetin		CCL2		5.83	THR10, SER33, ARG30	3
Kaempferol		CCL2		4.95	PRO8, SER33, THR10, THR32	4
Quercetin		IL-1*β*		5.12	LEU24, LEU81	2
Kaempferol		IL-1*β*		5.9	LEU25, VAL131	2
β-sitosterol		IL-1*β*		7.49	ASN22, ASN23, LYS40	3
Quercetin		MMP-9		6.85	LEU188, ALA189, LEU222, ALA242	4
Kaempferol		MMP-9		6.08	ALA242, ARG249	2
Kaempferol		MMP-2		4.94	LEU116, ILE141, THR145	3

### Guizhi decoction improves the blood pressure in rats

**Fig. 8:**
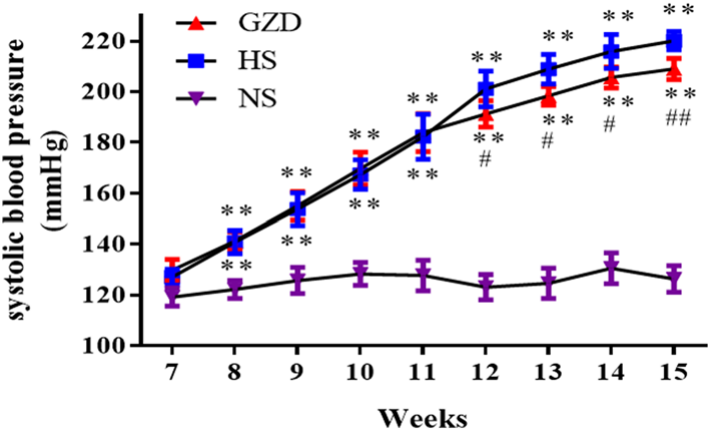
Effects of GZD on blood pressure in Dahl salt-sensitive rats. Data are expressed as mean ± SD (n = 8 rats per group). ^*^P < 0.05, ^**^P < 0.01 compared with the NS group; ^#^*P* < 0.05, ^##^*P* < 0.01 compared with the HS group

As shown in Fig. 8, no obvious differences were observed in the systolic blood pressure (SBP) between the HS group and GZD group before administration of GZD. At 11 weeks of age, 8% NaCl diet significantly increased the SBP in these two groups relative to the NS group(NS: 128 ± 5.99 mmHg, HS: 182 ± 8.86 mmHg, and GZD: 184 ± 7.49 mmHg at 11 weeks; *P* < 0.01). These results indicated that a rat model of hypertension had been successfully established. After 4 weeks of GZD administration, the SBP in the GZD group reduced as compared to the HS group (NS: 126 ± 5.18 mmHg, HS: 220 ± 3.64 mmHg, and GZD: 209 ± 4.17 mmHg at 15 weeks; *P* < 0.01).

### Guizhi decoction decreased the expression of collagen type I and III in rats

**Fig. 9:**
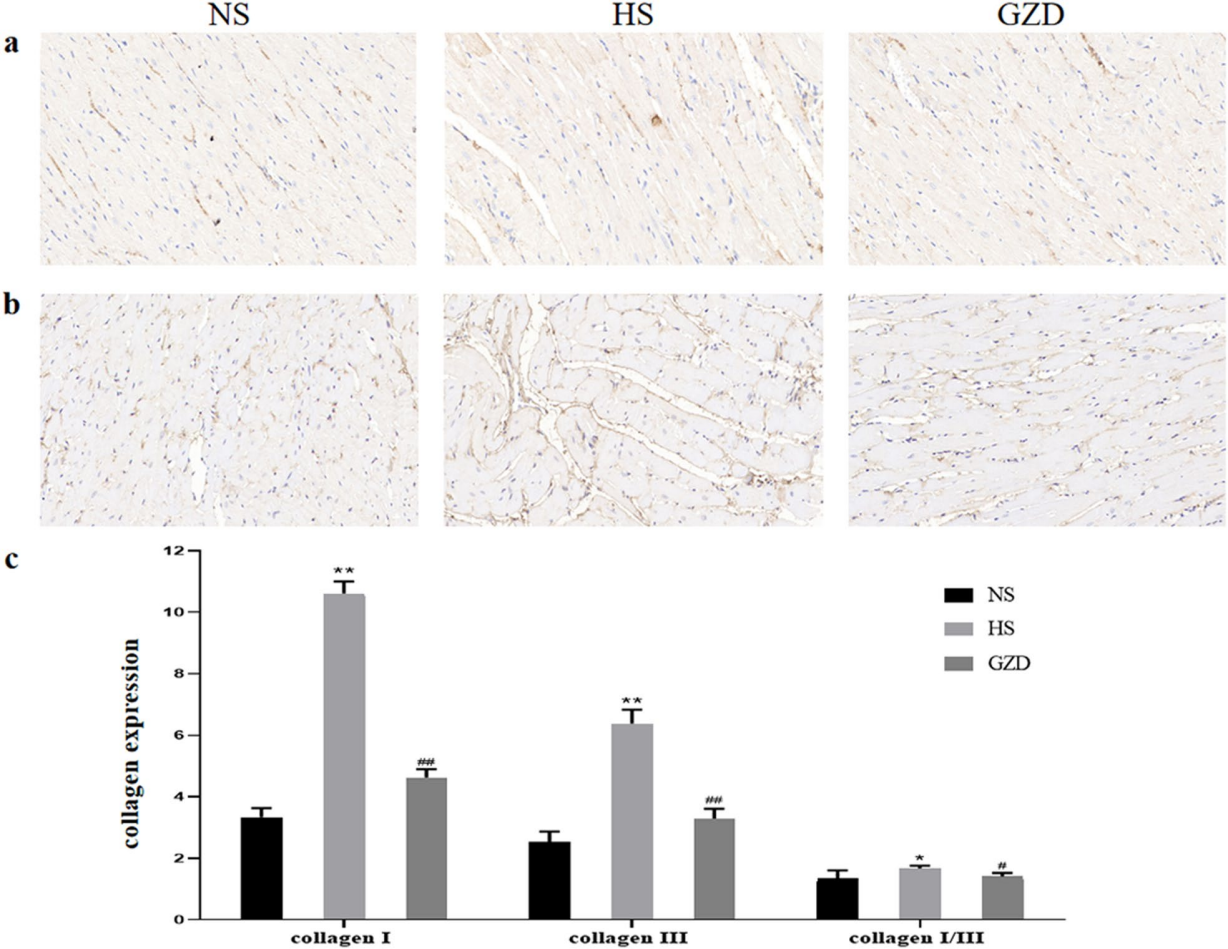
Effects of GZD on the expression levels of collagen type I and III in Dahl salt-sensitive rats. **a** The expression of collagen I (Brown staining) in the left ventricle was detected by immunohistochemical staining. (scale bars: 50 µm). **b** The expression of collagen III in the left ventricle was detected by immunohistochemical staining. (scale bars: 50 µm). **c** Quantitatively analysis of collagen type I and III in the left ventricle. Data are expressed as mean ± SD (n = 8 rats per group). ^*^P < 0.05, ^**^P < 0.01 compared with the NS group; ^#^*P* < 0.05 and ^##^*P* < 0.01 compared with the HS group

As presented in Fig. 9c, the quantitative assessment showed that positive expression rate of collagens I and III were significantly higher in the HS group compared to the NS group ( *P* < 0.01). Treatment with GZD remarkably decreased the increase in positive expression rate of collagens I and III compared to the NS group (*P* < 0.01). Moreover, the collagen I/collagen III ratio was inhibited after GZD treatment (*P* < 0.05), which play a valuable role in the evaluation of myocardial stiffness.

### Guizhi decoction suppressed inflammation in rats

**Fig. 10:**
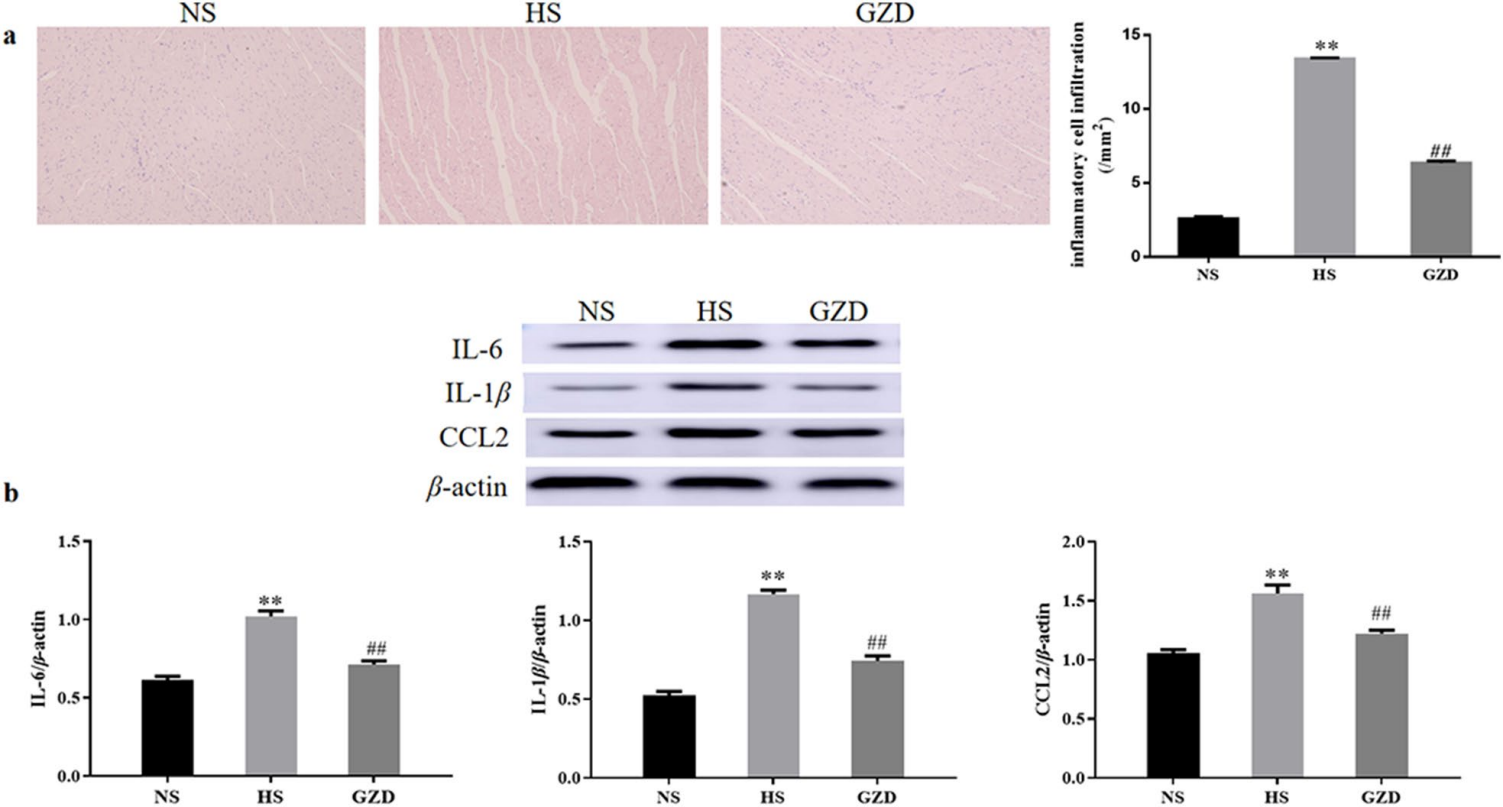
Effects of GZD on the inflammation levels in Dahl salt-sensitive rats. **a** Histomorphological analysis of hematoxylin and eosin (H&E) stained sections (scale bars: 50 µm): Bar graphs show the degree of inflammatory cell infiltration. **b** The protein expression levels of IL-6, CCL2, and IL-1*β* in the left ventricle were evaluated by western blot analysis. Data are expressed as mean ± SD (n = 8 rats per group). ^*^P < 0.05, ^**^P < 0.01 compared with the NS group; ^#^*P* < 0.05, ^##^*P* < 0.01 compared with the HS group

**Fig. 11:**
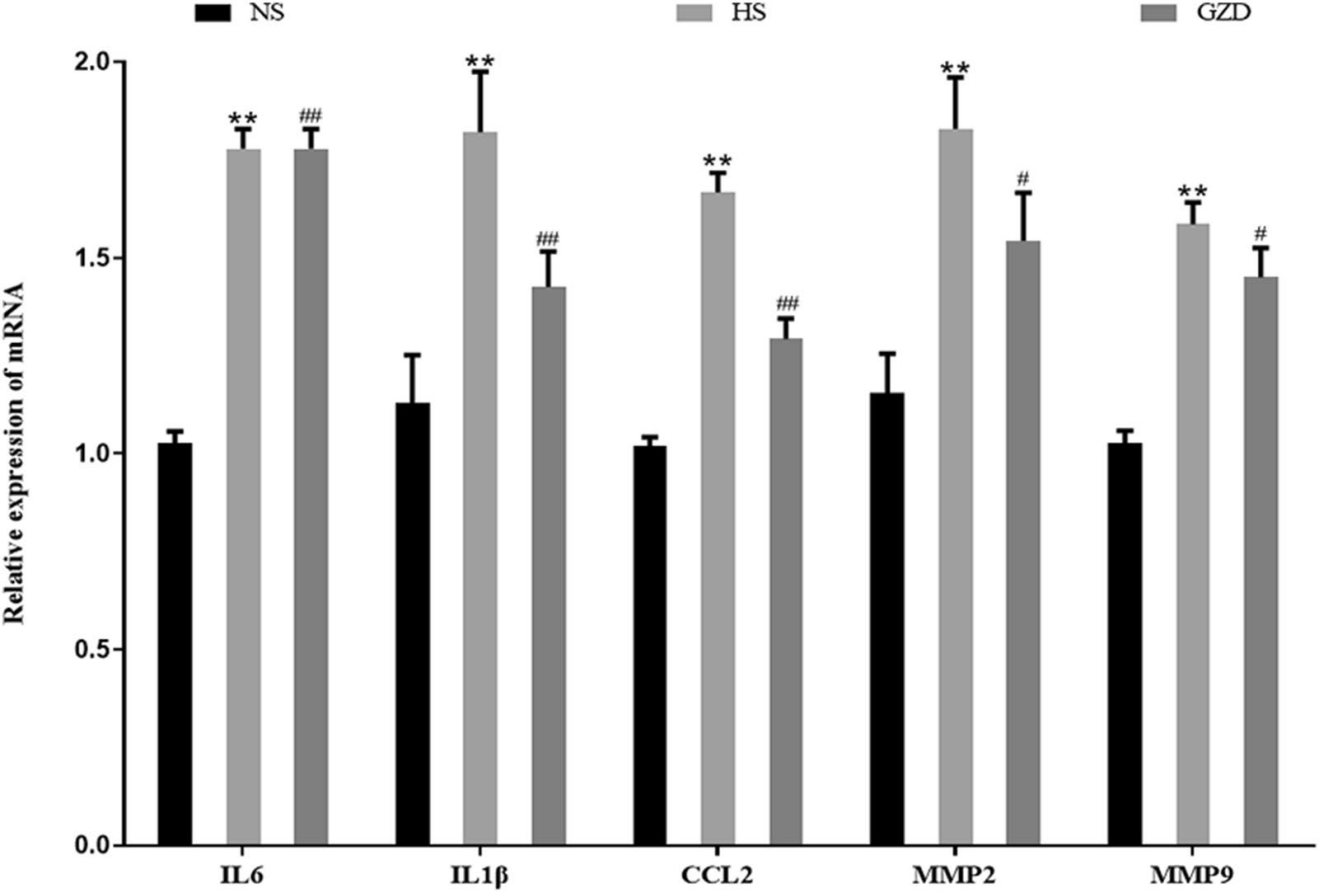
Effects of GZD on the relative mRNA expression levels of IL-6, IL-1*β*,CCL2, MMP-2, and MMP-9 in Dahl salt-sensitive rats. Data are expressed as mean ± SD (n = 8 rats per group). *P < 0.05, ^**^P < 0.01 compared with the NS group; ^#^*P* < 0.05 and ^##^*P* < 0.01 compared with the HS group

As presented in Fig. 10a, H&E staining showed that the ratio of inflammatory cell infiltration in the HS group was remarkably higher than that of the NS group (*P* < 0.01), but this pathological change markedly reduced after administration of GZD (*P* < 0.01). As shown in Figs. 10b and 11, compared with the NS group, the mRNA and protein expressions of IL-6, CCL2, and IL-1*β* in the HS group were significantly increased (*P* < 0.01). After administration of GZD for 4 weeks, the mRNA and protein expressions of IL-6, CCL2, and IL-1*β* in the GZD group were diminished significantly in comparison with the HS group (*P* < 0.01).

### Influence of Guizhi decoction on the myocardial fibrosis in rats

**Fig. 12:**
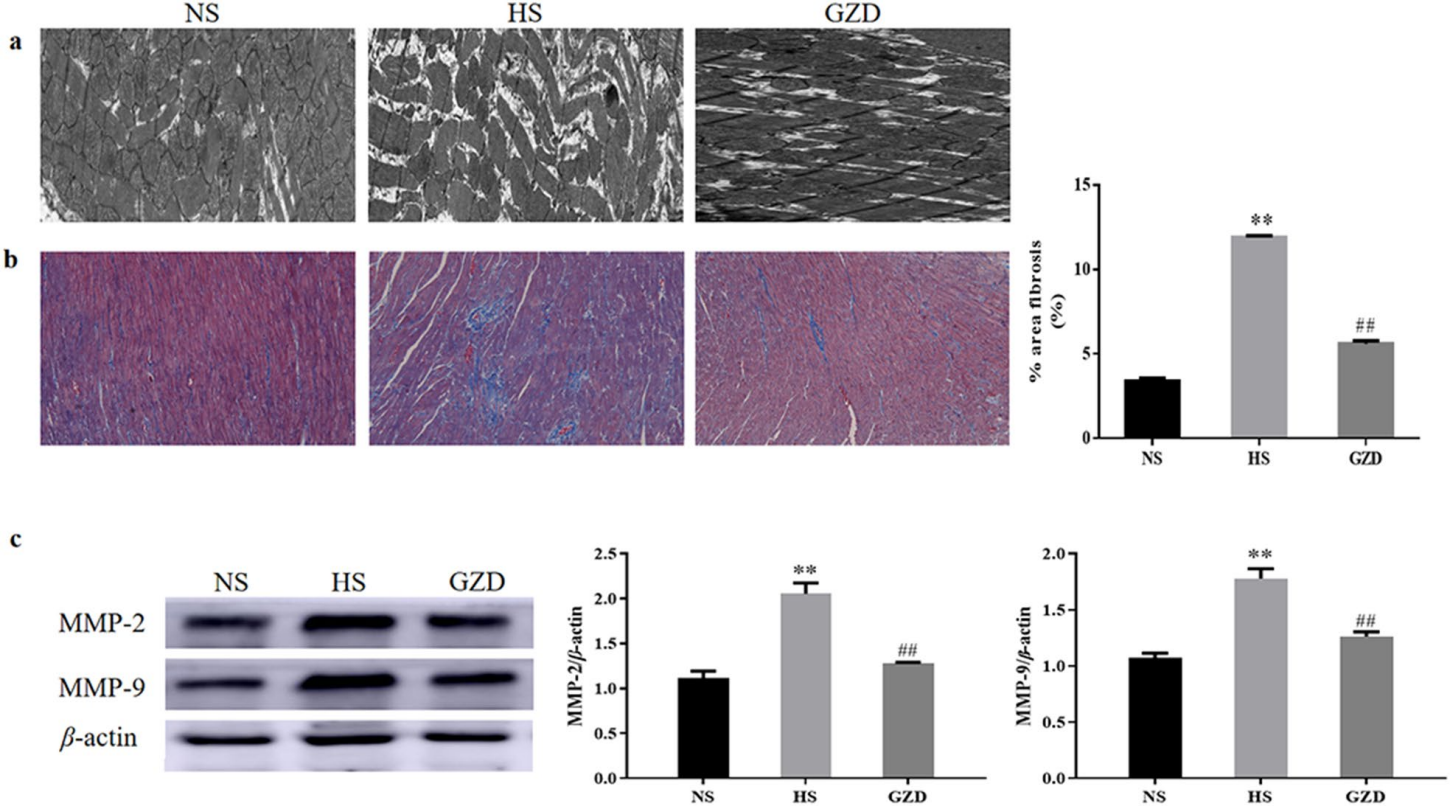
Effects of GZD on the myocardial fibrosis in Dahl salt-sensitive rats. **a** Transmission electron microscopic images of the left ventricle (scale bars: 0.5 µm). **b** Histomorphological analysis of Masson staining: Bar graphs show the percentage area of fibrosis in the left ventricle (scale bars: 50 µm). **c** The protein expression levels of MMP2 and MMP9 in the left ventricle were evaluated by western blot analysis. Data are expressed as mean ± SD (n = 8 rats per group). *P < 0.05, **P < 0.01 compared with the NS group; ^#^*P* < 0.05 and ^##^*P* < 0.01 compared with the HS group

In the results of transmission electron microscopy, the HS group presented disordered arrangement of the cardiomyocytes, apparent myocardial injury, and excessive collagen fibers, which were markedly attenuated with GZD treatment (Fig. 12a). Similarly, the results of Masson staining showed that the degree of interstitial fibrosis decreased in the GZD group as compared to the HS group (Fig. 12b, *P* < 0.01). As shown in Figs. 11 and 12c, the mRNA and protein expressions of MMP-2 and MMP-9 were significantly higher in the HS group as compared to the NS group (*P* < 0.01). After 4 weeks of GZD treatment, the mRNA and protein expressions of MMP-2 and MMP-9 in the GZD group were obviously decreased as compared to the HS group (*P* < 0.05 and *P* < 0.01, respectively), suggesting that GZD could decrease mRNA and protein expressions of MMP-2 and MMP-9 to improve the collagen deposition in Dahl salt-sensitive rats.

## Discussion

Hypertension is a multifactorial disease affected by the complex interactions between genetic predisposition and environmental factors [4]. GZD is wildly used to treat many internal diseases, including allergic or respiratory diseases, endocrine system disease, nervous system disease, and cardiac autonomic neuropathy [25]. Additionally, GZD has accumulated plentiful experiences in the treatment of cardiovascular diseases, such as hypertension [11, 12, 26]. However, its material basis and potential mechanisms have not been fully elucidated. In the present study, we initially identified key compounds, hub targets, and main biological processes and pathways of GZD against hypertension by network pharmacology analysis, and then examined the therapeutic effects of GZD on hypertension in the Dahl salt-sensitive rat model. Our integrative approach elucidated the potential mechanisms of GZD against hypertension based on a systematic network perspective, and demonstrated that GZD can effectively attenuate elevated blood pressure, improve both inflammatory cell infiltration and myocardial fibrosis, and inhibit the expression levels of IL-6, CCL2, IL-1*β*, MMP-2, and MMP-9 in the Dahl salt-sensitive rats.

In this study, 112 active compounds of GZD were identified from the TCMSP database. We constructed a C–T network by mapping 112 active compounds to their 222 corresponding potential targets. In the network, quercetin (degree 272), *β*-sitosterol (degree 104), and kaempferol (degree 102) presented the maximum interactions with potential targets, illustrating that these active compounds with high degree values could play an important role in the potential pharmacological effects of GZD. In order to illustrate the complex relationships among the active compounds, overlapping targets, and pathways, we constructed C–T–D and C–T–P networks. In these networks, quercetin, kaempferol, and *β*-sitosterol exhibited degree values higher than that of other compounds, and these key compounds could all act on multiple targets, such as HMOX1, AKT1, JUN, PPARG, and PTGS2. Moreover, these three key compounds exhibited optimal binding affinity in molecular docking, indicating that they might play a crucial role in the anti-hypertensive effects of GZD. *β*-sitosterol is a type of phytosterol that exerts protective cardiovascular effects by enhancing the intracellular antioxidant defense, improving endothelial function, and inhibiting serum cholesterol levels [27–29]. Kaempferol can effectively maintain blood pressure by repressing the generation of inflammatory cytokines and apoptosis, stimulating the release of NO from the vascular endothelium, and decreasing myocardial fibrosis by inhibiting proliferation of cardiac fibroblasts [30–32]. Quercetin exerts remarkable effects in the treatment of hypertension by improving the endothelial function, inhibiting cardiac fibrosis, and reducing the generation of the adhesion molecules and other inflammatory factors [33–35]. In, addition, we found that beta-carotene, (+)-catechin, formononetin and naringenin may also be the efficacious components of GZD in the treatment of hypertension, but the mechanisms of action need to be further explored.

In the PPI network, we observed that IL-6, CCL2, IL-1*β*, MMP-2, and MMP-9 were the hub targets of GZD for improving hypertension. Notably, these hub targets were closely related to various compounds, biological processes and pathways based on the analysis of multi-layered networks, suggesting that these targets may be important in the role of GZD against hypertension. Several studies have shown that a long-term inflammatory response can trigger sympathetic activation and result in myocardial fibrosis and endothelial dysfunction [36]. Thus, inhibition of the inflammatory response can effectively delay or control the development of hypertension and severe complications [37]. The inflammatory process in hypertension is characterized by increased levels of local inflammatory cytokines such as IL-6, IL-1*β*, TNF-α, and ICAM-1, which are highly correlated with an increased risk for hypertension, and may be useful diagnostic tools for hypertension in the future [4]. Particularly, IL-6, a well-known pro-inflammatory cytokine, participates in the pathological process of hypertension by promoting endothelial dysfunction and inflammatory cell recruitment [38]. Inhibition of IL-1*β* can attenuate the overactivation of RAAS and decrease the overproduction of other pro-inflammatory cytokines, thereby improving hypertension and cardiac fibrosis [39, 40]. CCL2 is a chemokine that contributes to the progression of hypertension by recruiting circulating monocytes to the blood vessel walls and promoting macrophage infiltration [41]. Myocardial fibrosis is a crucial pathological feature in the progression of hypertension. It is predominantly related to the excessive accumulation of extracellular matrix proteins, which contribute to increased ventricular wall stiffness and impaired diastolic function [39, 42]. MMPs participate in the development of myocardial fibrosis by regulating the degradation and production of collagen. Higher levels of MMP-2 and MMP-9 have been considered as markers of cardiovascular risk and aberrant accumulation of collagen [43, 44]. As expected, our experimental results showed that GZD can attenuate the degree of inflammatory infiltration, the area of interstitial fibrosis, and the upregulation of collagen I and collagen III, as well as downregulate the protein and mRNA levels of IL-6, IL-1*β*, CCL2, MMP-9, and MMP-2 in Dahl salt-sensitive rats. These results were consistent with earlier reports showing that improved cardiac inflammation and fibrosis were associated with a decrease in blood pressure [11, 24].

Based on the results of the network analysis and molecular docking, we found that AKT1, VEGFA, eNOS, ICAM-1, PTGS2, and ALB may also be associated with the potential effects of GZD on hypertension. For example, AKT1 directly participates in the phosphorylation of eNOS at serine 1177, which could increase enzyme activity, NO production, and angiogenesis [45]. The vascular endothelium is implicated in the regulation of vascular tone and structure, and abnormal vascular endothelial function may be a major contributor to the adverse outcomes for patients with hypertension [4, 46]. VEGFA is a homologue of the VEGF family, which regulates cell migration, division, and angiogenesis in normal microvascular endothelial cells [47]. Several studies have shown that eNOS significantly contributes to the maintenance of vascular function and cardiovascular homeostasis [48]. NO, produced by eNOS, mediates control of the inflammatory process and regulates neoangiogenesis and vasodilatation. A decrease in NO bioavailability has been implicated as a major cause of endothelial dysfunction in hypertension [4]. ICAM-1 promotes the adhesion of leukocytes and vascular endothelial cells, and subsequently leukocyte activation, which may trigger the endothelial dysfunction, inflammatory response, and blood-vessel remodeling [39, 49]. PTGS2-derived products are related  to the regulation of fluid balance, endothelial function, and ROS production [50]. As a major protein in human serum, the low level of serum ALB is associated with increased risk of hypertension, cardiovascular disease, and carotid atherosclerosis [51]. These results provided preliminary evidence for illuminating the molecular mechanisms of GZD on hypertension through multiple targets.

In the KEGG pathway analysis, overlapping targets were related to multiple pathways, such as TNF signaling pathway, HIF-1 signaling pathway, TLR signaling pathway, insulin resistance, PI3K-AKT signaling pathway, and NF-ĸB signaling pathway. These signaling pathways play a significant role in the pathogenesis and management of hypertension. Activation of the TNF signaling pathway is an important contributor to inflammatory processes, which plays an essential part in modulating the gene expression of cytokines and chemokines involved in vascular inflammation and remodeling [52]. In the inflammatory process associated with hypertension, inappropriate activation of the NF-kB pathway facilitates disease progression by inducing inflammatory cytokine release, vascular dysfunction, and generation of ROS [39, 53] The TLR signaling pathway, a critical upstream mechanism activating inflammatory signaling, regulates inflammatory response by promoting the release of a variety of inflammatory mediators, inducing the migration of immune cells to inflammatory sites, and increasing the adhesion and infiltration ability of inflammatory cells [36, 54]. The HIF-1 pathway is associated with energy metabolism and angiogenesis, and participates in the pathophysiology of inflammation and ischemia. HIF-1 is the chief hypoxia-regulated transcription factor that can regulate cellular responses under hypoxic and ischemic conditions [55, 56]. The PI3K/AKT pathway plays a seminal role in regulating multiple biological effects, including cell growth and proliferation, apoptosis, and angiogenesis [57]. The PI3K/AKT pathway can induce the phosphorylation and activation of eNOS, and regulate blood pressure homeostasis, endothelium function, and vascular integrity [58]. Insulin resistance is a risk factor in patients with hypertension that is closely associated with the activation of RAAS and SNS, resulting in increased peripheral vascular resistance and circulating plasma volume [59]. Therefore, the improvement of insulin resistance is important in the management of hypertension and its complications [60]. However, the specific roles of these signaling pathways in the mechanisms of GZD against hypertension need to be validated through rigorous investigations.

Due to limitations in the database screening conditions and statistical software used in this study, several ingredients and targets of GZD against hypertension may have been missed during the screening process. Therefore, further investigations of these key compounds and pathways in vitro and vivo experiments are required to clarify the efficacy of GZD against hypertension. Despite the limitations of this study, our results revealed the potential mechanisms of GZD against hypertension, and provided scientific basis and valuable enlightenment for guiding future in-depth research and clinical applications.

## Conclusion

In this study, the results of network pharmacology indicated that quercetin, *β*-sitosterol, kaempferol, and other effective compounds of GZD showed therapeutic effects against hypertension via multiple targets and multi-pathways. Furthermore, the experimental results suggested that GZD could downregulate the expression levels of IL-6, IL-1*β*, CCL2, MMP-2, and MMP-9, thus inhibiting the inflammation and myocardial fibrosis in Dahl salt-sensitive rats. In conclusion, this study holistically illuminates the potential mechanisms of GZD against hypertension, and provides scientific basis for further pharmacological studies and clinical applications.

## Supplementary Information

**Additional file 1: Table S1.** The information of the active compounds in GZD.

**Additional file 2: Table S2.** Potential targets of the active compounds in GZD.

## Data Availability

The datasets used and/or analyzed during the current study are available from the corresponding author upon reasonable request.

## Abbreviations

INS: Insulin
REN: Renin
EDN1: Endothelin-1
AGT: Angiotensin
TP53: Cellular tumor antigen p53
ACE: Angiotensin-converting enzyme
FN1: Fibronectin
IGF1: Insulin-like growth factor I
VCAM: Vascular cell adhesion protein
ICAM: Intercellular adhesion molecule
IL-6: Interleukin-6
IL-1B: Interleukin-1B
PTGS2: Prostaglandin G/H synthase 2
TNF: Tumor necrosis factor
MMP: Matrix metalloproteinase
NO: Nitric oxide
eNOS: Endothelial nitric oxide synthase
PI3K: Phosphoinositide 3-kinase
AKT: Serine/threonine-protein kinase
ALB: Albumin
CCL2: C–C motif chemokine 2
VEGFA: Vascular endothelial growth factor A
NF-κB: Nuclear factor kappa-B
HIF: Hypoxia-inducible factor
ROS: Reactive oxygen species
TLR: Toll-like receptor
