# Supplementary material for: Potential mechanisms of Guizhi decoction against hypertension based on network pharmacology and Dahl salt-sensitive rat model
Source: Chin Med. 2021 Apr 27;16:34. doi: 10.1186/s13020-021-00446-x (PMC8077739; doi:10.1186/s13020-021-00446-x)
Supplement: Supplementary file 1 — Additional file 1: Table S1. The information of the active compounds in GZD. [file 13020_2021_446_MOESM1_ESM.doc]

Table S1: The information of the active compounds in GZD

| Mol ID | Molecule name | OB(%) | DL | Herb name |
| --- | --- | --- | --- | --- |
| MOL000492 | [(+)-catechin](https://tcmspw.com/molecule.php?qn=492) | 54.83 | 0.24 | [Cinnamomi Ramulus](https://tcmspw.com/tcmspsearch.php?qr=Cinnamomi%20Ramulus&qsr=herb_en_name&token=27bf73920be4253ff1994b6952217827) |
| MOL000359 | [sitosterol](https://tcmspw.com/molecule.php?qn=359) | 36.91 | 0.75 | [Cinnamomi Ramulus](https://tcmspw.com/tcmspsearch.php?qr=Cinnamomi%20Ramulus&qsr=herb_en_name&token=27bf73920be4253ff1994b6952217827) |
| MOL000358 | [beta-sitosterol](https://tcmspw.com/molecule.php?qn=358) | 36.91 | 0.75 | [Cinnamomi Ramulus](https://tcmspw.com/tcmspsearch.php?qr=Cinnamomi%20Ramulus&qsr=herb_en_name&token=27bf73920be4253ff1994b6952217827) |
| MOL011169 | [Peroxyergosterol](https://tcmspw.com/molecule.php?qn=11169) | 44.39 | 0.82 | [Cinnamomi Ramulus](https://tcmspw.com/tcmspsearch.php?qr=Cinnamomi%20Ramulus&qsr=herb_en_name&token=27bf73920be4253ff1994b6952217827) |
| MOL000073 | [ent-Epicatechin](https://tcmspw.com/molecule.php?qn=73) | 48.96 | 0.24 | [Cinnamomi Ramulus](https://tcmspw.com/tcmspsearch.php?qr=Cinnamomi%20Ramulus&qsr=herb_en_name&token=27bf73920be4253ff1994b6952217827) |
| MOL004576 | [taxifolin](https://tcmspw.com/molecule.php?qn=4576) | 57.84 | 0.27 | [Cinnamomi Ramulus](https://tcmspw.com/tcmspsearch.php?qr=Cinnamomi%20Ramulus&qsr=herb_en_name&token=27bf73920be4253ff1994b6952217827) |
| MOL001736 | [(-)-taxifolin](https://tcmspw.com/molecule.php?qn=1736) | 60.51 | 0.27 | [Cinnamomi Ramulus](https://tcmspw.com/tcmspsearch.php?qr=Cinnamomi%20Ramulus&qsr=herb_en_name&token=27bf73920be4253ff1994b6952217827) |
| MOL001910 | [11alpha,12alpha-epoxy-3beta-23-dihydroxy-30-norolean-20-en-28,12beta-olide](https://tcmspw.com/molecule.php?qn=1910) | 64.77 | 0.38 | [Paeoniae Radix Alba](https://tcmspw.com/tcmspsearch.php?qr=Paeoniae%20Radix%20Alba&qsr=herb_en_name&token=27bf73920be4253ff1994b6952217827) |
| MOL001918 | [paeoniflorgenone](https://tcmspw.com/molecule.php?qn=1918) | 87.59 | 0.37 | [Paeoniae Radix Alba](https://tcmspw.com/tcmspsearch.php?qr=Paeoniae%20Radix%20Alba&qsr=herb_en_name&token=27bf73920be4253ff1994b6952217827) |
| MOL001919 | [(3S,5R,8R,9R,10S,14S)-3,17-dihydroxy-4,4,8,10,14-pentamethyl-2,3,5,6,7,9-hexahydro-1H-cyclopenta[a]phenanthrene-15,16-dione](https://tcmspw.com/molecule.php?qn=1919) | 43.56 | 0.53 | [Paeoniae Radix Alba](https://tcmspw.com/tcmspsearch.php?qr=Paeoniae%20Radix%20Alba&qsr=herb_en_name&token=27bf73920be4253ff1994b6952217827) |
| MOL001921 | [Lactiflorin](https://tcmspw.com/molecule.php?qn=1921) | 49.12 | 0.80 | [Paeoniae Radix Alba](https://tcmspw.com/tcmspsearch.php?qr=Paeoniae%20Radix%20Alba&qsr=herb_en_name&token=27bf73920be4253ff1994b6952217827) |
| MOL001924 | [paeoniflorin](https://tcmspw.com/molecule.php?qn=1924) | 53.87 | 0.79 | [Paeoniae Radix Alba](https://tcmspw.com/tcmspsearch.php?qr=Paeoniae%20Radix%20Alba&qsr=herb_en_name&token=27bf73920be4253ff1994b6952217827) |
| MOL001925 | [paeoniflorin_qt](https://tcmspw.com/molecule.php?qn=1925) | 68.18 | 0.40 | [Paeoniae Radix Alba](https://tcmspw.com/tcmspsearch.php?qr=Paeoniae%20Radix%20Alba&qsr=herb_en_name&token=27bf73920be4253ff1994b6952217827) |
| MOL001928 | [albiflorin_qt](https://tcmspw.com/molecule.php?qn=1928) | 66.64 | 0.33 | [Paeoniae Radix Alba](https://tcmspw.com/tcmspsearch.php?qr=Paeoniae%20Radix%20Alba&qsr=herb_en_name&token=27bf73920be4253ff1994b6952217827) |
| MOL001930 | [benzoyl paeoniflorin](https://tcmspw.com/molecule.php?qn=1930) | 31.27 | 0.75 | [Paeoniae Radix Alba](https://tcmspw.com/tcmspsearch.php?qr=Paeoniae%20Radix%20Alba&qsr=herb_en_name&token=27bf73920be4253ff1994b6952217827) |
| MOL000211 | [Mairin](https://tcmspw.com/molecule.php?qn=211) | 55.38 | 0.78 | [Paeoniae Radix Alba](https://tcmspw.com/tcmspsearch.php?qr=Paeoniae%20Radix%20Alba&qsr=herb_en_name&token=27bf73920be4253ff1994b6952217827) |
| MOL000358 | [beta-sitosterol](https://tcmspw.com/molecule.php?qn=358) | 36.91 | 0.75 | [Paeoniae Radix Alba](https://tcmspw.com/tcmspsearch.php?qr=Paeoniae%20Radix%20Alba&qsr=herb_en_name&token=27bf73920be4253ff1994b6952217827) |
| MOL000359 | [sitosterol](https://tcmspw.com/molecule.php?qn=359) | 36.91 | 0.75 | [Paeoniae Radix Alba](https://tcmspw.com/tcmspsearch.php?qr=Paeoniae%20Radix%20Alba&qsr=herb_en_name&token=27bf73920be4253ff1994b6952217827) |
| MOL000422 | [kaempferol](https://tcmspw.com/molecule.php?qn=422) | 41.88 | 0.24 | [Paeoniae Radix Alba](https://tcmspw.com/tcmspsearch.php?qr=Paeoniae%20Radix%20Alba&qsr=herb_en_name&token=27bf73920be4253ff1994b6952217827) |
| MOL000492 | [(+)-catechin](https://tcmspw.com/molecule.php?qn=492) | 54.83 | 0.24 | [Paeoniae Radix Alba](https://tcmspw.com/tcmspsearch.php?qr=Paeoniae%20Radix%20Alba&qsr=herb_en_name&token=27bf73920be4253ff1994b6952217827) |
| MOL001484 | [Inermine](https://tcmspw.com/molecule.php?qn=1484) | 75.18 | 0.54 | [licorice](https://tcmspw.com/tcmspsearch.php?qr=licorice&qsr=herb_en_name&token=2065e445b3365467877bf499c6a98ad4) |
| MOL001792 | DFV | 32.76 | 0.18 | [licorice](https://tcmspw.com/tcmspsearch.php?qr=licorice&qsr=herb_en_name&token=27bf73920be4253ff1994b6952217827) |
| MOL000211 | [Mairin](https://tcmspw.com/molecule.php?qn=211) | 55.38 | 0.78 | [licorice](https://tcmspw.com/tcmspsearch.php?qr=licorice&qsr=herb_en_name&token=27bf73920be4253ff1994b6952217827) |
| MOL002311 | [Glycyrol](https://tcmspw.com/molecule.php?qn=2311) | 90.78 | 0.67 | [licorice](https://tcmspw.com/tcmspsearch.php?qr=licorice&qsr=herb_en_name&token=27bf73920be4253ff1994b6952217827) |
| MOL000239 | [Jaranol](https://tcmspw.com/molecule.php?qn=239) | 50.83 | 0.29 | [licorice](https://tcmspw.com/tcmspsearch.php?qr=licorice&qsr=herb_en_name&token=27bf73920be4253ff1994b6952217827) |
| MOL002565 | [Medicarpin](https://tcmspw.com/molecule.php?qn=2565) | 49.22 | 0.34 | [licorice](https://tcmspw.com/tcmspsearch.php?qr=licorice&qsr=herb_en_name&token=27bf73920be4253ff1994b6952217827) |
| MOL000354 | [isorhamnetin](https://tcmspw.com/molecule.php?qn=354) | 49.60 | 0.31 | [licorice](https://tcmspw.com/tcmspsearch.php?qr=licorice&qsr=herb_en_name&token=27bf73920be4253ff1994b6952217827) |
| MOL000359 | [sitosterol](https://tcmspw.com/molecule.php?qn=359) | 36.91 | 0.75 | [licorice](https://tcmspw.com/tcmspsearch.php?qr=licorice&qsr=herb_en_name&token=27bf73920be4253ff1994b6952217827) |
| MOL003656 | [Lupiwighteone](https://tcmspw.com/molecule.php?qn=3656) | 51.64 | 0.37 | [licorice](https://tcmspw.com/tcmspsearch.php?qr=licorice&qsr=herb_en_name&token=27bf73920be4253ff1994b6952217827) |
| MOL003896 | [7-Methoxy-2-methyl isoflavone](https://tcmspw.com/molecule.php?qn=3896) | 42.56 | 0.20 | [licorice](https://tcmspw.com/tcmspsearch.php?qr=licorice&qsr=herb_en_name&token=27bf73920be4253ff1994b6952217827) |
| MOL000392 | [formononetin](https://tcmspw.com/molecule.php?qn=392) | 69.67 | 0.21 | [licorice](https://tcmspw.com/tcmspsearch.php?qr=licorice&qsr=herb_en_name&token=27bf73920be4253ff1994b6952217827) |
| MOL000417 | [Calycosin](https://tcmspw.com/molecule.php?qn=417) | 47.75 | 0.24 | [licorice](https://tcmspw.com/tcmspsearch.php?qr=licorice&qsr=herb_en_name&token=27bf73920be4253ff1994b6952217827) |
| MOL000422 | [kaempferol](https://tcmspw.com/molecule.php?qn=422) | 41.88 | 0.24 | [licorice](https://tcmspw.com/tcmspsearch.php?qr=licorice&qsr=herb_en_name&token=27bf73920be4253ff1994b6952217827) |
| MOL004328 | [naringenin](https://tcmspw.com/molecule.php?qn=4328) | 59.29 | 0.21 | [licorice](https://tcmspw.com/tcmspsearch.php?qr=licorice&qsr=herb_en_name&token=27bf73920be4253ff1994b6952217827) |
| MOL004805 | [(2S)-2-[4-hydroxy-3-(3-methylbut-2-enyl)phenyl]-8,8-dimethyl-2,3-dihydropyrano[2,3-f]chromen-4-one](https://tcmspw.com/molecule.php?qn=4805) | 31.79 | 0.72 | [licorice](https://tcmspw.com/tcmspsearch.php?qr=licorice&qsr=herb_en_name&token=27bf73920be4253ff1994b6952217827) |
| MOL004806 | [euchrenone](https://tcmspw.com/molecule.php?qn=4806) | 30.29 | 0.57 | [licorice](https://tcmspw.com/tcmspsearch.php?qr=licorice&qsr=herb_en_name&token=27bf73920be4253ff1994b6952217827) |
| MOL004808 | [glyasperin B](https://tcmspw.com/molecule.php?qn=4808) | 65.22 | 0.44 | [licorice](https://tcmspw.com/tcmspsearch.php?qr=licorice&qsr=herb_en_name&token=27bf73920be4253ff1994b6952217827) |
| MOL004810 | [glyasperin F](https://tcmspw.com/molecule.php?qn=4810) | 75.84 | 0.54 | [licorice](https://tcmspw.com/tcmspsearch.php?qr=licorice&qsr=herb_en_name&token=27bf73920be4253ff1994b6952217827) |
| MOL004811 | [Glyasperin C](https://tcmspw.com/molecule.php?qn=4811) | 45.56 | 0.40 | [licorice](https://tcmspw.com/tcmspsearch.php?qr=licorice&qsr=herb_en_name&token=27bf73920be4253ff1994b6952217827) |
| MOL004814 | [Isotrifoliol](https://tcmspw.com/molecule.php?qn=4814) | 31.94 | 0.42 | [licorice](https://tcmspw.com/tcmspsearch.php?qr=licorice&qsr=herb_en_name&token=27bf73920be4253ff1994b6952217827) |
| MOL004815 | [(E)-1-(2,4-dihydroxyphenyl)-3-(2,2-dimethylchromen-6-yl)prop-2-en-1-one](https://tcmspw.com/molecule.php?qn=4815) | 39.62 | 0.35 | [licorice](https://tcmspw.com/tcmspsearch.php?qr=licorice&qsr=herb_en_name&token=27bf73920be4253ff1994b6952217827) |
| MOL004820 | [kanzonols W](https://tcmspw.com/molecule.php?qn=4820) | 50.48 | 0.52 | [licorice](https://tcmspw.com/tcmspsearch.php?qr=licorice&qsr=herb_en_name&token=27bf73920be4253ff1994b6952217827) |
| MOL004824 | [(2S)-6-(2,4-dihydroxyphenyl)-2-(2-hydroxypropan-2-yl)-4-methoxy-2,3-dihydrofuro[3,2-g]chromen-7-one](https://tcmspw.com/molecule.php?qn=4824) | 60.25 | 0.63 | [licorice](https://tcmspw.com/tcmspsearch.php?qr=licorice&qsr=herb_en_name&token=27bf73920be4253ff1994b6952217827) |
| MOL004827 | [Semilicoisoflavone B](https://tcmspw.com/molecule.php?qn=4827) | 48.78 | 0.55 | [licorice](https://tcmspw.com/tcmspsearch.php?qr=licorice&qsr=herb_en_name&token=27bf73920be4253ff1994b6952217827) |
| MOL004828 | [Glepidotin A](https://tcmspw.com/molecule.php?qn=4828) | 44.72 | 0.35 | [licorice](https://tcmspw.com/tcmspsearch.php?qr=licorice&qsr=herb_en_name&token=27bf73920be4253ff1994b6952217827) |
| MOL004829 | [Glepidotin B](https://tcmspw.com/molecule.php?qn=4829) | 64.46 | 0.34 | [licorice](https://tcmspw.com/tcmspsearch.php?qr=licorice&qsr=herb_en_name&token=27bf73920be4253ff1994b6952217827) |
| MOL004833 | [Phaseolinisoflavan](https://tcmspw.com/molecule.php?qn=4833) | 32.01 | 0.45 | [licorice](https://tcmspw.com/tcmspsearch.php?qr=licorice&qsr=herb_en_name&token=27bf73920be4253ff1994b6952217827) |
| MOL004835 | [Glypallichalcone](https://tcmspw.com/molecule.php?qn=4835) | 61.60 | 0.19 | [licorice](https://tcmspw.com/tcmspsearch.php?qr=licorice&qsr=herb_en_name&token=27bf73920be4253ff1994b6952217827) |
| MOL004838 | [8-(6-hydroxy-2-benzofuranyl)-2,2-dimethyl-5-chromenol](https://tcmspw.com/molecule.php?qn=4838) | 58.44 | 0.38 | [licorice](https://tcmspw.com/tcmspsearch.php?qr=licorice&qsr=herb_en_name&token=27bf73920be4253ff1994b6952217827) |
| MOL004841 | [Licochalcone B](https://tcmspw.com/molecule.php?qn=4841) | 76.76 | 0.19 | [licorice](https://tcmspw.com/tcmspsearch.php?qr=licorice&qsr=herb_en_name&token=27bf73920be4253ff1994b6952217827) |
| MOL004848 | [licochalcone G](https://tcmspw.com/molecule.php?qn=4848) | 49.25 | 0.32 | [licorice](https://tcmspw.com/tcmspsearch.php?qr=licorice&qsr=herb_en_name&token=27bf73920be4253ff1994b6952217827) |
| MOL004849 | [3-(2,4-dihydroxyphenyl)-8-(1,1-dimethylprop-2-enyl)-7-hydroxy-5-methoxy-coumarin](https://tcmspw.com/molecule.php?qn=4849) | 59.62 | 0.43 | [licorice](https://tcmspw.com/tcmspsearch.php?qr=licorice&qsr=herb_en_name&token=27bf73920be4253ff1994b6952217827) |
| MOL004855 | [Licoricone](https://tcmspw.com/molecule.php?qn=4855) | 63.58 | 0.47 | [licorice](https://tcmspw.com/tcmspsearch.php?qr=licorice&qsr=herb_en_name&token=27bf73920be4253ff1994b6952217827) |
| MOL004856 | [Gancaonin A](https://tcmspw.com/molecule.php?qn=4856) | 51.08 | 0.40 | [licorice](https://tcmspw.com/tcmspsearch.php?qr=licorice&qsr=herb_en_name&token=27bf73920be4253ff1994b6952217827) |
| MOL004857 | [Gancaonin B](https://tcmspw.com/molecule.php?qn=4857) | 48.79 | 0.45 | [licorice](https://tcmspw.com/tcmspsearch.php?qr=licorice&qsr=herb_en_name&token=27bf73920be4253ff1994b6952217827) |
| MOL004860 | [licorice glycoside E](https://tcmspw.com/molecule.php?qn=4860) | 32.89 | 0.27 | [licorice](https://tcmspw.com/tcmspsearch.php?qr=licorice&qsr=herb_en_name&token=27bf73920be4253ff1994b6952217827) |
| MOL004863 | [3-(3,4-dihydroxyphenyl)-5,7-dihydroxy-8-(3-methylbut-2-enyl)chromone](https://tcmspw.com/molecule.php?qn=4863) | 66.37 | 0.41 | [licorice](https://tcmspw.com/tcmspsearch.php?qr=licorice&qsr=herb_en_name&token=27bf73920be4253ff1994b6952217827) |
| MOL004864 | [5,7-dihydroxy-3-(4-methoxyphenyl)-8-(3-methylbut-2-enyl)chromone](https://tcmspw.com/molecule.php?qn=4864) | 30.49 | 0.41 | [licorice](https://tcmspw.com/tcmspsearch.php?qr=licorice&qsr=herb_en_name&token=27bf73920be4253ff1994b6952217827) |
| MOL004866 | [2-(3,4-dihydroxyphenyl)-5,7-dihydroxy-6-(3-methylbut-2-enyl)chromone](https://tcmspw.com/molecule.php?qn=4866) | 44.15 | 0.41 | [licorice](https://tcmspw.com/tcmspsearch.php?qr=licorice&qsr=herb_en_name&token=27bf73920be4253ff1994b6952217827) |
| MOL004879 | [Glycyrin](https://tcmspw.com/molecule.php?qn=4879) | 52.61 | 0.47 | [licorice](https://tcmspw.com/tcmspsearch.php?qr=licorice&qsr=herb_en_name&token=27bf73920be4253ff1994b6952217827) |
| MOL004882 | [Licocoumarone](https://tcmspw.com/molecule.php?qn=4882) | 33.21 | 0.36 | [licorice](https://tcmspw.com/tcmspsearch.php?qr=licorice&qsr=herb_en_name&token=27bf73920be4253ff1994b6952217827) |
| MOL004883 | [Licoisoflavone](https://tcmspw.com/molecule.php?qn=4883) | 41.61 | 0.42 | [licorice](https://tcmspw.com/tcmspsearch.php?qr=licorice&qsr=herb_en_name&token=27bf73920be4253ff1994b6952217827) |
| MOL004884 | [Licoisoflavone B](https://tcmspw.com/molecule.php?qn=4884) | 38.93 | 0.55 | [licorice](https://tcmspw.com/tcmspsearch.php?qr=licorice&qsr=herb_en_name&token=27bf73920be4253ff1994b6952217827) |
| MOL004885 | [licoisoflavanone](https://tcmspw.com/molecule.php?qn=4885) | 52.47 | 0.54 | [licorice](https://tcmspw.com/tcmspsearch.php?qr=licorice&qsr=herb_en_name&token=27bf73920be4253ff1994b6952217827) |
| MOL004891 | [shinpterocarpin](https://tcmspw.com/molecule.php?qn=4891) | 80.30 | 0.73 | [licorice](https://tcmspw.com/tcmspsearch.php?qr=licorice&qsr=herb_en_name&token=27bf73920be4253ff1994b6952217827) |
| MOL004898 | [(E)-3-[3,4-dihydroxy-5-(3-methylbut-2-enyl)phenyl]-1-(2,4-dihydroxyphenyl)prop-2-en-1-one](https://tcmspw.com/molecule.php?qn=4898) | 46.27 | 0.31 | [licorice](https://tcmspw.com/tcmspsearch.php?qr=licorice&qsr=herb_en_name&token=27bf73920be4253ff1994b6952217827) |
| MOL004903 | [liquiritin](https://tcmspw.com/molecule.php?qn=4903) | 65.69 | 0.74 | [licorice](https://tcmspw.com/tcmspsearch.php?qr=licorice&qsr=herb_en_name&token=27bf73920be4253ff1994b6952217827) |
| MOL004904 | [licopyranocoumarin](https://tcmspw.com/molecule.php?qn=4904) | 80.36 | 0.65 | [licorice](https://tcmspw.com/tcmspsearch.php?qr=licorice&qsr=herb_en_name&token=27bf73920be4253ff1994b6952217827) |
| MOL004905 | [3,22-Dihydroxy-11-oxo-delta(12)-oleanene-27-alpha-methoxycarbonyl-29-oic acid](https://tcmspw.com/molecule.php?qn=4905) | 34.32 | 0.55 | [licorice](https://tcmspw.com/tcmspsearch.php?qr=licorice&qsr=herb_en_name&token=27bf73920be4253ff1994b6952217827) |
| MOL004907 | [Glyzaglabrin](https://tcmspw.com/molecule.php?qn=4907) | 61.07 | 0.35 | [licorice](https://tcmspw.com/tcmspsearch.php?qr=licorice&qsr=herb_en_name&token=27bf73920be4253ff1994b6952217827) |
| MOL004908 | [Glabridin](https://tcmspw.com/molecule.php?qn=4908) | 53.25 | 0.47 | [licorice](https://tcmspw.com/tcmspsearch.php?qr=licorice&qsr=herb_en_name&token=27bf73920be4253ff1994b6952217827) |
| MOL004910 | [Glabranin](https://tcmspw.com/molecule.php?qn=4910) | 52.90 | 0.31 | [licorice](https://tcmspw.com/tcmspsearch.php?qr=licorice&qsr=herb_en_name&token=27bf73920be4253ff1994b6952217827) |
| MOL004911 | [Glabrene](https://tcmspw.com/molecule.php?qn=4911) | 46.27 | 0.44 | [licorice](https://tcmspw.com/tcmspsearch.php?qr=licorice&qsr=herb_en_name&token=27bf73920be4253ff1994b6952217827) |
| MOL004912 | [Glabrone](https://tcmspw.com/molecule.php?qn=4912) | 52.51 | 0.50 | [licorice](https://tcmspw.com/tcmspsearch.php?qr=licorice&qsr=herb_en_name&token=27bf73920be4253ff1994b6952217827) |
| MOL004913 | [1,3-dihydroxy-9-methoxy-6-benzofurano[3,2-c]chromenone](https://tcmspw.com/molecule.php?qn=4913) | 48.14 | 0.43 | [licorice](https://tcmspw.com/tcmspsearch.php?qr=licorice&qsr=herb_en_name&token=27bf73920be4253ff1994b6952217827) |
| MOL004914 | [1,3-dihydroxy-8,9-dimethoxy-6-benzofurano[3,2-c]chromenone](https://tcmspw.com/molecule.php?qn=4914) | 62.90 | 0.53 | [licorice](https://tcmspw.com/tcmspsearch.php?qr=licorice&qsr=herb_en_name&token=27bf73920be4253ff1994b6952217827) |
| MOL004915 | [Eurycarpin A](https://tcmspw.com/molecule.php?qn=4915) | 43.28 | 0.37 | [licorice](https://tcmspw.com/tcmspsearch.php?qr=licorice&qsr=herb_en_name&token=27bf73920be4253ff1994b6952217827) |
| MOL004917 | [glycyroside](https://tcmspw.com/molecule.php?qn=4917) | 37.25 | 0.79 | [licorice](https://tcmspw.com/tcmspsearch.php?qr=licorice&qsr=herb_en_name&token=27bf73920be4253ff1994b6952217827) |
| MOL004924 | [(-)-Medicocarpin](https://tcmspw.com/molecule.php?qn=4924) | 40.99 | 0.95 | [licorice](https://tcmspw.com/tcmspsearch.php?qr=licorice&qsr=herb_en_name&token=27bf73920be4253ff1994b6952217827) |
| MOL004935 | [Sigmoidin-B](https://tcmspw.com/molecule.php?qn=4935) | 34.88 | 0.41 | [licorice](https://tcmspw.com/tcmspsearch.php?qr=licorice&qsr=herb_en_name&token=27bf73920be4253ff1994b6952217827) |
| MOL004941 | [(2R)-7-hydroxy-2-(4-hydroxyphenyl)chroman-4-one](https://tcmspw.com/molecule.php?qn=4941) | 71.12 | 0.18 | [licorice](https://tcmspw.com/tcmspsearch.php?qr=licorice&qsr=herb_en_name&token=27bf73920be4253ff1994b6952217827) |
| MOL004945 | [(2S)-7-hydroxy-2-(4-hydroxyphenyl)-8-(3-methylbut-2-enyl)chroman-4-one](https://tcmspw.com/molecule.php?qn=4945) | 36.57 | 0.32 | [licorice](https://tcmspw.com/tcmspsearch.php?qr=licorice&qsr=herb_en_name&token=27bf73920be4253ff1994b6952217827) |
| MOL004948 | [Isoglycyrol](https://tcmspw.com/molecule.php?qn=4948) | 44.70 | 0.84 | [licorice](https://tcmspw.com/tcmspsearch.php?qr=licorice&qsr=herb_en_name&token=27bf73920be4253ff1994b6952217827) |
| MOL004949 | [Isolicoflavonol](https://tcmspw.com/molecule.php?qn=4949) | 45.17 | 0.42 | [licorice](https://tcmspw.com/tcmspsearch.php?qr=licorice&qsr=herb_en_name&token=27bf73920be4253ff1994b6952217827) |
| MOL004957 | [HMO](https://tcmspw.com/molecule.php?qn=4957) | 38.37 | 0.21 | [licorice](https://tcmspw.com/tcmspsearch.php?qr=licorice&qsr=herb_en_name&token=27bf73920be4253ff1994b6952217827) |
| MOL004959 | [1-Methoxyphaseollidin](https://tcmspw.com/molecule.php?qn=4959) | 69.98 | 0.64 | [licorice](https://tcmspw.com/tcmspsearch.php?qr=licorice&qsr=herb_en_name&token=27bf73920be4253ff1994b6952217827) |
| MOL004961 | [Quercetin der.](https://tcmspw.com/molecule.php?qn=4961) | 46.45 | 0.33 | [licorice](https://tcmspw.com/tcmspsearch.php?qr=licorice&qsr=herb_en_name&token=27bf73920be4253ff1994b6952217827) |
| MOL004966 | [3'-Hydroxy-4'-O-Methylglabridin](https://tcmspw.com/molecule.php?qn=4966) | 43.71 | 0.57 | [licorice](https://tcmspw.com/tcmspsearch.php?qr=licorice&qsr=herb_en_name&token=27bf73920be4253ff1994b6952217827) |
| MOL000497 | [licochalcone a](https://tcmspw.com/molecule.php?qn=497) | 40.79 | 0.29 | [licorice](https://tcmspw.com/tcmspsearch.php?qr=licorice&qsr=herb_en_name&token=27bf73920be4253ff1994b6952217827) |
| MOL004974 | [3'-Methoxyglabridin](https://tcmspw.com/molecule.php?qn=4974) | 46.16 | 0.57 | [licorice](https://tcmspw.com/tcmspsearch.php?qr=licorice&qsr=herb_en_name&token=27bf73920be4253ff1994b6952217827) |
| MOL004978 | [2-[(3R)-8,8-dimethyl-3,4-dihydro-2H-pyrano[6,5-f]chromen-3-yl]-5-methoxyphenol](https://tcmspw.com/molecule.php?qn=4978) | 36.21 | 0.52 | [licorice](https://tcmspw.com/tcmspsearch.php?qr=licorice&qsr=herb_en_name&token=27bf73920be4253ff1994b6952217827) |
| MOL004980 | [Inflacoumarin A](https://tcmspw.com/molecule.php?qn=4980) | 39.71 | 0.33 | [licorice](https://tcmspw.com/tcmspsearch.php?qr=licorice&qsr=herb_en_name&token=27bf73920be4253ff1994b6952217827) |
| MOL004985 | [icos-5-enoic acid](https://tcmspw.com/molecule.php?qn=4985) | 30.70 | 0.20 | [licorice](https://tcmspw.com/tcmspsearch.php?qr=licorice&qsr=herb_en_name&token=27bf73920be4253ff1994b6952217827) |
| MOL004988 | [Kanzonol F](https://tcmspw.com/molecule.php?qn=4988) | 32.47 | 0.89 | [licorice](https://tcmspw.com/tcmspsearch.php?qr=licorice&qsr=herb_en_name&token=27bf73920be4253ff1994b6952217827) |
| MOL004989 | [6-prenylated eriodictyol](https://tcmspw.com/molecule.php?qn=4989) | 39.22 | 0.41 | [licorice](https://tcmspw.com/tcmspsearch.php?qr=licorice&qsr=herb_en_name&token=27bf73920be4253ff1994b6952217827) |
| MOL004990 | [7,2',4'-trihydroxy－5-methoxy-3－arylcoumarin](https://tcmspw.com/molecule.php?qn=4990) | 83.71 | 0.27 | [licorice](https://tcmspw.com/tcmspsearch.php?qr=licorice&qsr=herb_en_name&token=27bf73920be4253ff1994b6952217827) |
| MOL004991 | [7-Acetoxy-2-methylisoflavone](https://tcmspw.com/molecule.php?qn=4991) | 38.92 | 0.26 | [licorice](https://tcmspw.com/tcmspsearch.php?qr=licorice&qsr=herb_en_name&token=27bf73920be4253ff1994b6952217827) |
| MOL004993 | [8-prenylated eriodictyol](https://tcmspw.com/molecule.php?qn=4993) | 53.79 | 0.40 | [licorice](https://tcmspw.com/tcmspsearch.php?qr=licorice&qsr=herb_en_name&token=27bf73920be4253ff1994b6952217827) |
| MOL004996 | [gadelaidic acid](https://tcmspw.com/molecule.php?qn=4996) | 30.70 | 0.20 | [licorice](https://tcmspw.com/tcmspsearch.php?qr=licorice&qsr=herb_en_name&token=27bf73920be4253ff1994b6952217827) |
| MOL000500 | [Vestitol](https://tcmspw.com/molecule.php?qn=500) | 74.66 | 0.21 | [licorice](https://tcmspw.com/tcmspsearch.php?qr=licorice&qsr=herb_en_name&token=27bf73920be4253ff1994b6952217827) |
| MOL005000 | [Gancaonin G](https://tcmspw.com/molecule.php?qn=5000) | 60.44 | 0.39 | [licorice](https://tcmspw.com/tcmspsearch.php?qr=licorice&qsr=herb_en_name&token=27bf73920be4253ff1994b6952217827) |
| MOL005001 | [Gancaonin H](https://tcmspw.com/molecule.php?qn=5001) | 50.10 | 0.78 | [licorice](https://tcmspw.com/tcmspsearch.php?qr=licorice&qsr=herb_en_name&token=27bf73920be4253ff1994b6952217827) |
| MOL005003 | [Licoagrocarpin](https://tcmspw.com/molecule.php?qn=5003) | 58.81 | 0.58 | [licorice](https://tcmspw.com/tcmspsearch.php?qr=licorice&qsr=herb_en_name&token=27bf73920be4253ff1994b6952217827) |
| MOL005007 | [Glyasperins M](https://tcmspw.com/molecule.php?qn=5007) | 72.67 | 0.59 | [licorice](https://tcmspw.com/tcmspsearch.php?qr=licorice&qsr=herb_en_name&token=27bf73920be4253ff1994b6952217827) |
| MOL005008 | [Glycyrrhiza flavonol A](https://tcmspw.com/molecule.php?qn=5008) | 41.28 | 0.60 | [licorice](https://tcmspw.com/tcmspsearch.php?qr=licorice&qsr=herb_en_name&token=27bf73920be4253ff1994b6952217827) |
| MOL005012 | [Licoagroisoflavone](https://tcmspw.com/molecule.php?qn=5012) | 57.28 | 0.49 | [licorice](https://tcmspw.com/tcmspsearch.php?qr=licorice&qsr=herb_en_name&token=27bf73920be4253ff1994b6952217827) |
| MOL005013 | [18α-hydroxyglycyrrhetic acid](https://tcmspw.com/molecule.php?qn=5013) | 41.16 | 0.71 | [licorice](https://tcmspw.com/tcmspsearch.php?qr=licorice&qsr=herb_en_name&token=27bf73920be4253ff1994b6952217827) |
| MOL005016 | [Odoratin](https://tcmspw.com/molecule.php?qn=5016) | 49.95 | 0.30 | [licorice](https://tcmspw.com/tcmspsearch.php?qr=licorice&qsr=herb_en_name&token=27bf73920be4253ff1994b6952217827) |
| MOL005017 | [Phaseol](https://tcmspw.com/molecule.php?qn=5017) | 78.77 | 0.58 | [licorice](https://tcmspw.com/tcmspsearch.php?qr=licorice&qsr=herb_en_name&token=27bf73920be4253ff1994b6952217827) |
| MOL005018 | [Xambioona](https://tcmspw.com/molecule.php?qn=5018) | 54.85 | 0.87 | [licorice](https://tcmspw.com/tcmspsearch.php?qr=licorice&qsr=herb_en_name&token=27bf73920be4253ff1994b6952217827) |
| MOL005020 | [dehydroglyasperins C](https://tcmspw.com/molecule.php?qn=5020) | 53.82 | 0.37 | [licorice](https://tcmspw.com/tcmspsearch.php?qr=licorice&qsr=herb_en_name&token=27bf73920be4253ff1994b6952217827) |
| MOL000098 | [quercetin](https://tcmspw.com/molecule.php?qn=98) | 46.43 | 0.28 | [licorice](https://tcmspw.com/tcmspsearch.php?qr=licorice&qsr=herb_en_name&token=27bf73920be4253ff1994b6952217827) |
| MOL000358 | [beta-sitosterol](https://tcmspw.com/molecule.php?qn=358) | 36.91 | 0.75 | [Zingiber Officinale Roscoe](https://tcmspw.com/tcmspsearch.php?qr=Zingiber%20Officinale%20Roscoe&qsr=herb_en_name&token=2065e445b3365467877bf499c6a98ad4) |
| MOL006129 | [6-methylgingediacetate2](https://tcmspw.com/molecule.php?qn=6129) | 48.73 | 0.32 | [Zingiber Officinale Roscoe](https://tcmspw.com/tcmspsearch.php?qr=Zingiber%20Officinale%20Roscoe&qsr=herb_en_name&token=2065e445b3365467877bf499c6a98ad4) |
| MOL000449 | [Stigmasterol](https://tcmspw.com/molecule.php?qn=449) | 43.83 | 0.76 | [Zingiber Officinale Roscoe](https://tcmspw.com/tcmspsearch.php?qr=Zingiber%20Officinale%20Roscoe&qsr=herb_en_name&token=27bf73920be4253ff1994b6952217827) |
| MOL001771 | [poriferast-5-en-3beta-ol](https://tcmspw.com/molecule.php?qn=1771) | 36.91 | 0.72 | [Zingiber Officinale Roscoe](https://tcmspw.com/tcmspsearch.php?qr=Zingiber%20Officinale%20Roscoe&qsr=herb_en_name&token=27bf73920be4253ff1994b6952217827) |
| MOL008698 | [Dihydrocapsaicin](https://tcmspw.com/molecule.php?qn=8698) | 47.07 | 0.19 | [Zingiber Officinale Roscoe](https://tcmspw.com/tcmspsearch.php?qr=Zingiber%20Officinale%20Roscoe&qsr=herb_en_name&token=27bf73920be4253ff1994b6952217827) |
| MOL012921 | [stepharine](https://tcmspw.com/molecule.php?qn=12921) | 31.55 | 0.33 | [Jujubae Fructus](https://tcmspw.com/tcmspsearch.php?qr=Jujubae%20Fructus&qsr=herb_en_name&token=2065e445b3365467877bf499c6a98ad4) |
| MOL012940 | [Spiradine A](https://tcmspw.com/molecule.php?qn=12940) | 113.52 | 0.61 | [Jujubae Fructus](https://tcmspw.com/tcmspsearch.php?qr=Jujubae%20Fructus&qsr=herb_en_name&token=2065e445b3365467877bf499c6a98ad4) |
| MOL012946 | [zizyphus saponin I_qt](https://tcmspw.com/molecule.php?qn=12946) | 32.69 | 0.62 | [Jujubae Fructus](https://tcmspw.com/tcmspsearch.php?qr=Jujubae%20Fructus&qsr=herb_en_name&token=2065e445b3365467877bf499c6a98ad4) |
| MOL012961 | [jujuboside A_qt](https://tcmspw.com/molecule.php?qn=12961) | 36.67 | 0.62 | [Jujubae Fructus](https://tcmspw.com/tcmspsearch.php?qr=Jujubae%20Fructus&qsr=herb_en_name&token=2065e445b3365467877bf499c6a98ad4) |
| MOL012976 | [coumestrol](https://tcmspw.com/molecule.php?qn=12976) | 32.49 | 0.34 | [Jujubae Fructus](https://tcmspw.com/tcmspsearch.php?qr=Jujubae%20Fructus&qsr=herb_en_name&token=2065e445b3365467877bf499c6a98ad4) |
| MOL012980 | [Daechuine S6](https://tcmspw.com/molecule.php?qn=12980) | 46.48 | 0.79 | [Jujubae Fructus](https://tcmspw.com/tcmspsearch.php?qr=Jujubae%20Fructus&qsr=herb_en_name&token=2065e445b3365467877bf499c6a98ad4) |
| MOL012981 | [Daechuine S7](https://tcmspw.com/molecule.php?qn=12981) | 44.82 | 0.83 | [Jujubae Fructus](https://tcmspw.com/tcmspsearch.php?qr=Jujubae%20Fructus&qsr=herb_en_name&token=2065e445b3365467877bf499c6a98ad4) |
| MOL012986 | [Jujubasaponin V_qt](https://tcmspw.com/molecule.php?qn=12986) | 36.99 | 0.63 | [Jujubae Fructus](https://tcmspw.com/tcmspsearch.php?qr=Jujubae%20Fructus&qsr=herb_en_name&token=2065e445b3365467877bf499c6a98ad4) |
| MOL012989 | [Jujuboside C_qt](https://tcmspw.com/molecule.php?qn=12989) | 40.26 | 0.62 | [Jujubae Fructus](https://tcmspw.com/tcmspsearch.php?qr=Jujubae%20Fructus&qsr=herb_en_name&token=2065e445b3365467877bf499c6a98ad4) |
| MOL012992 | [Mauritine D](https://tcmspw.com/molecule.php?qn=12992) | 89.13 | 0.45 | [Jujubae Fructus](https://tcmspw.com/tcmspsearch.php?qr=Jujubae%20Fructus&qsr=herb_en_name&token=2065e445b3365467877bf499c6a98ad4) |
| MOL001454 | [berberine](https://tcmspw.com/molecule.php?qn=1454) | 36.86 | 0.78 | [Jujubae Fructus](https://tcmspw.com/tcmspsearch.php?qr=Jujubae%20Fructus&qsr=herb_en_name&token=2065e445b3365467877bf499c6a98ad4) |
| MOL001522 | [(S)-Coclaurine](https://tcmspw.com/molecule.php?qn=1522) | 42.35 | 0.24 | [Jujubae Fructus](https://tcmspw.com/tcmspsearch.php?qr=Jujubae%20Fructus&qsr=herb_en_name&token=2065e445b3365467877bf499c6a98ad4) |
| MOL000211 | [Mairin](https://tcmspw.com/molecule.php?qn=211) | 55.38 | 0.78 | [Jujubae Fructus](https://tcmspw.com/tcmspsearch.php?qr=Jujubae%20Fructus&qsr=herb_en_name&token=2065e445b3365467877bf499c6a98ad4) |
| MOL000449 | [Stigmasterol](https://tcmspw.com/molecule.php?qn=449) | 43.83 | 0.76 | [Jujubae Fructus](https://tcmspw.com/tcmspsearch.php?qr=Jujubae%20Fructus&qsr=herb_en_name&token=2065e445b3365467877bf499c6a98ad4) |
| MOL003410 | [Ziziphin_qt](https://tcmspw.com/molecule.php?qn=3410) | 66.95 | 0.62 | [Jujubae Fructus](https://tcmspw.com/tcmspsearch.php?qr=Jujubae%20Fructus&qsr=herb_en_name&token=2065e445b3365467877bf499c6a98ad4) |
| MOL000358 | [beta-sitosterol](https://tcmspw.com/molecule.php?qn=358) | 36.91 | 0.75 | [Jujubae Fructus](https://tcmspw.com/tcmspsearch.php?qr=Jujubae%20Fructus&qsr=herb_en_name&token=2065e445b3365467877bf499c6a98ad4) |
| MOL004350 | [Ruvoside_qt](https://tcmspw.com/molecule.php?qn=4350) | 36.12 | 0.76 | [Jujubae Fructus](https://tcmspw.com/tcmspsearch.php?qr=Jujubae%20Fructus&qsr=herb_en_name&token=2065e445b3365467877bf499c6a98ad4) |
| MOL000492 | [(+)-catechin](https://tcmspw.com/molecule.php?qn=492) | 54.83 | 0.24 | [Jujubae Fructus](https://tcmspw.com/tcmspsearch.php?qr=Jujubae%20Fructus&qsr=herb_en_name&token=2065e445b3365467877bf499c6a98ad4) |
| MOL005360 | [malkangunin](https://tcmspw.com/molecule.php?qn=5360) | 57.71 | 0.63 | [Jujubae Fructus](https://tcmspw.com/tcmspsearch.php?qr=Jujubae%20Fructus&qsr=herb_en_name&token=2065e445b3365467877bf499c6a98ad4) |
| MOL000627 | [Stepholidine](https://tcmspw.com/molecule.php?qn=627) | 33.11 | 0.54 | [Jujubae Fructus](https://tcmspw.com/tcmspsearch.php?qr=Jujubae%20Fructus&qsr=herb_en_name&token=2065e445b3365467877bf499c6a98ad4) |
| MOL007213 | [Nuciferin](https://tcmspw.com/molecule.php?qn=7213) | 34.43 | 0.40 | [Jujubae Fructus](https://tcmspw.com/tcmspsearch.php?qr=Jujubae%20Fructus&qsr=herb_en_name&token=2065e445b3365467877bf499c6a98ad4) |
| MOL000783 | [Protoporphyrin](https://tcmspw.com/molecule.php?qn=783) | 30.86 | 0.56 | [Jujubae Fructus](https://tcmspw.com/tcmspsearch.php?qr=Jujubae%20Fructus&qsr=herb_en_name&token=2065e445b3365467877bf499c6a98ad4) |
| MOL000787 | [Fumarine](https://tcmspw.com/molecule.php?qn=787) | 59.26 | 0.83 | [Jujubae Fructus](https://tcmspw.com/tcmspsearch.php?qr=Jujubae%20Fructus&qsr=herb_en_name&token=2065e445b3365467877bf499c6a98ad4) |
| MOL008034 | [21302-79-4](https://tcmspw.com/molecule.php?qn=8034) | 73.52 | 0.77 | [Jujubae Fructus](https://tcmspw.com/tcmspsearch.php?qr=Jujubae%20Fructus&qsr=herb_en_name&token=2065e445b3365467877bf499c6a98ad4) |
| MOL008647 | [Moupinamide](https://tcmspw.com/molecule.php?qn=8647) | 86.71 | 0.26 | [Jujubae Fructus](https://tcmspw.com/tcmspsearch.php?qr=Jujubae%20Fructus&qsr=herb_en_name&token=2065e445b3365467877bf499c6a98ad4) |
| MOL002773 | [beta-carotene](https://tcmspw.com/molecule.php?qn=2773) | 37.18 | 0.58 | [Jujubae Fructus](https://tcmspw.com/tcmspsearch.php?qr=Jujubae%20Fructus&qsr=herb_en_name&token=2065e445b3365467877bf499c6a98ad4) |
| MOL000096 | [(-)-catechin](https://tcmspw.com/molecule.php?qn=96) | 49.68 | 0.24 | [Jujubae Fructus](https://tcmspw.com/tcmspsearch.php?qr=Jujubae%20Fructus&qsr=herb_en_name&token=2065e445b3365467877bf499c6a98ad4) |
| MOL000098 | [quercetin](https://tcmspw.com/molecule.php?qn=98) | 46.43 | 0.28 | [Jujubae Fructus](https://tcmspw.com/tcmspsearch.php?qr=Jujubae%20Fructus&qsr=herb_en_name&token=2065e445b3365467877bf499c6a98ad4) |
| MOL013357 | [(3S,6R,8S,9S,10R,13R,14S,17R)-17-[(1R,4R)-4-ethyl-1,5-dimethylhexyl]-10,13-dimethyl-2,3,6,7,8,9,11,12,14,15,16,17-dodecahydro-1H-cyclopenta[a]phenanthrene-3,6-diol](https://tcmspw.com/molecule.php?qn=13357) | 34.37 | 0.78 | [Jujubae Fructus](https://tcmspw.com/tcmspsearch.php?qr=Jujubae%20Fructus&qsr=herb_en_name&token=2065e445b3365467877bf499c6a98ad4) |

Supplementary Table 2: Potential targets of the active compounds in GZD.

| Compound | Target | Gene |  |
| --- | --- | --- | --- |
| (-)-catechin | Prostaglandin G/H synthase 1 | PTGS1 |  |
| (-)-catechin | Estrogen receptor | ESR1 |  |
| (-)-catechin | Prostaglandin G/H synthase 2 | PTGS2 |  |
| (-)-catechin | Nuclear receptor coactivator 2 | NCOA2 |  |
| (-)-catechin | Fatty acid synthase | FASN |  |
| (-)-catechin | Peroxisome proliferator-activated receptor gamma | PPARG |  |
| (-)-catechin | Krueppel-like factor 7 | KLF7 |  |
| (-)-Medicocarpin | Prostaglandin G/H synthase 2 | PTGS2 |  |
| (-)-Medicocarpin | Acetylcholinesterase | ACHE |  |
| (-)-taxifolin | Prostaglandin G/H synthase 1 | PTGS1 |  |
| (-)-taxifolin | Prostaglandin G/H synthase 2 | PTGS2 |  |
| (+)-catechin | Prostaglandin G/H synthase 1 | PTGS1 |  |
| (+)-catechin | Estrogen receptor | ESR1 |  |
| (+)-catechin | Prostaglandin G/H synthase 2 | PTGS2 |  |
| (+)-catechin | Nuclear receptor coactivator 2 | NCOA2 |  |
| (+)-catechin | Retinoic acid receptor RXR-alpha | RXRA |  |
| (+)-catechin | Catalase | CAT |  |
| (+)-catechin | Hyaluronan synthase 2 | HAS2 |  |
| (+)-catechin | Prostaglandin G/H synthase 1 | PTGS1 |  |
| (+)-catechin | Estrogen receptor | ESR1 |  |
| (+)-catechin | Prostaglandin G/H synthase 2 | PTGS2 |  |
| (+)-catechin | Nuclear receptor coactivator 2 | NCOA2 |  |
| (+)-catechin | Retinoic acid receptor RXR-alpha | RXRA |  |
| (+)-catechin | Catalase | CAT |  |
| (+)-catechin | Hyaluronan synthase 2 | HAS2 |  |
| (+)-catechin | Prostaglandin G/H synthase 1 | PTGS1 |  |
| (+)-catechin | Estrogen receptor | ESR1 |  |
| (+)-catechin | Prostaglandin G/H synthase 2 | PTGS2 |  |
| (+)-catechin | Nuclear receptor coactivator 2 | NCOA2 |  |
| (+)-catechin | Retinoic acid receptor RXR-alpha | RXRA |  |
| (+)-catechin | Catalase | CAT |  |
| (+)-catechin | Hyaluronan synthase 2 | HAS2 |  |
| (2R)-7-hydroxy-2-(4-hydroxyphenyl)chroman-4-one | Prostaglandin G/H synthase 1 | PTGS1 |  |
| (2R)-7-hydroxy-2-(4-hydroxyphenyl)chroman-4-one | Estrogen receptor | ESR1 |  |
| (2R)-7-hydroxy-2-(4-hydroxyphenyl)chroman-4-one | Prostaglandin G/H synthase 2 | PTGS2 |  |
| (2R)-7-hydroxy-2-(4-hydroxyphenyl)chroman-4-one | Retinoic acid receptor RXR-alpha | RXRA |  |
| (2R)-7-hydroxy-2-(4-hydroxyphenyl)chroman-4-one | Beta-2 adrenergic receptor | ADRB2 |  |
| (2R)-7-hydroxy-2-(4-hydroxyphenyl)chroman-4-one | Amine oxidase [flavin-containing] B | MAOB |  |
| (2R)-7-hydroxy-2-(4-hydroxyphenyl)chroman-4-one | cAMP-dependent protein kinase inhibitor alpha | PKIA |  |
| (2R)-7-hydroxy-2-(4-hydroxyphenyl)chroman-4-one | Gamma-aminobutyric acid receptor subunit alpha-1 | GABRA1 |  |
| (2R)-7-hydroxy-2-(4-hydroxyphenyl)chroman-4-one | Sodium-dependent serotonin transporter | SLC6A4 |  |
| (2S)-2-[4-hydroxy-3-(3-methylbut-2-enyl)phenyl]-8,8-dimethyl-2,3-dihydropyrano[2,3-f]chromen-4-one | Nitric oxide synthase, inducible | NOS2 |  |
| (2S)-2-[4-hydroxy-3-(3-methylbut-2-enyl)phenyl]-8,8-dimethyl-2,3-dihydropyrano[2,3-f]chromen-4-one | Potassium voltage-gated channel subfamily H member 2 | KCNH2 |  |
| (2S)-2-[4-hydroxy-3-(3-methylbut-2-enyl)phenyl]-8,8-dimethyl-2,3-dihydropyrano[2,3-f]chromen-4-one | Estrogen receptor | ESR1 |  |
| (2S)-2-[4-hydroxy-3-(3-methylbut-2-enyl)phenyl]-8,8-dimethyl-2,3-dihydropyrano[2,3-f]chromen-4-one | Androgen receptor | AR |  |
| (2S)-2-[4-hydroxy-3-(3-methylbut-2-enyl)phenyl]-8,8-dimethyl-2,3-dihydropyrano[2,3-f]chromen-4-one | Peroxisome proliferator activated receptor gamma | PPARG |  |
| (2S)-2-[4-hydroxy-3-(3-methylbut-2-enyl)phenyl]-8,8-dimethyl-2,3-dihydropyrano[2,3-f]chromen-4-one | Prostaglandin G/H synthase 2 | PTGS2 |  |
| (2S)-2-[4-hydroxy-3-(3-methylbut-2-enyl)phenyl]-8,8-dimethyl-2,3-dihydropyrano[2,3-f]chromen-4-one | Estrogen receptor beta | ESR2 |  |
| (2S)-2-[4-hydroxy-3-(3-methylbut-2-enyl)phenyl]-8,8-dimethyl-2,3-dihydropyrano[2,3-f]chromen-4-one | Mitogen-activated protein kinase 14 | MAPK14 |  |
| (2S)-2-[4-hydroxy-3-(3-methylbut-2-enyl)phenyl]-8,8-dimethyl-2,3-dihydropyrano[2,3-f]chromen-4-one | Glycogen synthase kinase-3 beta | GSK3B |  |
| (2S)-6-(2,4-dihydroxyphenyl)-2-(2-hydroxypropan-2-yl)-4-methoxy-2,3-dihydrofuro[3,2-g]chromen-7-one | Nitric oxide synthase, inducible | NOS2 |  |
| (2S)-6-(2,4-dihydroxyphenyl)-2-(2-hydroxypropan-2-yl)-4-methoxy-2,3-dihydrofuro[3,2-g]chromen-7-one | Estrogen receptor | ESR1 |  |
| (2S)-6-(2,4-dihydroxyphenyl)-2-(2-hydroxypropan-2-yl)-4-methoxy-2,3-dihydrofuro[3,2-g]chromen-7-one | Androgen receptor | AR |  |
| (2S)-6-(2,4-dihydroxyphenyl)-2-(2-hydroxypropan-2-yl)-4-methoxy-2,3-dihydrofuro[3,2-g]chromen-7-one | Peroxisome proliferator activated receptor gamma | PPARG |  |
| (2S)-6-(2,4-dihydroxyphenyl)-2-(2-hydroxypropan-2-yl)-4-methoxy-2,3-dihydrofuro[3,2-g]chromen-7-one | Prostaglandin G/H synthase 2 | PTGS2 |  |
| (2S)-6-(2,4-dihydroxyphenyl)-2-(2-hydroxypropan-2-yl)-4-methoxy-2,3-dihydrofuro[3,2-g]chromen-7-one | Coagulation factor VII | F7 |  |
| (2S)-6-(2,4-dihydroxyphenyl)-2-(2-hydroxypropan-2-yl)-4-methoxy-2,3-dihydrofuro[3,2-g]chromen-7-one | Vascular endothelial growth factor receptor 2 | KDR |  |
| (2S)-6-(2,4-dihydroxyphenyl)-2-(2-hydroxypropan-2-yl)-4-methoxy-2,3-dihydrofuro[3,2-g]chromen-7-one | Acetylcholinesterase | ACHE |  |
| (2S)-6-(2,4-dihydroxyphenyl)-2-(2-hydroxypropan-2-yl)-4-methoxy-2,3-dihydrofuro[3,2-g]chromen-7-one | Estrogen receptor beta | ESR2 |  |
| (2S)-6-(2,4-dihydroxyphenyl)-2-(2-hydroxypropan-2-yl)-4-methoxy-2,3-dihydrofuro[3,2-g]chromen-7-one | Mitogen-activated protein kinase 14 | MAPK14 |  |
| (2S)-6-(2,4-dihydroxyphenyl)-2-(2-hydroxypropan-2-yl)-4-methoxy-2,3-dihydrofuro[3,2-g]chromen-7-one | Glycogen synthase kinase-3 beta | GSK3B |  |
| (2S)-6-(2,4-dihydroxyphenyl)-2-(2-hydroxypropan-2-yl)-4-methoxy-2,3-dihydrofuro[3,2-g]chromen-7-one | Serine/threonine-protein kinase Chk1 | CHEK1 |  |
| (2S)-6-(2,4-dihydroxyphenyl)-2-(2-hydroxypropan-2-yl)-4-methoxy-2,3-dihydrofuro[3,2-g]chromen-7-one | Trypsin-1 | PRSS1 |  |
| (2S)-6-(2,4-dihydroxyphenyl)-2-(2-hydroxypropan-2-yl)-4-methoxy-2,3-dihydrofuro[3,2-g]chromen-7-one | Cyclin-A2 | CCNA2 |  |
| (2S)-7-hydroxy-2-(4-hydroxyphenyl)-8-(3-methylbut-2-enyl)chroman-4-one | Nitric oxide synthase, inducible | NOS2 |  |
| (2S)-7-hydroxy-2-(4-hydroxyphenyl)-8-(3-methylbut-2-enyl)chroman-4-one | Prostaglandin G/H synthase 1 | PTGS1 |  |
| (2S)-7-hydroxy-2-(4-hydroxyphenyl)-8-(3-methylbut-2-enyl)chroman-4-one | Estrogen receptor | ESR1 |  |
| (2S)-7-hydroxy-2-(4-hydroxyphenyl)-8-(3-methylbut-2-enyl)chroman-4-one | Sodium channel protein type 5 subunit alpha | SCN5A |  |
| (2S)-7-hydroxy-2-(4-hydroxyphenyl)-8-(3-methylbut-2-enyl)chroman-4-one | Prostaglandin G/H synthase 2 | PTGS2 |  |
| (2S)-7-hydroxy-2-(4-hydroxyphenyl)-8-(3-methylbut-2-enyl)chroman-4-one | Alpha-1B adrenergic receptor | ADRA1B |  |
| (2S)-7-hydroxy-2-(4-hydroxyphenyl)-8-(3-methylbut-2-enyl)chroman-4-one | Beta-2 adrenergic receptor | ADRB2 |  |
| (2S)-7-hydroxy-2-(4-hydroxyphenyl)-8-(3-methylbut-2-enyl)chroman-4-one | Estrogen receptor beta | ESR2 |  |
| (3S,5R,8R,9R,10S,14S)-3,17-dihydroxy-4,4,8,10,14-pentamethyl-2,3,5,6,7,9-hexahydro-1H-cyclopenta[a]phenanthrene-15,16-dione | Progesterone receptor | PGR |  |
| (3S,5R,8R,9R,10S,14S)-3,17-dihydroxy-4,4,8,10,14-pentamethyl-2,3,5,6,7,9-hexahydro-1H-cyclopenta[a]phenanthrene-15,16-dione | Mineralocorticoid receptor | NR3C2 |  |
| (E)-1-(2,4-dihydroxyphenyl)-3-(2,2-dimethylchromen-6-yl)prop-2-en-1-one | Nitric oxide synthase, inducible | NOS2 |  |
| (E)-1-(2,4-dihydroxyphenyl)-3-(2,2-dimethylchromen-6-yl)prop-2-en-1-one | Prostaglandin G/H synthase 1 | PTGS1 |  |
| (E)-1-(2,4-dihydroxyphenyl)-3-(2,2-dimethylchromen-6-yl)prop-2-en-1-one | Estrogen receptor | ESR1 |  |
| (E)-1-(2,4-dihydroxyphenyl)-3-(2,2-dimethylchromen-6-yl)prop-2-en-1-one | Androgen receptor | AR |  |
| (E)-1-(2,4-dihydroxyphenyl)-3-(2,2-dimethylchromen-6-yl)prop-2-en-1-one | Sodium channel protein type 5 subunit alpha | SCN5A |  |
| (E)-1-(2,4-dihydroxyphenyl)-3-(2,2-dimethylchromen-6-yl)prop-2-en-1-one | Peroxisome proliferator activated receptor gamma | PPARG |  |
| (E)-1-(2,4-dihydroxyphenyl)-3-(2,2-dimethylchromen-6-yl)prop-2-en-1-one | Prostaglandin G/H synthase 2 | PTGS2 |  |
| (E)-1-(2,4-dihydroxyphenyl)-3-(2,2-dimethylchromen-6-yl)prop-2-en-1-one | Retinoic acid receptor RXR-alpha | RXRA |  |
| (E)-1-(2,4-dihydroxyphenyl)-3-(2,2-dimethylchromen-6-yl)prop-2-en-1-one | Alpha-1B adrenergic receptor | ADRA1B |  |
| (E)-1-(2,4-dihydroxyphenyl)-3-(2,2-dimethylchromen-6-yl)prop-2-en-1-one | Estrogen receptor beta | ESR2 |  |
| (E)-1-(2,4-dihydroxyphenyl)-3-(2,2-dimethylchromen-6-yl)prop-2-en-1-one | Mitogen-activated protein kinase 14 | MAPK14 |  |
| (E)-1-(2,4-dihydroxyphenyl)-3-(2,2-dimethylchromen-6-yl)prop-2-en-1-one | Glycogen synthase kinase-3 beta | GSK3B |  |
| (E)-1-(2,4-dihydroxyphenyl)-3-(2,2-dimethylchromen-6-yl)prop-2-en-1-one | Serine/threonine-protein kinase Chk1 | CHEK1 |  |
| (E)-1-(2,4-dihydroxyphenyl)-3-(2,2-dimethylchromen-6-yl)prop-2-en-1-one | Cyclin-A2 | CCNA2 |  |
| (E)-1-(2,4-dihydroxyphenyl)-3-(2,2-dimethylchromen-6-yl)prop-2-en-1-one | Nuclear receptor coactivator 2 | NCOA2 |  |
| (E)-3-[3,4-dihydroxy-5-(3-methylbut-2-enyl)phenyl]-1-(2,4-dihydroxyphenyl)prop-2-en-1-one | Estrogen receptor | ESR1 |  |
| (E)-3-[3,4-dihydroxy-5-(3-methylbut-2-enyl)phenyl]-1-(2,4-dihydroxyphenyl)prop-2-en-1-one | Androgen receptor | AR |  |
| (E)-3-[3,4-dihydroxy-5-(3-methylbut-2-enyl)phenyl]-1-(2,4-dihydroxyphenyl)prop-2-en-1-one | Peroxisome proliferator activated receptor gamma | PPARG |  |
| (E)-3-[3,4-dihydroxy-5-(3-methylbut-2-enyl)phenyl]-1-(2,4-dihydroxyphenyl)prop-2-en-1-one | Prostaglandin G/H synthase 2 | PTGS2 |  |
| (E)-3-[3,4-dihydroxy-5-(3-methylbut-2-enyl)phenyl]-1-(2,4-dihydroxyphenyl)prop-2-en-1-one | Mitogen-activated protein kinase 14 | MAPK14 |  |
| (E)-3-[3,4-dihydroxy-5-(3-methylbut-2-enyl)phenyl]-1-(2,4-dihydroxyphenyl)prop-2-en-1-one | Glycogen synthase kinase-3 beta | GSK3B |  |
| (E)-3-[3,4-dihydroxy-5-(3-methylbut-2-enyl)phenyl]-1-(2,4-dihydroxyphenyl)prop-2-en-1-one | Cyclin-A2 | CCNA2 |  |
| (E)-3-[3,4-dihydroxy-5-(3-methylbut-2-enyl)phenyl]-1-(2,4-dihydroxyphenyl)prop-2-en-1-one | Nuclear receptor coactivator 2 | NCOA2 |  |
| (S)-Coclaurine | Prostaglandin G/H synthase 1 | PTGS1 |  |
| (S)-Coclaurine | Muscarinic acetylcholine receptor M1 | CHRM1 |  |
| (S)-Coclaurine | Sodium channel protein type 5 subunit alpha | SCN5A |  |
| (S)-Coclaurine | Prostaglandin G/H synthase 2 | PTGS2 |  |
| (S)-Coclaurine | Retinoic acid receptor RXR-alpha | RXRA |  |
| (S)-Coclaurine | Alpha-1A adrenergic receptor | ADRA1A |  |
| (S)-Coclaurine | Alpha-1B adrenergic receptor | ADRA1B |  |
| (S)-Coclaurine | Sodium-dependent dopamine transporter | SLC6A3 |  |
| (S)-Coclaurine | Beta-2 adrenergic receptor | ADRB2 |  |
| (S)-Coclaurine | Sodium-dependent serotonin transporter | SLC6A4 |  |
| (S)-Coclaurine | Amine oxidase [flavin-containing] B | MAOB |  |
| (S)-Coclaurine | Muscarinic acetylcholine receptor M3 | CHRM3 |  |
| (S)-Coclaurine | Alpha-2A adrenergic receptor | ADRA2A |  |
| (S)-Coclaurine | Alpha-2C adrenergic receptor | ADRA2C |  |
| (S)-Coclaurine | Alpha-1D adrenergic receptor | ADRA1D |  |
| (S)-Coclaurine | Mu-type opioid receptor | OPRM1 |  |
| (S)-Coclaurine | Nuclear receptor coactivator 2 | NCOA2 |  |
| 1,3-dihydroxy-8,9-dimethoxy-6-benzofurano[3,2-c]chromenone | Estrogen receptor | ESR1 |  |
| 1,3-dihydroxy-8,9-dimethoxy-6-benzofurano[3,2-c]chromenone | Androgen receptor | AR |  |
| 1,3-dihydroxy-8,9-dimethoxy-6-benzofurano[3,2-c]chromenone | Peroxisome proliferator activated receptor gamma | PPARG |  |
| 1,3-dihydroxy-8,9-dimethoxy-6-benzofurano[3,2-c]chromenone | Mitogen-activated protein kinase 14 | MAPK14 |  |
| 1,3-dihydroxy-8,9-dimethoxy-6-benzofurano[3,2-c]chromenone | Glycogen synthase kinase-3 beta | GSK3B |  |
| 1,3-dihydroxy-8,9-dimethoxy-6-benzofurano[3,2-c]chromenone | Serine/threonine-protein kinase Chk1 | CHEK1 |  |
| 1,3-dihydroxy-9-methoxy-6-benzofurano[3,2-c]chromenone | Estrogen receptor | ESR1 |  |
| 1,3-dihydroxy-9-methoxy-6-benzofurano[3,2-c]chromenone | Peroxisome proliferator activated receptor gamma | PPARG |  |
| 1,3-dihydroxy-9-methoxy-6-benzofurano[3,2-c]chromenone | Estrogen receptor beta | ESR2 |  |
| 1,3-dihydroxy-9-methoxy-6-benzofurano[3,2-c]chromenone | Mitogen-activated protein kinase 14 | MAPK14 |  |
| 1,3-dihydroxy-9-methoxy-6-benzofurano[3,2-c]chromenone | Glycogen synthase kinase-3 beta | GSK3B |  |
| 1,3-dihydroxy-9-methoxy-6-benzofurano[3,2-c]chromenone | Serine/threonine-protein kinase Chk1 | CHEK1 |  |
| 1,3-dihydroxy-9-methoxy-6-benzofurano[3,2-c]chromenone | Cyclin-A2 | CCNA2 |  |
| 1-Methoxyphaseollidin | Nitric oxide synthase, inducible | NOS2 |  |
| 1-Methoxyphaseollidin | Prostaglandin G/H synthase 1 | PTGS1 |  |
| 1-Methoxyphaseollidin | Potassium voltage-gated channel subfamily H member 2 | KCNH2 |  |
| 1-Methoxyphaseollidin | Estrogen receptor | ESR1 |  |
| 1-Methoxyphaseollidin | Androgen receptor | AR |  |
| 1-Methoxyphaseollidin | Sodium channel protein type 5 subunit alpha | SCN5A |  |
| 1-Methoxyphaseollidin | Peroxisome proliferator activated receptor gamma | PPARG |  |
| 1-Methoxyphaseollidin | Prostaglandin G/H synthase 2 | PTGS2 |  |
| 1-Methoxyphaseollidin | Vascular endothelial growth factor receptor 2 | KDR |  |
| 1-Methoxyphaseollidin | Retinoic acid receptor RXR-alpha | RXRA |  |
| 1-Methoxyphaseollidin | Alpha-1B adrenergic receptor | ADRA1B |  |
| 1-Methoxyphaseollidin | Beta-2 adrenergic receptor | ADRB2 |  |
| 1-Methoxyphaseollidin | Alpha-1D adrenergic receptor | ADRA1D |  |
| 1-Methoxyphaseollidin | Estrogen receptor beta | ESR2 |  |
| 1-Methoxyphaseollidin | Mitogen-activated protein kinase 14 | MAPK14 |  |
| 1-Methoxyphaseollidin | Glycogen synthase kinase-3 beta | GSK3B |  |
| 1-Methoxyphaseollidin | Trypsin-1 | PRSS1 |  |
| 1-Methoxyphaseollidin | Cyclin-A2 | CCNA2 |  |
| 1-Methoxyphaseollidin | Nuclear receptor coactivator 2 | NCOA2 |  |
| 1-Methoxyphaseollidin | Nuclear receptor coactivator 1 | NCOA1 |  |
| 2-(3,4-dihydroxyphenyl)-5,7-dihydroxy-6-(3-methylbut-2-enyl)chromone | Androgen receptor | AR |  |
| 2-(3,4-dihydroxyphenyl)-5,7-dihydroxy-6-(3-methylbut-2-enyl)chromone | Sodium channel protein type 5 subunit alpha | SCN5A |  |
| 2-(3,4-dihydroxyphenyl)-5,7-dihydroxy-6-(3-methylbut-2-enyl)chromone | Peroxisome proliferator activated receptor gamma | PPARG |  |
| 2-(3,4-dihydroxyphenyl)-5,7-dihydroxy-6-(3-methylbut-2-enyl)chromone | Prostaglandin G/H synthase 2 | PTGS2 |  |
| 2-(3,4-dihydroxyphenyl)-5,7-dihydroxy-6-(3-methylbut-2-enyl)chromone | Coagulation factor VII | F7 |  |
| 2-(3,4-dihydroxyphenyl)-5,7-dihydroxy-6-(3-methylbut-2-enyl)chromone | Beta-2 adrenergic receptor | ADRB2 |  |
| 2-(3,4-dihydroxyphenyl)-5,7-dihydroxy-6-(3-methylbut-2-enyl)chromone | Serine/threonine-protein kinase Chk1 | CHEK1 |  |
| 2-(3,4-dihydroxyphenyl)-5,7-dihydroxy-6-(3-methylbut-2-enyl)chromone | Trypsin-1 | PRSS1 |  |
| 2-(3,4-dihydroxyphenyl)-5,7-dihydroxy-6-(3-methylbut-2-enyl)chromone | Cyclin-A2 | CCNA2 |  |
| 2-[(3R)-8,8-dimethyl-3,4-dihydro-2H-pyrano[6,5-f]chromen-3-yl]-5-methoxyphenol | Nitric oxide synthase, inducible | NOS2 |  |
| 2-[(3R)-8,8-dimethyl-3,4-dihydro-2H-pyrano[6,5-f]chromen-3-yl]-5-methoxyphenol | Prostaglandin G/H synthase 1 | PTGS1 |  |
| 2-[(3R)-8,8-dimethyl-3,4-dihydro-2H-pyrano[6,5-f]chromen-3-yl]-5-methoxyphenol | Muscarinic acetylcholine receptor M3 | CHRM3 |  |
| 2-[(3R)-8,8-dimethyl-3,4-dihydro-2H-pyrano[6,5-f]chromen-3-yl]-5-methoxyphenol | Potassium voltage-gated channel subfamily H member 2 | KCNH2 |  |
| 2-[(3R)-8,8-dimethyl-3,4-dihydro-2H-pyrano[6,5-f]chromen-3-yl]-5-methoxyphenol | Muscarinic acetylcholine receptor M1 | CHRM1 |  |
| 2-[(3R)-8,8-dimethyl-3,4-dihydro-2H-pyrano[6,5-f]chromen-3-yl]-5-methoxyphenol | Estrogen receptor | ESR1 |  |
| 2-[(3R)-8,8-dimethyl-3,4-dihydro-2H-pyrano[6,5-f]chromen-3-yl]-5-methoxyphenol | Androgen receptor | AR |  |
| 2-[(3R)-8,8-dimethyl-3,4-dihydro-2H-pyrano[6,5-f]chromen-3-yl]-5-methoxyphenol | Sodium channel protein type 5 subunit alpha | SCN5A |  |
| 2-[(3R)-8,8-dimethyl-3,4-dihydro-2H-pyrano[6,5-f]chromen-3-yl]-5-methoxyphenol | Peroxisome proliferator activated receptor gamma | PPARG |  |
| 2-[(3R)-8,8-dimethyl-3,4-dihydro-2H-pyrano[6,5-f]chromen-3-yl]-5-methoxyphenol | Prostaglandin G/H synthase 2 | PTGS2 |  |
| 2-[(3R)-8,8-dimethyl-3,4-dihydro-2H-pyrano[6,5-f]chromen-3-yl]-5-methoxyphenol | Retinoic acid receptor RXR-alpha | RXRA |  |
| 2-[(3R)-8,8-dimethyl-3,4-dihydro-2H-pyrano[6,5-f]chromen-3-yl]-5-methoxyphenol | Acetylcholinesterase | ACHE |  |
| 2-[(3R)-8,8-dimethyl-3,4-dihydro-2H-pyrano[6,5-f]chromen-3-yl]-5-methoxyphenol | Alpha-1B adrenergic receptor | ADRA1B |  |
| 2-[(3R)-8,8-dimethyl-3,4-dihydro-2H-pyrano[6,5-f]chromen-3-yl]-5-methoxyphenol | Sodium-dependent dopamine transporter | SLC6A3 |  |
| 2-[(3R)-8,8-dimethyl-3,4-dihydro-2H-pyrano[6,5-f]chromen-3-yl]-5-methoxyphenol | Beta-2 adrenergic receptor | ADRB2 |  |
| 2-[(3R)-8,8-dimethyl-3,4-dihydro-2H-pyrano[6,5-f]chromen-3-yl]-5-methoxyphenol | Estrogen receptor beta | ESR2 |  |
| 2-[(3R)-8,8-dimethyl-3,4-dihydro-2H-pyrano[6,5-f]chromen-3-yl]-5-methoxyphenol | Mitogen-activated protein kinase 14 | MAPK14 |  |
| 2-[(3R)-8,8-dimethyl-3,4-dihydro-2H-pyrano[6,5-f]chromen-3-yl]-5-methoxyphenol | Glycogen synthase kinase-3 beta | GSK3B |  |
| 2-[(3R)-8,8-dimethyl-3,4-dihydro-2H-pyrano[6,5-f]chromen-3-yl]-5-methoxyphenol | Serine/threonine-protein kinase Chk1 | CHEK1 |  |
| 2-[(3R)-8,8-dimethyl-3,4-dihydro-2H-pyrano[6,5-f]chromen-3-yl]-5-methoxyphenol | Retinoic acid receptor RXR-beta | RXRB |  |
| 2-[(3R)-8,8-dimethyl-3,4-dihydro-2H-pyrano[6,5-f]chromen-3-yl]-5-methoxyphenol | Trypsin-1 | PRSS1 |  |
| 2-[(3R)-8,8-dimethyl-3,4-dihydro-2H-pyrano[6,5-f]chromen-3-yl]-5-methoxyphenol | Cyclin-A2 | CCNA2 |  |
| 2-[(3R)-8,8-dimethyl-3,4-dihydro-2H-pyrano[6,5-f]chromen-3-yl]-5-methoxyphenol | Nuclear receptor coactivator 2 | NCOA2 |  |
| 2-[(3R)-8,8-dimethyl-3,4-dihydro-2H-pyrano[6,5-f]chromen-3-yl]-5-methoxyphenol | Nuclear receptor coactivator 1 | NCOA1 |  |
| 3-(2,4-dihydroxyphenyl)-8-(1,1-dimethylprop-2-enyl)-7-hydroxy-5-methoxy-coumarin | Nitric oxide synthase, inducible | NOS2 |  |
| 3-(2,4-dihydroxyphenyl)-8-(1,1-dimethylprop-2-enyl)-7-hydroxy-5-methoxy-coumarin | Potassium voltage-gated channel subfamily H member 2 | KCNH2 |  |
| 3-(2,4-dihydroxyphenyl)-8-(1,1-dimethylprop-2-enyl)-7-hydroxy-5-methoxy-coumarin | Estrogen receptor | ESR1 |  |
| 3-(2,4-dihydroxyphenyl)-8-(1,1-dimethylprop-2-enyl)-7-hydroxy-5-methoxy-coumarin | Androgen receptor | AR |  |
| 3-(2,4-dihydroxyphenyl)-8-(1,1-dimethylprop-2-enyl)-7-hydroxy-5-methoxy-coumarin | Peroxisome proliferator activated receptor gamma | PPARG |  |
| 3-(2,4-dihydroxyphenyl)-8-(1,1-dimethylprop-2-enyl)-7-hydroxy-5-methoxy-coumarin | Prostaglandin G/H synthase 2 | PTGS2 |  |
| 3-(2,4-dihydroxyphenyl)-8-(1,1-dimethylprop-2-enyl)-7-hydroxy-5-methoxy-coumarin | Coagulation factor VII | F7 |  |
| 3-(2,4-dihydroxyphenyl)-8-(1,1-dimethylprop-2-enyl)-7-hydroxy-5-methoxy-coumarin | Vascular endothelial growth factor receptor 2 | KDR |  |
| 3-(2,4-dihydroxyphenyl)-8-(1,1-dimethylprop-2-enyl)-7-hydroxy-5-methoxy-coumarin | Estrogen receptor beta | ESR2 |  |
| 3-(2,4-dihydroxyphenyl)-8-(1,1-dimethylprop-2-enyl)-7-hydroxy-5-methoxy-coumarin | Mitogen-activated protein kinase 14 | MAPK14 |  |
| 3-(2,4-dihydroxyphenyl)-8-(1,1-dimethylprop-2-enyl)-7-hydroxy-5-methoxy-coumarin | Glycogen synthase kinase-3 beta | GSK3B |  |
| 3-(2,4-dihydroxyphenyl)-8-(1,1-dimethylprop-2-enyl)-7-hydroxy-5-methoxy-coumarin | Serine/threonine-protein kinase Chk1 | CHEK1 |  |
| 3-(2,4-dihydroxyphenyl)-8-(1,1-dimethylprop-2-enyl)-7-hydroxy-5-methoxy-coumarin | Trypsin-1 | PRSS1 |  |
| 3-(2,4-dihydroxyphenyl)-8-(1,1-dimethylprop-2-enyl)-7-hydroxy-5-methoxy-coumarin | Nuclear receptor coactivator 2 | NCOA2 |  |
| 3-(2,4-dihydroxyphenyl)-8-(1,1-dimethylprop-2-enyl)-7-hydroxy-5-methoxy-coumarin | Nuclear receptor coactivator 1 | NCOA1 |  |
| 3-(3,4-dihydroxyphenyl)-5,7-dihydroxy-8-(3-methylbut-2-enyl)chromone | Nitric oxide synthase, inducible | NOS2 |  |
| 3-(3,4-dihydroxyphenyl)-5,7-dihydroxy-8-(3-methylbut-2-enyl)chromone | Estrogen receptor | ESR1 |  |
| 3-(3,4-dihydroxyphenyl)-5,7-dihydroxy-8-(3-methylbut-2-enyl)chromone | Androgen receptor | AR |  |
| 3-(3,4-dihydroxyphenyl)-5,7-dihydroxy-8-(3-methylbut-2-enyl)chromone | Peroxisome proliferator activated receptor gamma | PPARG |  |
| 3-(3,4-dihydroxyphenyl)-5,7-dihydroxy-8-(3-methylbut-2-enyl)chromone | Prostaglandin G/H synthase 2 | PTGS2 |  |
| 3-(3,4-dihydroxyphenyl)-5,7-dihydroxy-8-(3-methylbut-2-enyl)chromone | Mitogen-activated protein kinase 14 | MAPK14 |  |
| 3-(3,4-dihydroxyphenyl)-5,7-dihydroxy-8-(3-methylbut-2-enyl)chromone | Glycogen synthase kinase-3 beta | GSK3B |  |
| 3-(3,4-dihydroxyphenyl)-5,7-dihydroxy-8-(3-methylbut-2-enyl)chromone | Serine/threonine-protein kinase Chk1 | CHEK1 |  |
| 3-(3,4-dihydroxyphenyl)-5,7-dihydroxy-8-(3-methylbut-2-enyl)chromone | Trypsin-1 | PRSS1 |  |
| 3-(3,4-dihydroxyphenyl)-5,7-dihydroxy-8-(3-methylbut-2-enyl)chromone | Cyclin-A2 | CCNA2 |  |
| 3-(3,4-dihydroxyphenyl)-5,7-dihydroxy-8-(3-methylbut-2-enyl)chromone | Nuclear receptor coactivator 2 | NCOA2 |  |
| 3'-Hydroxy-4'-O-Methylglabridin | Nitric oxide synthase, inducible | NOS2 |  |
| 3'-Hydroxy-4'-O-Methylglabridin | Prostaglandin G/H synthase 1 | PTGS1 |  |
| 3'-Hydroxy-4'-O-Methylglabridin | Potassium voltage-gated channel subfamily H member 2 | KCNH2 |  |
| 3'-Hydroxy-4'-O-Methylglabridin | Estrogen receptor | ESR1 |  |
| 3'-Hydroxy-4'-O-Methylglabridin | Androgen receptor | AR |  |
| 3'-Hydroxy-4'-O-Methylglabridin | Sodium channel protein type 5 subunit alpha | SCN5A |  |
| 3'-Hydroxy-4'-O-Methylglabridin | Peroxisome proliferator activated receptor gamma | PPARG |  |
| 3'-Hydroxy-4'-O-Methylglabridin | Prostaglandin G/H synthase 2 | PTGS2 |  |
| 3'-Hydroxy-4'-O-Methylglabridin | Coagulation factor VII | F7 |  |
| 3'-Hydroxy-4'-O-Methylglabridin | Vascular endothelial growth factor receptor 2 | KDR |  |
| 3'-Hydroxy-4'-O-Methylglabridin | Alpha-1B adrenergic receptor | ADRA1B |  |
| 3'-Hydroxy-4'-O-Methylglabridin | Beta-2 adrenergic receptor | ADRB2 |  |
| 3'-Hydroxy-4'-O-Methylglabridin | Estrogen receptor beta | ESR2 |  |
| 3'-Hydroxy-4'-O-Methylglabridin | Mitogen-activated protein kinase 14 | MAPK14 |  |
| 3'-Hydroxy-4'-O-Methylglabridin | Glycogen synthase kinase-3 beta | GSK3B |  |
| 3'-Hydroxy-4'-O-Methylglabridin | Serine/threonine-protein kinase Chk1 | CHEK1 |  |
| 3'-Hydroxy-4'-O-Methylglabridin | Trypsin-1 | PRSS1 |  |
| 3'-Hydroxy-4'-O-Methylglabridin | Cyclin-A2 | CCNA2 |  |
| 3'-Hydroxy-4'-O-Methylglabridin | Nuclear receptor coactivator 2 | NCOA2 |  |
| 3'-Hydroxy-4'-O-Methylglabridin | Nuclear receptor coactivator 1 | NCOA1 |  |
| 3'-Methoxyglabridin | Nitric oxide synthase, inducible | NOS2 |  |
| 3'-Methoxyglabridin | Prostaglandin G/H synthase 1 | PTGS1 |  |
| 3'-Methoxyglabridin | Potassium voltage-gated channel subfamily H member 2 | KCNH2 |  |
| 3'-Methoxyglabridin | Estrogen receptor | ESR1 |  |
| 3'-Methoxyglabridin | Androgen receptor | AR |  |
| 3'-Methoxyglabridin | Sodium channel protein type 5 subunit alpha | SCN5A |  |
| 3'-Methoxyglabridin | Peroxisome proliferator activated receptor gamma | PPARG |  |
| 3'-Methoxyglabridin | Prostaglandin G/H synthase 2 | PTGS2 |  |
| 3'-Methoxyglabridin | Coagulation factor VII | F7 |  |
| 3'-Methoxyglabridin | Retinoic acid receptor RXR-alpha | RXRA |  |
| 3'-Methoxyglabridin | Acetylcholinesterase | ACHE |  |
| 3'-Methoxyglabridin | Alpha-1B adrenergic receptor | ADRA1B |  |
| 3'-Methoxyglabridin | Beta-2 adrenergic receptor | ADRB2 |  |
| 3'-Methoxyglabridin | Estrogen receptor beta | ESR2 |  |
| 3'-Methoxyglabridin | Mitogen-activated protein kinase 14 | MAPK14 |  |
| 3'-Methoxyglabridin | Glycogen synthase kinase-3 beta | GSK3B |  |
| 3'-Methoxyglabridin | Serine/threonine-protein kinase Chk1 | CHEK1 |  |
| 3'-Methoxyglabridin | Trypsin-1 | PRSS1 |  |
| 3'-Methoxyglabridin | Cyclin-A2 | CCNA2 |  |
| 3'-Methoxyglabridin | Nuclear receptor coactivator 2 | NCOA2 |  |
| 3'-Methoxyglabridin | Nuclear receptor coactivator 1 | NCOA1 |  |
| 5,7-dihydroxy-3-(4-methoxyphenyl)-8-(3-methylbut-2-enyl)chromone | Nitric oxide synthase, inducible | NOS2 |  |
| 5,7-dihydroxy-3-(4-methoxyphenyl)-8-(3-methylbut-2-enyl)chromone | Potassium voltage-gated channel subfamily H member 2 | KCNH2 |  |
| 5,7-dihydroxy-3-(4-methoxyphenyl)-8-(3-methylbut-2-enyl)chromone | Estrogen receptor | ESR1 |  |
| 5,7-dihydroxy-3-(4-methoxyphenyl)-8-(3-methylbut-2-enyl)chromone | Androgen receptor | AR |  |
| 5,7-dihydroxy-3-(4-methoxyphenyl)-8-(3-methylbut-2-enyl)chromone | Peroxisome proliferator activated receptor gamma | PPARG |  |
| 5,7-dihydroxy-3-(4-methoxyphenyl)-8-(3-methylbut-2-enyl)chromone | Prostaglandin G/H synthase 2 | PTGS2 |  |
| 5,7-dihydroxy-3-(4-methoxyphenyl)-8-(3-methylbut-2-enyl)chromone | Estrogen receptor beta | ESR2 |  |
| 5,7-dihydroxy-3-(4-methoxyphenyl)-8-(3-methylbut-2-enyl)chromone | Mitogen-activated protein kinase 14 | MAPK14 |  |
| 5,7-dihydroxy-3-(4-methoxyphenyl)-8-(3-methylbut-2-enyl)chromone | Glycogen synthase kinase-3 beta | GSK3B |  |
| 5,7-dihydroxy-3-(4-methoxyphenyl)-8-(3-methylbut-2-enyl)chromone | Serine/threonine-protein kinase Chk1 | CHEK1 |  |
| 5,7-dihydroxy-3-(4-methoxyphenyl)-8-(3-methylbut-2-enyl)chromone | Trypsin-1 | PRSS1 |  |
| 5,7-dihydroxy-3-(4-methoxyphenyl)-8-(3-methylbut-2-enyl)chromone | Cyclin-A2 | CCNA2 |  |
| 5,7-dihydroxy-3-(4-methoxyphenyl)-8-(3-methylbut-2-enyl)chromone | Nuclear receptor coactivator 2 | NCOA2 |  |
| 6-methylgingediacetate2 | Estrogen receptor | ESR1 |  |
| 6-methylgingediacetate2 | Prostaglandin G/H synthase 2 | PTGS2 |  |
| 6-prenylated eriodictyol | Nitric oxide synthase, inducible | NOS2 |  |
| 6-prenylated eriodictyol | Estrogen receptor | ESR1 |  |
| 6-prenylated eriodictyol | Sodium channel protein type 5 subunit alpha | SCN5A |  |
| 6-prenylated eriodictyol | Prostaglandin G/H synthase 2 | PTGS2 |  |
| 6-prenylated eriodictyol | Coagulation factor VII | F7 |  |
| 7,2',4'-trihydroxy－5-methoxy-3－arylcoumarin | Nitric oxide synthase, inducible | NOS2 |  |
| 7,2',4'-trihydroxy－5-methoxy-3－arylcoumarin | Prostaglandin G/H synthase 1 | PTGS1 |  |
| 7,2',4'-trihydroxy－5-methoxy-3－arylcoumarin | Estrogen receptor | ESR1 |  |
| 7,2',4'-trihydroxy－5-methoxy-3－arylcoumarin | Androgen receptor | AR |  |
| 7,2',4'-trihydroxy－5-methoxy-3－arylcoumarin | Peroxisome proliferator activated receptor gamma | PPARG |  |
| 7,2',4'-trihydroxy－5-methoxy-3－arylcoumarin | Prostaglandin G/H synthase 2 | PTGS2 |  |
| 7,2',4'-trihydroxy－5-methoxy-3－arylcoumarin | Estrogen receptor beta | ESR2 |  |
| 7,2',4'-trihydroxy－5-methoxy-3－arylcoumarin | Mitogen-activated protein kinase 14 | MAPK14 |  |
| 7,2',4'-trihydroxy－5-methoxy-3－arylcoumarin | Glycogen synthase kinase-3 beta | GSK3B |  |
| 7,2',4'-trihydroxy－5-methoxy-3－arylcoumarin | Serine/threonine-protein kinase Chk1 | CHEK1 |  |
| 7-Acetoxy-2-methylisoflavone | Nitric oxide synthase, inducible | NOS2 |  |
| 7-Acetoxy-2-methylisoflavone | Prostaglandin G/H synthase 1 | PTGS1 |  |
| 7-Acetoxy-2-methylisoflavone | Estrogen receptor | ESR1 |  |
| 7-Acetoxy-2-methylisoflavone | Androgen receptor | AR |  |
| 7-Acetoxy-2-methylisoflavone | Sodium channel protein type 5 subunit alpha | SCN5A |  |
| 7-Acetoxy-2-methylisoflavone | Peroxisome proliferator activated receptor gamma | PPARG |  |
| 7-Acetoxy-2-methylisoflavone | Prostaglandin G/H synthase 2 | PTGS2 |  |
| 7-Acetoxy-2-methylisoflavone | Retinoic acid receptor RXR-alpha | RXRA |  |
| 7-Acetoxy-2-methylisoflavone | Acetylcholinesterase | ACHE |  |
| 7-Acetoxy-2-methylisoflavone | Alpha-1B adrenergic receptor | ADRA1B |  |
| 7-Acetoxy-2-methylisoflavone | Beta-2 adrenergic receptor | ADRB2 |  |
| 7-Acetoxy-2-methylisoflavone | Alpha-1D adrenergic receptor | ADRA1D |  |
| 7-Acetoxy-2-methylisoflavone | Gamma-aminobutyric acid receptor subunit alpha-1 | GABRA1 |  |
| 7-Acetoxy-2-methylisoflavone | Mitogen-activated protein kinase 14 | MAPK14 |  |
| 7-Acetoxy-2-methylisoflavone | Glycogen synthase kinase-3 beta | GSK3B |  |
| 7-Acetoxy-2-methylisoflavone | Serine/threonine-protein kinase Chk1 | CHEK1 |  |
| 7-Acetoxy-2-methylisoflavone | Trypsin-1 | PRSS1 |  |
| 7-Acetoxy-2-methylisoflavone | Nuclear receptor coactivator 2 | NCOA2 |  |
| 7-Methoxy-2-methyl isoflavone | Nitric oxide synthase, inducible | NOS2 |  |
| 7-Methoxy-2-methyl isoflavone | Prostaglandin G/H synthase 1 | PTGS1 |  |
| 7-Methoxy-2-methyl isoflavone | Muscarinic acetylcholine receptor M3 | CHRM3 |  |
| 7-Methoxy-2-methyl isoflavone | Muscarinic acetylcholine receptor M1 | CHRM1 |  |
| 7-Methoxy-2-methyl isoflavone | Estrogen receptor | ESR1 |  |
| 7-Methoxy-2-methyl isoflavone | Androgen receptor | AR |  |
| 7-Methoxy-2-methyl isoflavone | Beta-1 adrenergic receptor | ADRB1 |  |
| 7-Methoxy-2-methyl isoflavone | Sodium channel protein type 5 subunit alpha | SCN5A |  |
| 7-Methoxy-2-methyl isoflavone | Peroxisome proliferator activated receptor gamma | PPARG |  |
| 7-Methoxy-2-methyl isoflavone | Prostaglandin G/H synthase 2 | PTGS2 |  |
| 7-Methoxy-2-methyl isoflavone | Retinoic acid receptor RXR-alpha | RXRA |  |
| 7-Methoxy-2-methyl isoflavone | Acetylcholinesterase | ACHE |  |
| 7-Methoxy-2-methyl isoflavone | Alpha-1B adrenergic receptor | ADRA1B |  |
| 7-Methoxy-2-methyl isoflavone | Sodium-dependent dopamine transporter | SLC6A3 |  |
| 7-Methoxy-2-methyl isoflavone | Beta-2 adrenergic receptor | ADRB2 |  |
| 7-Methoxy-2-methyl isoflavone | Alpha-1D adrenergic receptor | ADRA1D |  |
| 7-Methoxy-2-methyl isoflavone | Sodium-dependent serotonin transporter | SLC6A4 |  |
| 7-Methoxy-2-methyl isoflavone | Estrogen receptor beta | ESR2 |  |
| 7-Methoxy-2-methyl isoflavone | Gamma-aminobutyric acid receptor subunit alpha-1 | GABRA1 |  |
| 7-Methoxy-2-methyl isoflavone | Mitogen-activated protein kinase 14 | MAPK14 |  |
| 7-Methoxy-2-methyl isoflavone | Glycogen synthase kinase-3 beta | GSK3B |  |
| 7-Methoxy-2-methyl isoflavone | Leukotriene A-4 hydrolase | LTA4H |  |
| 7-Methoxy-2-methyl isoflavone | Amine oxidase [flavin-containing] B | MAOB |  |
| 7-Methoxy-2-methyl isoflavone | Serine/threonine-protein kinase Chk1 | CHEK1 |  |
| 7-Methoxy-2-methyl isoflavone | Trypsin-1 | PRSS1 |  |
| 7-Methoxy-2-methyl isoflavone | Cyclin-A2 | CCNA2 |  |
| 7-Methoxy-2-methyl isoflavone | Nuclear receptor coactivator 1 | NCOA1 |  |
| 7-Methoxy-2-methyl isoflavone | cAMP-dependent protein kinase inhibitor alpha | PKIA |  |
| 7-Methoxy-2-methyl isoflavone | Muscarinic acetylcholine receptor M5 | CHRM5 |  |
| 7-Methoxy-2-methyl isoflavone | Mu-type opioid receptor | OPRM1 |  |
| 7-Methoxy-2-methyl isoflavone | Nuclear receptor coactivator 2 | NCOA2 |  |
| 8-(6-hydroxy-2-benzofuranyl)-2,2-dimethyl-5-chromenol | Nitric oxide synthase, inducible | NOS2 |  |
| 8-(6-hydroxy-2-benzofuranyl)-2,2-dimethyl-5-chromenol | Estrogen receptor | ESR1 |  |
| 8-(6-hydroxy-2-benzofuranyl)-2,2-dimethyl-5-chromenol | Prostaglandin G/H synthase 2 | PTGS2 |  |
| 8-(6-hydroxy-2-benzofuranyl)-2,2-dimethyl-5-chromenol | Retinoic acid receptor RXR-alpha | RXRA |  |
| 8-prenylated eriodictyol | Estrogen receptor | ESR1 |  |
| 8-prenylated eriodictyol | Sodium channel protein type 5 subunit alpha | SCN5A |  |
| 8-prenylated eriodictyol | Prostaglandin G/H synthase 2 | PTGS2 |  |
| 8-prenylated eriodictyol | Coagulation factor VII | F7 |  |
| 8-prenylated eriodictyol | Nuclear receptor coactivator 1 | NCOA1 |  |
| berberine | Nitric oxide synthase, inducible | NOS2 |  |
| berberine | Prostaglandin G/H synthase 1 | PTGS1 |  |
| berberine | Potassium voltage-gated channel subfamily H member 2 | KCNH2 |  |
| berberine | Estrogen receptor | ESR1 |  |
| berberine | Androgen receptor | AR |  |
| berberine | Sodium channel protein type 5 subunit alpha | SCN5A |  |
| berberine | Prostaglandin G/H synthase 2 | PTGS2 |  |
| berberine | Retinoic acid receptor RXR-alpha | RXRA |  |
| berberine | Beta-2 adrenergic receptor | ADRB2 |  |
| berberine | Trypsin-1 | PRSS1 |  |
| berberine | Nuclear receptor coactivator 2 | NCOA2 |  |
| berberine | cAMP and cAMP-inhibited cGMP 3',5'-cyclic phosphodiesterase 10A | PDE10A |  |
| beta-carotene | RAC-alpha serine/threonine-protein kinase | AKT1 |  |
| beta-carotene | Vascular endothelial growth factor A | VEGFA |  |
| beta-carotene | Apoptosis regulator Bcl-2 | BCL2 |  |
| beta-carotene | Caspase-9 | CASP9 |  |
| beta-carotene | 72 kDa type IV collagenase | MMP2 |  |
| beta-carotene | Transcription factor AP-1 | JUN |  |
| beta-carotene | Caspase-3 | CASP3 |  |
| beta-carotene | Prostaglandin G/H synthase 2 | PTGS2 |  |
| beta-carotene | Caspase-8 | CASP8 |  |
| beta-carotene | Interstitial collagenase | MMP1 |  |
| beta-carotene | Heme oxygenase 1 | HMOX1 |  |
| beta-carotene | Cytochrome P450 3A4 | CYP3A4 |  |
| beta-carotene | Cytochrome P450 1A2 | CYP1A2 |  |
| beta-carotene | Serum albumin | ALB |  |
| beta-carotene | Caveolin-1 | CAV1 |  |
| beta-carotene | Catenin beta-1 | CTNNB1 |  |
| beta-carotene | Myc proto-oncogene protein | MYC |  |
| beta-carotene | Caspase-7 | CASP7 |  |
| beta-carotene | Tissue factor | F3 |  |
| beta-carotene | Gap junction alpha-1 protein | GJA1 |  |
| beta-carotene | Stromelysin-2 | MMP10 |  |
| beta-sitosterol | Progesterone receptor | PGR |  |
| beta-sitosterol | Nuclear receptor coactivator 2 | NCOA2 |  |
| beta-sitosterol | Prostaglandin G/H synthase 1 | PTGS1 |  |
| beta-sitosterol | Prostaglandin G/H synthase 2 | PTGS2 |  |
| beta-sitosterol | Potassium voltage-gated channel subfamily H member 2 | KCNH2 |  |
| beta-sitosterol | Muscarinic acetylcholine receptor M3 | CHRM3 |  |
| beta-sitosterol | Muscarinic acetylcholine receptor M1 | CHRM1 |  |
| beta-sitosterol | Sodium channel protein type 5 subunit alpha | SCN5A |  |
| beta-sitosterol | Muscarinic acetylcholine receptor M4 | CHRM4 |  |
| beta-sitosterol | Alpha-1A adrenergic receptor | ADRA1A |  |
| beta-sitosterol | Muscarinic acetylcholine receptor M2 | CHRM2 |  |
| beta-sitosterol | Alpha-1B adrenergic receptor | ADRA1B |  |
| beta-sitosterol | Beta-2 adrenergic receptor | ADRB2 |  |
| beta-sitosterol | Neuronal acetylcholine receptor subunit alpha-2 | CHRNA2 |  |
| beta-sitosterol | Sodium-dependent serotonin transporter | SLC6A4 |  |
| beta-sitosterol | Mu-type opioid receptor | OPRM1 |  |
| beta-sitosterol | Gamma-aminobutyric acid receptor subunit alpha-1 | GABRA1 |  |
| beta-sitosterol | Apoptosis regulator Bcl-2 | BCL2 |  |
| beta-sitosterol | Apoptosis regulator BAX | BAX |  |
| beta-sitosterol | Caspase-9 | CASP9 |  |
| beta-sitosterol | Transcription factor AP-1 | JUN |  |
| beta-sitosterol | Caspase-3 | CASP3 |  |
| beta-sitosterol | Caspase-8 | CASP8 |  |
| beta-sitosterol | Protein kinase C alpha type | PRKCA |  |
| beta-sitosterol | Serum paraoxonase/arylesterase 1 | PON1 |  |
| beta-sitosterol | Microtubule-associated protein 2 | MAP2 |  |
| beta-sitosterol | Progesterone receptor | PGR |  |
| beta-sitosterol | Nuclear receptor coactivator 2 | NCOA2 |  |
| beta-sitosterol | Prostaglandin G/H synthase 1 | PTGS1 |  |
| beta-sitosterol | Prostaglandin G/H synthase 2 | PTGS2 |  |
| beta-sitosterol | Potassium voltage-gated channel subfamily H member 2 | KCNH2 |  |
| beta-sitosterol | Muscarinic acetylcholine receptor M3 | CHRM3 |  |
| beta-sitosterol | Muscarinic acetylcholine receptor M1 | CHRM1 |  |
| beta-sitosterol | Sodium channel protein type 5 subunit alpha | SCN5A |  |
| beta-sitosterol | Muscarinic acetylcholine receptor M4 | CHRM4 |  |
| beta-sitosterol | Alpha-1A adrenergic receptor | ADRA1A |  |
| beta-sitosterol | Muscarinic acetylcholine receptor M2 | CHRM2 |  |
| beta-sitosterol | Alpha-1B adrenergic receptor | ADRA1B |  |
| beta-sitosterol | Beta-2 adrenergic receptor | ADRB2 |  |
| beta-sitosterol | Neuronal acetylcholine receptor subunit alpha-2 | CHRNA2 |  |
| beta-sitosterol | Sodium-dependent serotonin transporter | SLC6A4 |  |
| beta-sitosterol | Mu-type opioid receptor | OPRM1 |  |
| beta-sitosterol | Gamma-aminobutyric acid receptor subunit alpha-1 | GABRA1 |  |
| beta-sitosterol | Apoptosis regulator Bcl-2 | BCL2 |  |
| beta-sitosterol | Apoptosis regulator BAX | BAX |  |
| beta-sitosterol | Caspase-9 | CASP9 |  |
| beta-sitosterol | Transcription factor AP-1 | JUN |  |
| beta-sitosterol | Caspase-3 | CASP3 |  |
| beta-sitosterol | Caspase-8 | CASP8 |  |
| beta-sitosterol | Protein kinase C alpha type | PRKCA |  |
| beta-sitosterol | Serum paraoxonase/arylesterase 1 | PON1 |  |
| beta-sitosterol | Microtubule-associated protein 2 | MAP2 |  |
| beta-sitosterol | Progesterone receptor | PGR |  |
| beta-sitosterol | Nuclear receptor coactivator 2 | NCOA2 |  |
| beta-sitosterol | Prostaglandin G/H synthase 1 | PTGS1 |  |
| beta-sitosterol | Prostaglandin G/H synthase 2 | PTGS2 |  |
| beta-sitosterol | Potassium voltage-gated channel subfamily H member 2 | KCNH2 |  |
| beta-sitosterol | Muscarinic acetylcholine receptor M3 | CHRM3 |  |
| beta-sitosterol | Muscarinic acetylcholine receptor M1 | CHRM1 |  |
| beta-sitosterol | Sodium channel protein type 5 subunit alpha | SCN5A |  |
| beta-sitosterol | Muscarinic acetylcholine receptor M4 | CHRM4 |  |
| beta-sitosterol | Alpha-1A adrenergic receptor | ADRA1A |  |
| beta-sitosterol | Muscarinic acetylcholine receptor M2 | CHRM2 |  |
| beta-sitosterol | Alpha-1B adrenergic receptor | ADRA1B |  |
| beta-sitosterol | Beta-2 adrenergic receptor | ADRB2 |  |
| beta-sitosterol | Neuronal acetylcholine receptor subunit alpha-2 | CHRNA2 |  |
| beta-sitosterol | Sodium-dependent serotonin transporter | SLC6A4 |  |
| beta-sitosterol | Mu-type opioid receptor | OPRM1 |  |
| beta-sitosterol | Gamma-aminobutyric acid receptor subunit alpha-1 | GABRA1 |  |
| beta-sitosterol | Apoptosis regulator Bcl-2 | BCL2 |  |
| beta-sitosterol | Apoptosis regulator BAX | BAX |  |
| beta-sitosterol | Caspase-9 | CASP9 |  |
| beta-sitosterol | Transcription factor AP-1 | JUN |  |
| beta-sitosterol | Caspase-3 | CASP3 |  |
| beta-sitosterol | Caspase-8 | CASP8 |  |
| beta-sitosterol | Protein kinase C alpha type | PRKCA |  |
| beta-sitosterol | Serum paraoxonase/arylesterase 1 | PON1 |  |
| beta-sitosterol | Microtubule-associated protein 2 | MAP2 |  |
| beta-sitosterol | Progesterone receptor | PGR |  |
| beta-sitosterol | Nuclear receptor coactivator 2 | NCOA2 |  |
| beta-sitosterol | Prostaglandin G/H synthase 1 | PTGS1 |  |
| beta-sitosterol | Prostaglandin G/H synthase 2 | PTGS2 |  |
| beta-sitosterol | Potassium voltage-gated channel subfamily H member 2 | KCNH2 |  |
| beta-sitosterol | Muscarinic acetylcholine receptor M3 | CHRM3 |  |
| beta-sitosterol | Muscarinic acetylcholine receptor M1 | CHRM1 |  |
| beta-sitosterol | Sodium channel protein type 5 subunit alpha | SCN5A |  |
| beta-sitosterol | Muscarinic acetylcholine receptor M4 | CHRM4 |  |
| beta-sitosterol | Alpha-1A adrenergic receptor | ADRA1A |  |
| beta-sitosterol | Muscarinic acetylcholine receptor M2 | CHRM2 |  |
| beta-sitosterol | Alpha-1B adrenergic receptor | ADRA1B |  |
| beta-sitosterol | Beta-2 adrenergic receptor | ADRB2 |  |
| beta-sitosterol | Neuronal acetylcholine receptor subunit alpha-2 | CHRNA2 |  |
| beta-sitosterol | Sodium-dependent serotonin transporter | SLC6A4 |  |
| beta-sitosterol | Mu-type opioid receptor | OPRM1 |  |
| beta-sitosterol | Gamma-aminobutyric acid receptor subunit alpha-1 | GABRA1 |  |
| beta-sitosterol | Apoptosis regulator Bcl-2 | BCL2 |  |
| beta-sitosterol | Apoptosis regulator BAX | BAX |  |
| beta-sitosterol | Caspase-9 | CASP9 |  |
| beta-sitosterol | Transcription factor AP-1 | JUN |  |
| beta-sitosterol | Caspase-3 | CASP3 |  |
| beta-sitosterol | Caspase-8 | CASP8 |  |
| beta-sitosterol | Protein kinase C alpha type | PRKCA |  |
| beta-sitosterol | Serum paraoxonase/arylesterase 1 | PON1 |  |
| beta-sitosterol | Microtubule-associated protein 2 | MAP2 |  |
| Calycosin | Nitric oxide synthase, inducible | NOS2 |  |
| Calycosin | Prostaglandin G/H synthase 1 | PTGS1 |  |
| Calycosin | Estrogen receptor | ESR1 |  |
| Calycosin | Androgen receptor | AR |  |
| Calycosin | Peroxisome proliferator activated receptor gamma | PPARG |  |
| Calycosin | Prostaglandin G/H synthase 2 | PTGS2 |  |
| Calycosin | Retinoic acid receptor RXR-alpha | RXRA |  |
| Calycosin | Estrogen receptor beta | ESR2 |  |
| Calycosin | Mitogen-activated protein kinase 14 | MAPK14 |  |
| Calycosin | Glycogen synthase kinase-3 beta | GSK3B |  |
| Calycosin | Serine/threonine-protein kinase Chk1 | CHEK1 |  |
| Calycosin | Trypsin-1 | PRSS1 |  |
| Calycosin | Cyclin-A2 | CCNA2 |  |
| Calycosin | Nuclear receptor coactivator 2 | NCOA2 |  |
| Calycosin | Beta-2 adrenergic receptor | ADRB2 |  |
| coumestrol | Peroxisome proliferator activated receptor gamma | PPARG |  |
| coumestrol | Prostaglandin G/H synthase 2 | PTGS2 |  |
| coumestrol | Cytochrome P450 3A4 | CYP3A4 |  |
| coumestrol | Nuclear receptor subfamily 1 group I member 2 | NR1I2 |  |
| coumestrol | Cytochrome P450 2B6 | CYP2B6 |  |
| coumestrol | Hsp90 co-chaperone Cdc37 | CDC37 |  |
| dehydroglyasperins C | Nitric oxide synthase, inducible | NOS2 |  |
| dehydroglyasperins C | Estrogen receptor | ESR1 |  |
| dehydroglyasperins C | Androgen receptor | AR |  |
| dehydroglyasperins C | Sodium channel protein type 5 subunit alpha | SCN5A |  |
| dehydroglyasperins C | Peroxisome proliferator activated receptor gamma | PPARG |  |
| dehydroglyasperins C | Prostaglandin G/H synthase 2 | PTGS2 |  |
| dehydroglyasperins C | Beta-2 adrenergic receptor | ADRB2 |  |
| dehydroglyasperins C | Estrogen receptor beta | ESR2 |  |
| dehydroglyasperins C | Mitogen-activated protein kinase 14 | MAPK14 |  |
| dehydroglyasperins C | Serine/threonine-protein kinase Chk1 | CHEK1 |  |
| dehydroglyasperins C | Trypsin-1 | PRSS1 |  |
| dehydroglyasperins C | Cyclin-A2 | CCNA2 |  |
| dehydroglyasperins C | Nuclear receptor coactivator 2 | NCOA2 |  |
| DFV | Prostaglandin G/H synthase 1 | PTGS1 |  |
| DFV | Estrogen receptor | ESR1 |  |
| DFV | Prostaglandin G/H synthase 2 | PTGS2 |  |
| DFV | Retinoic acid receptor RXR-alpha | RXRA |  |
| DFV | Beta-2 adrenergic receptor | ADRB2 |  |
| DFV | Amine oxidase [flavin-containing] B | MAOB |  |
| DFV | Sodium-dependent serotonin transporter | SLC6A4 |  |
| DFV | cAMP-dependent protein kinase inhibitor alpha | PKIA |  |
| ent-Epicatechin | Prostaglandin G/H synthase 1 | PTGS1 |  |
| ent-Epicatechin | Estrogen receptor | ESR1 |  |
| ent-Epicatechin | Prostaglandin G/H synthase 2 | PTGS2 |  |
| euchrenone | Nitric oxide synthase, inducible | NOS2 |  |
| euchrenone | Potassium voltage-gated channel subfamily H member 2 | KCNH2 |  |
| euchrenone | Estrogen receptor | ESR1 |  |
| euchrenone | Sodium channel protein type 5 subunit alpha | SCN5A |  |
| euchrenone | Prostaglandin G/H synthase 2 | PTGS2 |  |
| euchrenone | Estrogen receptor beta | ESR2 |  |
| Eurycarpin A | Nitric oxide synthase, inducible | NOS2 |  |
| Eurycarpin A | Estrogen receptor | ESR1 |  |
| Eurycarpin A | Androgen receptor | AR |  |
| Eurycarpin A | Sodium channel protein type 5 subunit alpha | SCN5A |  |
| Eurycarpin A | Peroxisome proliferator activated receptor gamma | PPARG |  |
| Eurycarpin A | Prostaglandin G/H synthase 2 | PTGS2 |  |
| Eurycarpin A | Estrogen receptor beta | ESR2 |  |
| Eurycarpin A | Mitogen-activated protein kinase 14 | MAPK14 |  |
| Eurycarpin A | Glycogen synthase kinase-3 beta | GSK3B |  |
| Eurycarpin A | Serine/threonine-protein kinase Chk1 | CHEK1 |  |
| Eurycarpin A | Trypsin-1 | PRSS1 |  |
| Eurycarpin A | Cyclin-A2 | CCNA2 |  |
| formononetin | Nitric oxide synthase, inducible | NOS2 |  |
| formononetin | Prostaglandin G/H synthase 1 | PTGS1 |  |
| formononetin | Muscarinic acetylcholine receptor M1 | CHRM1 |  |
| formononetin | Estrogen receptor | ESR1 |  |
| formononetin | Androgen receptor | AR |  |
| formononetin | Peroxisome proliferator activated receptor gamma | PPARG |  |
| formononetin | Prostaglandin G/H synthase 2 | PTGS2 |  |
| formononetin | Retinoic acid receptor RXR-alpha | RXRA |  |
| formononetin | Alpha-1A adrenergic receptor | ADRA1A |  |
| formononetin | Sodium-dependent dopamine transporter | SLC6A3 |  |
| formononetin | Beta-2 adrenergic receptor | ADRB2 |  |
| formononetin | Sodium-dependent serotonin transporter | SLC6A4 |  |
| formononetin | Estrogen receptor beta | ESR2 |  |
| formononetin | Mitogen-activated protein kinase 14 | MAPK14 |  |
| formononetin | Glycogen synthase kinase-3 beta | GSK3B |  |
| formononetin | Amine oxidase [flavin-containing] B | MAOB |  |
| formononetin | Serine/threonine-protein kinase Chk1 | CHEK1 |  |
| formononetin | Trypsin-1 | PRSS1 |  |
| formononetin | Cyclin-A2 | CCNA2 |  |
| formononetin | cAMP-dependent protein kinase inhibitor alpha | PKIA |  |
| formononetin | Acetylcholinesterase | ACHE |  |
| formononetin | Transcription factor AP-1 | JUN |  |
| formononetin | Peroxisome proliferator-activated receptor gamma | PPARG |  |
| formononetin | Interleukin-4 | IL4 |  |
| formononetin | ATP synthase subunit beta, mitochondrial | ATP5F1B |  |
| formononetin | NADH-ubiquinone oxidoreductase chain 6 | ND6 |  |
| formononetin | 3 beta-hydroxysteroid dehydrogenase/Delta 5-->4-isomerase type 2 | HSD3B2 |  |
| formononetin | 3 beta-hydroxysteroid dehydrogenase/Delta 5-->4-isomerase type 1 | HSD3B1 |  |
| Fumarine | Prostaglandin G/H synthase 1 | PTGS1 |  |
| Fumarine | Muscarinic acetylcholine receptor M3 | CHRM3 |  |
| Fumarine | Potassium voltage-gated channel subfamily H member 2 | KCNH2 |  |
| Fumarine | Muscarinic acetylcholine receptor M1 | CHRM1 |  |
| Fumarine | Sodium channel protein type 5 subunit alpha | SCN5A |  |
| Fumarine | Muscarinic acetylcholine receptor M5 | CHRM5 |  |
| Fumarine | Prostaglandin G/H synthase 2 | PTGS2 |  |
| Fumarine | 5-hydroxytryptamine receptor 3A | HTR3A |  |
| Fumarine | Coagulation factor VII | F7 |  |
| Fumarine | Muscarinic acetylcholine receptor M4 | CHRM4 |  |
| Fumarine | Delta-type opioid receptor | OPRD1 |  |
| Fumarine | Alpha-1B adrenergic receptor | ADRA1B |  |
| Fumarine | Beta-2 adrenergic receptor | ADRB2 |  |
| Fumarine | Alpha-1D adrenergic receptor | ADRA1D |  |
| Fumarine | Mu-type opioid receptor | OPRM1 |  |
| Fumarine | Sodium-dependent serotonin transporter | SLC6A4 |  |
| Fumarine | Voltage-dependent L-type calcium channel subunit alpha-1S | CACNA1S |  |
| Fumarine | Sodium-dependent dopamine transporter | SLC6A3 |  |
| Fumarine | Vascular endothelial growth factor receptor 2 | KDR |  |
| gadelaidic acid | Nuclear receptor coactivator 2 | NCOA2 |  |
| Gancaonin A | Nitric oxide synthase, inducible | NOS2 |  |
| Gancaonin A | Estrogen receptor | ESR1 |  |
| Gancaonin A | Androgen receptor | AR |  |
| Gancaonin A | Sodium channel protein type 5 subunit alpha | SCN5A |  |
| Gancaonin A | Peroxisome proliferator activated receptor gamma | PPARG |  |
| Gancaonin A | Prostaglandin G/H synthase 2 | PTGS2 |  |
| Gancaonin A | Acetylcholinesterase | ACHE |  |
| Gancaonin A | Estrogen receptor beta | ESR2 |  |
| Gancaonin A | Glycogen synthase kinase-3 beta | GSK3B |  |
| Gancaonin A | Serine/threonine-protein kinase Chk1 | CHEK1 |  |
| Gancaonin A | Trypsin-1 | PRSS1 |  |
| Gancaonin A | Cyclin-A2 | CCNA2 |  |
| Gancaonin A | Nuclear receptor coactivator 2 | NCOA2 |  |
| Gancaonin B | Nitric oxide synthase, inducible | NOS2 |  |
| Gancaonin B | Estrogen receptor | ESR1 |  |
| Gancaonin B | Androgen receptor | AR |  |
| Gancaonin B | Peroxisome proliferator activated receptor gamma | PPARG |  |
| Gancaonin B | Prostaglandin G/H synthase 2 | PTGS2 |  |
| Gancaonin B | Coagulation factor VII | F7 |  |
| Gancaonin B | Vascular endothelial growth factor receptor 2 | KDR |  |
| Gancaonin B | Alpha-1B adrenergic receptor | ADRA1B |  |
| Gancaonin B | Beta-2 adrenergic receptor | ADRB2 |  |
| Gancaonin B | Estrogen receptor beta | ESR2 |  |
| Gancaonin B | Glycogen synthase kinase-3 beta | GSK3B |  |
| Gancaonin B | Serine/threonine-protein kinase Chk1 | CHEK1 |  |
| Gancaonin B | Trypsin-1 | PRSS1 |  |
| Gancaonin B | Cyclin-A2 | CCNA2 |  |
| Gancaonin B | Nuclear receptor coactivator 2 | NCOA2 |  |
| Gancaonin G | Nitric oxide synthase, inducible | NOS2 |  |
| Gancaonin G | Estrogen receptor | ESR1 |  |
| Gancaonin G | Androgen receptor | AR |  |
| Gancaonin G | Peroxisome proliferator activated receptor gamma | PPARG |  |
| Gancaonin G | Prostaglandin G/H synthase 2 | PTGS2 |  |
| Gancaonin G | Estrogen receptor beta | ESR2 |  |
| Gancaonin G | Mitogen-activated protein kinase 14 | MAPK14 |  |
| Gancaonin G | Glycogen synthase kinase-3 beta | GSK3B |  |
| Gancaonin G | Serine/threonine-protein kinase Chk1 | CHEK1 |  |
| Gancaonin G | Trypsin-1 | PRSS1 |  |
| Gancaonin G | Cyclin-A2 | CCNA2 |  |
| Gancaonin G | Nuclear receptor coactivator 2 | NCOA2 |  |
| Gancaonin H | Estrogen receptor | ESR1 |  |
| Gancaonin H | Androgen receptor | AR |  |
| Gancaonin H | Prostaglandin G/H synthase 2 | PTGS2 |  |
| Gancaonin H | Vascular endothelial growth factor receptor 2 | KDR |  |
| Gancaonin H | Trypsin-1 | PRSS1 |  |
| Gancaonin H | Cyclin-A2 | CCNA2 |  |
| Gancaonin H | Nuclear receptor coactivator 2 | NCOA2 |  |
| Glabranin | Nitric oxide synthase, inducible | NOS2 |  |
| Glabranin | Prostaglandin G/H synthase 1 | PTGS1 |  |
| Glabranin | Estrogen receptor | ESR1 |  |
| Glabranin | Sodium channel protein type 5 subunit alpha | SCN5A |  |
| Glabranin | Prostaglandin G/H synthase 2 | PTGS2 |  |
| Glabrene | Nitric oxide synthase, inducible | NOS2 |  |
| Glabrene | Prostaglandin G/H synthase 1 | PTGS1 |  |
| Glabrene | Estrogen receptor | ESR1 |  |
| Glabrene | Androgen receptor | AR |  |
| Glabrene | Sodium channel protein type 5 subunit alpha | SCN5A |  |
| Glabrene | Peroxisome proliferator activated receptor gamma | PPARG |  |
| Glabrene | Prostaglandin G/H synthase 2 | PTGS2 |  |
| Glabrene | Retinoic acid receptor RXR-alpha | RXRA |  |
| Glabrene | Beta-2 adrenergic receptor | ADRB2 |  |
| Glabrene | Estrogen receptor beta | ESR2 |  |
| Glabrene | Mitogen-activated protein kinase 14 | MAPK14 |  |
| Glabrene | Glycogen synthase kinase-3 beta | GSK3B |  |
| Glabrene | Trypsin-1 | PRSS1 |  |
| Glabrene | Nuclear receptor coactivator 2 | NCOA2 |  |
| Glabridin | Nitric oxide synthase, inducible | NOS2 |  |
| Glabridin | Muscarinic acetylcholine receptor M1 | CHRM1 |  |
| Glabridin | Estrogen receptor | ESR1 |  |
| Glabridin | Androgen receptor | AR |  |
| Glabridin | Sodium channel protein type 5 subunit alpha | SCN5A |  |
| Glabridin | Peroxisome proliferator activated receptor gamma | PPARG |  |
| Glabridin | Prostaglandin G/H synthase 2 | PTGS2 |  |
| Glabridin | Retinoic acid receptor RXR-alpha | RXRA |  |
| Glabridin | Acetylcholinesterase | ACHE |  |
| Glabridin | Alpha-1B adrenergic receptor | ADRA1B |  |
| Glabridin | Beta-2 adrenergic receptor | ADRB2 |  |
| Glabridin | Estrogen receptor beta | ESR2 |  |
| Glabridin | Mitogen-activated protein kinase 14 | MAPK14 |  |
| Glabridin | Glycogen synthase kinase-3 beta | GSK3B |  |
| Glabridin | Serine/threonine-protein kinase Chk1 | CHEK1 |  |
| Glabridin | Retinoic acid receptor RXR-beta | RXRB |  |
| Glabridin | Trypsin-1 | PRSS1 |  |
| Glabridin | Cyclin-A2 | CCNA2 |  |
| Glabridin | Nuclear receptor coactivator 2 | NCOA2 |  |
| Glabridin | Nuclear receptor coactivator 1 | NCOA1 |  |
| Glabrone | Nitric oxide synthase, inducible | NOS2 |  |
| Glabrone | Prostaglandin G/H synthase 1 | PTGS1 |  |
| Glabrone | Estrogen receptor | ESR1 |  |
| Glabrone | Androgen receptor | AR |  |
| Glabrone | Sodium channel protein type 5 subunit alpha | SCN5A |  |
| Glabrone | Peroxisome proliferator activated receptor gamma | PPARG |  |
| Glabrone | Prostaglandin G/H synthase 2 | PTGS2 |  |
| Glabrone | Retinoic acid receptor RXR-alpha | RXRA |  |
| Glabrone | Acetylcholinesterase | ACHE |  |
| Glabrone | Estrogen receptor beta | ESR2 |  |
| Glabrone | Mitogen-activated protein kinase 14 | MAPK14 |  |
| Glabrone | Glycogen synthase kinase-3 beta | GSK3B |  |
| Glabrone | Serine/threonine-protein kinase Chk1 | CHEK1 |  |
| Glabrone | Trypsin-1 | PRSS1 |  |
| Glabrone | Cyclin-A2 | CCNA2 |  |
| Glepidotin A | Nitric oxide synthase, inducible | NOS2 |  |
| Glepidotin A | Prostaglandin G/H synthase 1 | PTGS1 |  |
| Glepidotin A | Estrogen receptor | ESR1 |  |
| Glepidotin A | Androgen receptor | AR |  |
| Glepidotin A | Sodium channel protein type 5 subunit alpha | SCN5A |  |
| Glepidotin A | Peroxisome proliferator activated receptor gamma | PPARG |  |
| Glepidotin A | Prostaglandin G/H synthase 2 | PTGS2 |  |
| Glepidotin A | Coagulation factor VII | F7 |  |
| Glepidotin A | Vascular endothelial growth factor receptor 2 | KDR |  |
| Glepidotin A | Retinoic acid receptor RXR-alpha | RXRA |  |
| Glepidotin A | Mitogen-activated protein kinase 14 | MAPK14 |  |
| Glepidotin A | Glycogen synthase kinase-3 beta | GSK3B |  |
| Glepidotin A | Serine/threonine-protein kinase Chk1 | CHEK1 |  |
| Glepidotin A | Trypsin-1 | PRSS1 |  |
| Glepidotin A | Cyclin-A2 | CCNA2 |  |
| Glepidotin B | Prostaglandin G/H synthase 1 | PTGS1 |  |
| Glepidotin B | Estrogen receptor | ESR1 |  |
| Glepidotin B | Sodium channel protein type 5 subunit alpha | SCN5A |  |
| Glepidotin B | Prostaglandin G/H synthase 2 | PTGS2 |  |
| Glepidotin B | Coagulation factor VII | F7 |  |
| Glepidotin B | Retinoic acid receptor RXR-alpha | RXRA |  |
| Glepidotin B | Alpha-1B adrenergic receptor | ADRA1B |  |
| Glepidotin B | Nuclear receptor coactivator 1 | NCOA1 |  |
| glyasperin B | Nitric oxide synthase, inducible | NOS2 |  |
| glyasperin B | Estrogen receptor | ESR1 |  |
| glyasperin B | Androgen receptor | AR |  |
| glyasperin B | Peroxisome proliferator activated receptor gamma | PPARG |  |
| glyasperin B | Prostaglandin G/H synthase 2 | PTGS2 |  |
| glyasperin B | Coagulation factor VII | F7 |  |
| glyasperin B | Vascular endothelial growth factor receptor 2 | KDR |  |
| glyasperin B | Acetylcholinesterase | ACHE |  |
| glyasperin B | Estrogen receptor beta | ESR2 |  |
| glyasperin B | Glycogen synthase kinase-3 beta | GSK3B |  |
| glyasperin B | Trypsin-1 | PRSS1 |  |
| glyasperin B | Cyclin-A2 | CCNA2 |  |
| glyasperin B | Nuclear receptor coactivator 2 | NCOA2 |  |
| Glyasperin C | Nitric oxide synthase, inducible | NOS2 |  |
| Glyasperin C | Potassium voltage-gated channel subfamily H member 2 | KCNH2 |  |
| Glyasperin C | Estrogen receptor | ESR1 |  |
| Glyasperin C | Androgen receptor | AR |  |
| Glyasperin C | Sodium channel protein type 5 subunit alpha | SCN5A |  |
| Glyasperin C | Peroxisome proliferator activated receptor gamma | PPARG |  |
| Glyasperin C | Prostaglandin G/H synthase 2 | PTGS2 |  |
| Glyasperin C | Retinoic acid receptor RXR-alpha | RXRA |  |
| Glyasperin C | Acetylcholinesterase | ACHE |  |
| Glyasperin C | Estrogen receptor beta | ESR2 |  |
| Glyasperin C | Mitogen-activated protein kinase 14 | MAPK14 |  |
| Glyasperin C | Glycogen synthase kinase-3 beta | GSK3B |  |
| Glyasperin C | Serine/threonine-protein kinase Chk1 | CHEK1 |  |
| Glyasperin C | Trypsin-1 | PRSS1 |  |
| Glyasperin C | Cyclin-A2 | CCNA2 |  |
| Glyasperin C | Nuclear receptor coactivator 2 | NCOA2 |  |
| glyasperin F | Nitric oxide synthase, inducible | NOS2 |  |
| glyasperin F | Prostaglandin G/H synthase 1 | PTGS1 |  |
| glyasperin F | Estrogen receptor | ESR1 |  |
| glyasperin F | Androgen receptor | AR |  |
| glyasperin F | Sodium channel protein type 5 subunit alpha | SCN5A |  |
| glyasperin F | Peroxisome proliferator activated receptor gamma | PPARG |  |
| glyasperin F | Prostaglandin G/H synthase 2 | PTGS2 |  |
| glyasperin F | Estrogen receptor beta | ESR2 |  |
| glyasperin F | Mitogen-activated protein kinase 14 | MAPK14 |  |
| glyasperin F | Glycogen synthase kinase-3 beta | GSK3B |  |
| glyasperin F | Trypsin-1 | PRSS1 |  |
| glyasperin F | Cyclin-A2 | CCNA2 |  |
| Glyasperins M | Nitric oxide synthase, inducible | NOS2 |  |
| Glyasperins M | Prostaglandin G/H synthase 1 | PTGS1 |  |
| Glyasperins M | Potassium voltage-gated channel subfamily H member 2 | KCNH2 |  |
| Glyasperins M | Estrogen receptor | ESR1 |  |
| Glyasperins M | Androgen receptor | AR |  |
| Glyasperins M | Sodium channel protein type 5 subunit alpha | SCN5A |  |
| Glyasperins M | Peroxisome proliferator activated receptor gamma | PPARG |  |
| Glyasperins M | Prostaglandin G/H synthase 2 | PTGS2 |  |
| Glyasperins M | Coagulation factor VII | F7 |  |
| Glyasperins M | Vascular endothelial growth factor receptor 2 | KDR |  |
| Glyasperins M | Acetylcholinesterase | ACHE |  |
| Glyasperins M | Estrogen receptor beta | ESR2 |  |
| Glyasperins M | Glycogen synthase kinase-3 beta | GSK3B |  |
| Glyasperins M | Trypsin-1 | PRSS1 |  |
| Glyasperins M | Cyclin-A2 | CCNA2 |  |
| Glyasperins M | Nuclear receptor coactivator 2 | NCOA2 |  |
| Glyasperins M | Nuclear receptor coactivator 1 | NCOA1 |  |
| Glycyrin | Nitric oxide synthase, inducible | NOS2 |  |
| Glycyrin | Potassium voltage-gated channel subfamily H member 2 | KCNH2 |  |
| Glycyrin | Estrogen receptor | ESR1 |  |
| Glycyrin | Androgen receptor | AR |  |
| Glycyrin | Peroxisome proliferator activated receptor gamma | PPARG |  |
| Glycyrin | Prostaglandin G/H synthase 2 | PTGS2 |  |
| Glycyrin | Vascular endothelial growth factor receptor 2 | KDR |  |
| Glycyrin | Estrogen receptor beta | ESR2 |  |
| Glycyrin | Serine/threonine-protein kinase Chk1 | CHEK1 |  |
| Glycyrin | Trypsin-1 | PRSS1 |  |
| Glycyrin | Nuclear receptor coactivator 2 | NCOA2 |  |
| Glycyrol | Nitric oxide synthase, inducible | NOS2 |  |
| Glycyrol | Estrogen receptor | ESR1 |  |
| Glycyrol | Peroxisome proliferator activated receptor gamma | PPARG |  |
| Glycyrol | Prostaglandin G/H synthase 2 | PTGS2 |  |
| Glycyrol | Vascular endothelial growth factor receptor 2 | KDR |  |
| Glycyrol | Mitogen-activated protein kinase 14 | MAPK14 |  |
| Glycyrol | Glycogen synthase kinase-3 beta | GSK3B |  |
| Glycyrol | Serine/threonine-protein kinase Chk1 | CHEK1 |  |
| Glycyrol | Cyclin-A2 | CCNA2 |  |
| Glycyrrhiza flavonol A | Nitric oxide synthase, inducible | NOS2 |  |
| Glycyrrhiza flavonol A | Estrogen receptor | ESR1 |  |
| Glycyrrhiza flavonol A | Androgen receptor | AR |  |
| Glycyrrhiza flavonol A | Prostaglandin G/H synthase 2 | PTGS2 |  |
| Glycyrrhiza flavonol A | Coagulation factor VII | F7 |  |
| Glycyrrhiza flavonol A | Acetylcholinesterase | ACHE |  |
| Glycyrrhiza flavonol A | Estrogen receptor beta | ESR2 |  |
| Glycyrrhiza flavonol A | Glycogen synthase kinase-3 beta | GSK3B |  |
| Glycyrrhiza flavonol A | Trypsin-1 | PRSS1 |  |
| Glycyrrhiza flavonol A | Cyclin-A2 | CCNA2 |  |
| Glypallichalcone | Nitric oxide synthase, inducible | NOS2 |  |
| Glypallichalcone | Prostaglandin G/H synthase 1 | PTGS1 |  |
| Glypallichalcone | Muscarinic acetylcholine receptor M1 | CHRM1 |  |
| Glypallichalcone | Estrogen receptor | ESR1 |  |
| Glypallichalcone | Androgen receptor | AR |  |
| Glypallichalcone | Sodium channel protein type 5 subunit alpha | SCN5A |  |
| Glypallichalcone | Peroxisome proliferator activated receptor gamma | PPARG |  |
| Glypallichalcone | Prostaglandin G/H synthase 2 | PTGS2 |  |
| Glypallichalcone | Alpha-1B adrenergic receptor | ADRA1B |  |
| Glypallichalcone | Sodium-dependent dopamine transporter | SLC6A3 |  |
| Glypallichalcone | Beta-2 adrenergic receptor | ADRB2 |  |
| Glypallichalcone | Sodium-dependent serotonin transporter | SLC6A4 |  |
| Glypallichalcone | Estrogen receptor beta | ESR2 |  |
| Glypallichalcone | Mitogen-activated protein kinase 14 | MAPK14 |  |
| Glypallichalcone | Glycogen synthase kinase-3 beta | GSK3B |  |
| Glypallichalcone | Leukotriene A-4 hydrolase | LTA4H |  |
| Glypallichalcone | Amine oxidase [flavin-containing] B | MAOB |  |
| Glypallichalcone | Serine/threonine-protein kinase Chk1 | CHEK1 |  |
| Glypallichalcone | Cyclin-A2 | CCNA2 |  |
| Glypallichalcone | Nuclear receptor coactivator 1 | NCOA1 |  |
| Glypallichalcone | cAMP-dependent protein kinase inhibitor alpha | PKIA |  |
| Glyzaglabrin | Nitric oxide synthase, inducible | NOS2 |  |
| Glyzaglabrin | Prostaglandin G/H synthase 1 | PTGS1 |  |
| Glyzaglabrin | Estrogen receptor | ESR1 |  |
| Glyzaglabrin | Androgen receptor | AR |  |
| Glyzaglabrin | Peroxisome proliferator activated receptor gamma | PPARG |  |
| Glyzaglabrin | Prostaglandin G/H synthase 2 | PTGS2 |  |
| Glyzaglabrin | Estrogen receptor beta | ESR2 |  |
| Glyzaglabrin | Mitogen-activated protein kinase 14 | MAPK14 |  |
| Glyzaglabrin | Glycogen synthase kinase-3 beta | GSK3B |  |
| Glyzaglabrin | Serine/threonine-protein kinase Chk1 | CHEK1 |  |
| Glyzaglabrin | Trypsin-1 | PRSS1 |  |
| Glyzaglabrin | Cyclin-A2 | CCNA2 |  |
| HMO | Nitric oxide synthase, inducible | NOS2 |  |
| HMO | Prostaglandin G/H synthase 1 | PTGS1 |  |
| HMO | Muscarinic acetylcholine receptor M1 | CHRM1 |  |
| HMO | Estrogen receptor | ESR1 |  |
| HMO | Androgen receptor | AR |  |
| HMO | Sodium channel protein type 5 subunit alpha | SCN5A |  |
| HMO | Peroxisome proliferator activated receptor gamma | PPARG |  |
| HMO | Prostaglandin G/H synthase 2 | PTGS2 |  |
| HMO | Retinoic acid receptor RXR-alpha | RXRA |  |
| HMO | Sodium-dependent dopamine transporter | SLC6A3 |  |
| HMO | Beta-2 adrenergic receptor | ADRB2 |  |
| HMO | Sodium-dependent serotonin transporter | SLC6A4 |  |
| HMO | Estrogen receptor beta | ESR2 |  |
| HMO | Mitogen-activated protein kinase 14 | MAPK14 |  |
| HMO | Glycogen synthase kinase-3 beta | GSK3B |  |
| HMO | Amine oxidase [flavin-containing] B | MAOB |  |
| HMO | Serine/threonine-protein kinase Chk1 | CHEK1 |  |
| HMO | Trypsin-1 | PRSS1 |  |
| HMO | Cyclin-A2 | CCNA2 |  |
| HMO | cAMP-dependent protein kinase inhibitor alpha | PKIA |  |
| icos-5-enoic acid | Nuclear receptor coactivator 2 | NCOA2 |  |
| Inermine | Prostaglandin G/H synthase 1 | PTGS1 |  |
| Inermine | Muscarinic acetylcholine receptor M3 | CHRM3 |  |
| Inermine | Sodium channel protein type 5 subunit alpha | SCN5A |  |
| Inermine | Prostaglandin G/H synthase 2 | PTGS2 |  |
| Inermine | 5-hydroxytryptamine receptor 3A | HTR3A |  |
| Inermine | Retinoic acid receptor RXR-alpha | RXRA |  |
| Inermine | Alpha-1B adrenergic receptor | ADRA1B |  |
| Inermine | Alpha-1D adrenergic receptor | ADRA1D |  |
| Inermine | Trypsin-1 | PRSS1 |  |
| Inermine | Muscarinic acetylcholine receptor M1 | CHRM1 |  |
| Inermine | Beta-2 adrenergic receptor | ADRB2 |  |
| Inermine | Mu-type opioid receptor | OPRM1 |  |
| Inflacoumarin A | Estrogen receptor | ESR1 |  |
| Inflacoumarin A | Androgen receptor | AR |  |
| Inflacoumarin A | Peroxisome proliferator activated receptor gamma | PPARG |  |
| Inflacoumarin A | Prostaglandin G/H synthase 2 | PTGS2 |  |
| Inflacoumarin A | Beta-2 adrenergic receptor | ADRB2 |  |
| Inflacoumarin A | Trypsin-1 | PRSS1 |  |
| Inflacoumarin A | Nuclear receptor coactivator 2 | NCOA2 |  |
| Inflacoumarin A | Prostaglandin G/H synthase 1 | PTGS1 |  |
| Inflacoumarin A | Sodium channel protein type 5 subunit alpha | SCN5A |  |
| Isoglycyrol | Nitric oxide synthase, inducible | NOS2 |  |
| Isoglycyrol | Estrogen receptor | ESR1 |  |
| Isoglycyrol | Androgen receptor | AR |  |
| Isoglycyrol | Prostaglandin G/H synthase 2 | PTGS2 |  |
| Isoglycyrol | Glycogen synthase kinase-3 beta | GSK3B |  |
| Isolicoflavonol | Nitric oxide synthase, inducible | NOS2 |  |
| Isolicoflavonol | Estrogen receptor | ESR1 |  |
| Isolicoflavonol | Androgen receptor | AR |  |
| Isolicoflavonol | Peroxisome proliferator activated receptor gamma | PPARG |  |
| Isolicoflavonol | Prostaglandin G/H synthase 2 | PTGS2 |  |
| Isolicoflavonol | Glycogen synthase kinase-3 beta | GSK3B |  |
| Isolicoflavonol | Trypsin-1 | PRSS1 |  |
| Isolicoflavonol | Cyclin-A2 | CCNA2 |  |
| Isolicoflavonol | Nuclear receptor coactivator 2 | NCOA2 |  |
| isorhamnetin | Nitric oxide synthase, inducible | NOS2 |  |
| isorhamnetin | Prostaglandin G/H synthase 1 | PTGS1 |  |
| isorhamnetin | Estrogen receptor | ESR1 |  |
| isorhamnetin | Androgen receptor | AR |  |
| isorhamnetin | Peroxisome proliferator activated receptor gamma | PPARG |  |
| isorhamnetin | Prostaglandin G/H synthase 2 | PTGS2 |  |
| isorhamnetin | Estrogen receptor beta | ESR2 |  |
| isorhamnetin | Mitogen-activated protein kinase 14 | MAPK14 |  |
| isorhamnetin | Glycogen synthase kinase-3 beta | GSK3B |  |
| isorhamnetin | Trypsin-1 | PRSS1 |  |
| isorhamnetin | Cyclin-A2 | CCNA2 |  |
| isorhamnetin | Nuclear receptor coactivator 2 | NCOA2 |  |
| isorhamnetin | Glycogen phosphorylase, muscle form | PYGM |  |
| isorhamnetin | Serine/threonine-protein kinase Chk1 | CHEK1 |  |
| isorhamnetin | Aldose reductase | AKR1B1 |  |
| isorhamnetin | Nuclear receptor coactivator 1 | NCOA1 |  |
| isorhamnetin | Coagulation factor VII | F7 |  |
| isorhamnetin | Acetylcholinesterase | ACHE |  |
| isorhamnetin | Gamma-aminobutyric acid receptor subunit alpha-1 | GABRA1 |  |
| isorhamnetin | Amine oxidase [flavin-containing] B | MAOB |  |
| isorhamnetin | Glutamate receptor 2 | GRIA2 |  |
| isorhamnetin | Transcription factor p65 | RELA |  |
| isorhamnetin | Neutrophil cytosol factor 1 | NCF1 |  |
| isorhamnetin | Oxidized low-density lipoprotein receptor 1 | OLR1 |  |
| Isotrifoliol | Nitric oxide synthase, inducible | NOS2 |  |
| Isotrifoliol | Estrogen receptor | ESR1 |  |
| Isotrifoliol | Androgen receptor | AR |  |
| Isotrifoliol | Prostaglandin G/H synthase 2 | PTGS2 |  |
| Isotrifoliol | Estrogen receptor beta | ESR2 |  |
| Isotrifoliol | Mitogen-activated protein kinase 14 | MAPK14 |  |
| Isotrifoliol | Glycogen synthase kinase-3 beta | GSK3B |  |
| Isotrifoliol | Serine/threonine-protein kinase Chk1 | CHEK1 |  |
| Isotrifoliol | Cyclin-A2 | CCNA2 |  |
| Jaranol | Nitric oxide synthase, inducible | NOS2 |  |
| Jaranol | Prostaglandin G/H synthase 1 | PTGS1 |  |
| Jaranol | Androgen receptor | AR |  |
| Jaranol | Sodium channel protein type 5 subunit alpha | SCN5A |  |
| Jaranol | Prostaglandin G/H synthase 2 | PTGS2 |  |
| Jaranol | Estrogen receptor beta | ESR2 |  |
| Jaranol | Serine/threonine-protein kinase Chk1 | CHEK1 |  |
| Jaranol | Trypsin-1 | PRSS1 |  |
| Jaranol | Nuclear receptor coactivator 2 | NCOA2 |  |
| Jujubasaponin V_qt | Mineralocorticoid receptor | NR3C2 |  |
| kaempferol | Nitric oxide synthase, inducible | NOS2 |  |
| kaempferol | Prostaglandin G/H synthase 1 | PTGS1 |  |
| kaempferol | Androgen receptor | AR |  |
| kaempferol | Peroxisome proliferator activated receptor gamma | PPARG |  |
| kaempferol | Prostaglandin G/H synthase 2 | PTGS2 |  |
| kaempferol | Nuclear receptor coactivator 2 | NCOA2 |  |
| kaempferol | Trypsin-1 | PRSS1 |  |
| kaempferol | Progesterone receptor | PGR |  |
| kaempferol | Muscarinic acetylcholine receptor M1 | CHRM1 |  |
| kaempferol | Acetylcholinesterase | ACHE |  |
| kaempferol | Sodium-dependent noradrenaline transporter | SLC6A2 |  |
| kaempferol | Muscarinic acetylcholine receptor M2 | CHRM2 |  |
| kaempferol | Alpha-1B adrenergic receptor | ADRA1B |  |
| kaempferol | Gamma-aminobutyric acid receptor subunit alpha-1 | GABRA1 |  |
| kaempferol | Coagulation factor VII | F7 |  |
| kaempferol | Transcription factor p65 | RELA |  |
| kaempferol | Inhibitor of nuclear factor kappa-B kinase subunit beta | IKBKB |  |
| kaempferol | RAC-alpha serine/threonine-protein kinase | AKT1 |  |
| kaempferol | Apoptosis regulator Bcl-2 | BCL2 |  |
| kaempferol | Apoptosis regulator BAX | BAX |  |
| kaempferol | Tumor necrosis factor | TNFSF15 |  |
| kaempferol | Transcription factor AP-1 | JUN |  |
| kaempferol | Activator of 90 kDa heat shock protein ATPase homolog 1 | AHSA1 |  |
| kaempferol | Caspase-3 | CASP3 |  |
| kaempferol | Mitogen-activated protein kinase 8 | MAPK8 |  |
| kaempferol | Interstitial collagenase | MMP1 |  |
| kaempferol | Signal transducer and activator of transcription 1-alpha/beta | STAT1 |  |
| kaempferol | Peroxisome proliferator-activated receptor gamma | PPARG |  |
| kaempferol | Heme oxygenase 1 | HMOX1 |  |
| kaempferol | Cytochrome P450 3A4 | CYP3A4 |  |
| kaempferol | Cytochrome P450 1A2 | CYP1A2 |  |
| kaempferol | Cytochrome P450 1A1 | CYP1A1 |  |
| kaempferol | Intercellular adhesion molecule 1 | ICAM1 |  |
| kaempferol | E-selectin | SELE |  |
| kaempferol | Vascular cell adhesion protein 1 | VCAM1 |  |
| kaempferol | Nuclear receptor subfamily 1 group I member 2 | NR1I2 |  |
| kaempferol | Cytochrome P450 1B1 | CYP1B1 |  |
| kaempferol | Arachidonate 5-lipoxygenase | ALOX5 |  |
| kaempferol | Hyaluronan synthase 2 | HAS2 |  |
| kaempferol | Glutathione S-transferase P | GSTP1 |  |
| kaempferol | Aryl hydrocarbon receptor | AHR |  |
| kaempferol | 26S proteasome non-ATPase regulatory subunit 3 | PSMD3 |  |
| kaempferol | Solute carrier family 2, facilitated glucose transporter member 4 | SLC2A4 |  |
| kaempferol | Nuclear receptor subfamily 1 group I member 3 | NR1I3 |  |
| kaempferol | Insulin receptor | INSR |  |
| kaempferol | Type I iodothyronine deiodinase | DIO1 |  |
| kaempferol | Serine/threonine-protein phosphatase 2B catalytic subunit alpha isoform | PPP3CA |  |
| kaempferol | Glutathione S-transferase Mu 1 | GSTM1 |  |
| kaempferol | Glutathione S-transferase Mu 2 | GSTM2 |  |
| kaempferol | Aldo-keto reductase family 1 member C3 | AKR1C3 |  |
| kaempferol | Antileukoproteinase | SLPI |  |
| kaempferol | Nitric oxide synthase, inducible | NOS2 |  |
| kaempferol | Prostaglandin G/H synthase 1 | PTGS1 |  |
| kaempferol | Androgen receptor | AR |  |
| kaempferol | Peroxisome proliferator activated receptor gamma | PPARG |  |
| kaempferol | Prostaglandin G/H synthase 2 | PTGS2 |  |
| kaempferol | Nuclear receptor coactivator 2 | NCOA2 |  |
| kaempferol | Trypsin-1 | PRSS1 |  |
| kaempferol | Progesterone receptor | PGR |  |
| kaempferol | Muscarinic acetylcholine receptor M1 | CHRM1 |  |
| kaempferol | Acetylcholinesterase | ACHE |  |
| kaempferol | Sodium-dependent noradrenaline transporter | SLC6A2 |  |
| kaempferol | Muscarinic acetylcholine receptor M2 | CHRM2 |  |
| kaempferol | Alpha-1B adrenergic receptor | ADRA1B |  |
| kaempferol | Gamma-aminobutyric acid receptor subunit alpha-1 | GABRA1 |  |
| kaempferol | Coagulation factor VII | F7 |  |
| kaempferol | Transcription factor p65 | RELA |  |
| kaempferol | Inhibitor of nuclear factor kappa-B kinase subunit beta | IKBKB |  |
| kaempferol | RAC-alpha serine/threonine-protein kinase | AKT1 |  |
| kaempferol | Apoptosis regulator Bcl-2 | BCL2 |  |
| kaempferol | Apoptosis regulator BAX | BAX |  |
| kaempferol | Tumor necrosis factor | TNFSF15 |  |
| kaempferol | Transcription factor AP-1 | JUN |  |
| kaempferol | Activator of 90 kDa heat shock protein ATPase homolog 1 | AHSA1 |  |
| kaempferol | Caspase-3 | CASP3 |  |
| kaempferol | Mitogen-activated protein kinase 8 | MAPK8 |  |
| kaempferol | Interstitial collagenase | MMP1 |  |
| kaempferol | Signal transducer and activator of transcription 1-alpha/beta | STAT1 |  |
| kaempferol | Peroxisome proliferator-activated receptor gamma | PPARG |  |
| kaempferol | Heme oxygenase 1 | HMOX1 |  |
| kaempferol | Cytochrome P450 3A4 | CYP3A4 |  |
| kaempferol | Cytochrome P450 1A2 | CYP1A2 |  |
| kaempferol | Cytochrome P450 1A1 | CYP1A1 |  |
| kaempferol | Intercellular adhesion molecule 1 | ICAM1 |  |
| kaempferol | E-selectin | SELE |  |
| kaempferol | Vascular cell adhesion protein 1 | VCAM1 |  |
| kaempferol | Nuclear receptor subfamily 1 group I member 2 | NR1I2 |  |
| kaempferol | Cytochrome P450 1B1 | CYP1B1 |  |
| kaempferol | Arachidonate 5-lipoxygenase | ALOX5 |  |
| kaempferol | Hyaluronan synthase 2 | HAS2 |  |
| kaempferol | Glutathione S-transferase P | GSTP1 |  |
| kaempferol | Aryl hydrocarbon receptor | AHR |  |
| kaempferol | 26S proteasome non-ATPase regulatory subunit 3 | PSMD3 |  |
| kaempferol | Solute carrier family 2, facilitated glucose transporter member 4 | SLC2A4 |  |
| kaempferol | Nuclear receptor subfamily 1 group I member 3 | NR1I3 |  |
| kaempferol | Insulin receptor | INSR |  |
| kaempferol | Type I iodothyronine deiodinase | DIO1 |  |
| kaempferol | Serine/threonine-protein phosphatase 2B catalytic subunit alpha isoform | PPP3CA |  |
| kaempferol | Glutathione S-transferase Mu 1 | GSTM1 |  |
| kaempferol | Glutathione S-transferase Mu 2 | GSTM2 |  |
| kaempferol | Aldo-keto reductase family 1 member C3 | AKR1C3 |  |
| kaempferol | Antileukoproteinase | SLPI |  |
| Kanzonol F | Estrogen receptor | ESR1 |  |
| Kanzonol F | Androgen receptor | AR |  |
| Kanzonol F | Prostaglandin G/H synthase 2 | PTGS2 |  |
| Kanzonol F | Estrogen receptor beta | ESR2 |  |
| Kanzonol F | Nuclear receptor coactivator 2 | NCOA2 |  |
| kanzonols W | Nitric oxide synthase, inducible | NOS2 |  |
| kanzonols W | Prostaglandin G/H synthase 1 | PTGS1 |  |
| kanzonols W | Estrogen receptor | ESR1 |  |
| kanzonols W | Androgen receptor | AR |  |
| kanzonols W | Sodium channel protein type 5 subunit alpha | SCN5A |  |
| kanzonols W | Peroxisome proliferator activated receptor gamma | PPARG |  |
| kanzonols W | Prostaglandin G/H synthase 2 | PTGS2 |  |
| kanzonols W | Retinoic acid receptor RXR-alpha | RXRA |  |
| kanzonols W | Estrogen receptor beta | ESR2 |  |
| kanzonols W | Mitogen-activated protein kinase 14 | MAPK14 |  |
| kanzonols W | Glycogen synthase kinase-3 beta | GSK3B |  |
| kanzonols W | Serine/threonine-protein kinase Chk1 | CHEK1 |  |
| kanzonols W | Trypsin-1 | PRSS1 |  |
| kanzonols W | Cyclin-A2 | CCNA2 |  |
| kanzonols W | Nuclear receptor coactivator 2 | NCOA2 |  |
| kanzonols W | Nuclear receptor coactivator 1 | NCOA1 |  |
| Licoagrocarpin | Nitric oxide synthase, inducible | NOS2 |  |
| Licoagrocarpin | Prostaglandin G/H synthase 1 | PTGS1 |  |
| Licoagrocarpin | Muscarinic acetylcholine receptor M3 | CHRM3 |  |
| Licoagrocarpin | Potassium voltage-gated channel subfamily H member 2 | KCNH2 |  |
| Licoagrocarpin | Muscarinic acetylcholine receptor M1 | CHRM1 |  |
| Licoagrocarpin | Estrogen receptor | ESR1 |  |
| Licoagrocarpin | Androgen receptor | AR |  |
| Licoagrocarpin | Sodium channel protein type 5 subunit alpha | SCN5A |  |
| Licoagrocarpin | Peroxisome proliferator activated receptor gamma | PPARG |  |
| Licoagrocarpin | Muscarinic acetylcholine receptor M5 | CHRM5 |  |
| Licoagrocarpin | Prostaglandin G/H synthase 2 | PTGS2 |  |
| Licoagrocarpin | Retinoic acid receptor RXR-alpha | RXRA |  |
| Licoagrocarpin | Acetylcholinesterase | ACHE |  |
| Licoagrocarpin | Alpha-1B adrenergic receptor | ADRA1B |  |
| Licoagrocarpin | Beta-2 adrenergic receptor | ADRB2 |  |
| Licoagrocarpin | Estrogen receptor beta | ESR2 |  |
| Licoagrocarpin | Mitogen-activated protein kinase 14 | MAPK14 |  |
| Licoagrocarpin | Glycogen synthase kinase-3 beta | GSK3B |  |
| Licoagrocarpin | Retinoic acid receptor RXR-beta | RXRB |  |
| Licoagrocarpin | Trypsin-1 | PRSS1 |  |
| Licoagrocarpin | Cyclin-A2 | CCNA2 |  |
| Licoagrocarpin | Nuclear receptor coactivator 2 | NCOA2 |  |
| Licoagroisoflavone | Nitric oxide synthase, inducible | NOS2 |  |
| Licoagroisoflavone | Estrogen receptor | ESR1 |  |
| Licoagroisoflavone | Androgen receptor | AR |  |
| Licoagroisoflavone | Sodium channel protein type 5 subunit alpha | SCN5A |  |
| Licoagroisoflavone | Peroxisome proliferator activated receptor gamma | PPARG |  |
| Licoagroisoflavone | Prostaglandin G/H synthase 2 | PTGS2 |  |
| Licoagroisoflavone | Estrogen receptor beta | ESR2 |  |
| Licoagroisoflavone | Mitogen-activated protein kinase 14 | MAPK14 |  |
| Licoagroisoflavone | Glycogen synthase kinase-3 beta | GSK3B |  |
| Licoagroisoflavone | Serine/threonine-protein kinase Chk1 | CHEK1 |  |
| Licoagroisoflavone | Trypsin-1 | PRSS1 |  |
| Licoagroisoflavone | Cyclin-A2 | CCNA2 |  |
| licochalcone a | Nitric oxide synthase, inducible | NOS2 |  |
| licochalcone a | Prostaglandin G/H synthase 1 | PTGS1 |  |
| licochalcone a | Muscarinic acetylcholine receptor M1 | CHRM1 |  |
| licochalcone a | Estrogen receptor | ESR1 |  |
| licochalcone a | Androgen receptor | AR |  |
| licochalcone a | Sodium channel protein type 5 subunit alpha | SCN5A |  |
| licochalcone a | Peroxisome proliferator activated receptor gamma | PPARG |  |
| licochalcone a | Prostaglandin G/H synthase 2 | PTGS2 |  |
| licochalcone a | Alpha-1B adrenergic receptor | ADRA1B |  |
| licochalcone a | Sodium-dependent dopamine transporter | SLC6A3 |  |
| licochalcone a | Estrogen receptor beta | ESR2 |  |
| licochalcone a | Mitogen-activated protein kinase 14 | MAPK14 |  |
| licochalcone a | Glycogen synthase kinase-3 beta | GSK3B |  |
| licochalcone a | Serine/threonine-protein kinase Chk1 | CHEK1 |  |
| licochalcone a | Cyclin-A2 | CCNA2 |  |
| licochalcone a | Beta-2 adrenergic receptor | ADRB2 |  |
| licochalcone a | Nuclear receptor coactivator 2 | NCOA2 |  |
| licochalcone a | Transcription factor p65 | RELA |  |
| licochalcone a | Signal transducer and activator of transcription 3 | STAT3 |  |
| licochalcone a | G1/S-specific cyclin-D1 | CCND1 |  |
| licochalcone a | Apoptosis regulator Bcl-2 | BCL2 |  |
| licochalcone a | Eukaryotic translation initiation factor 6 | EIF6 |  |
| licochalcone a | Mitogen-activated protein kinase 1 | MAPK1 |  |
| licochalcone a | Retinoblastoma-associated protein | RB1 |  |
| licochalcone a | Fos-related antigen 2 | FOSL2 |  |
| Licochalcone B | Nitric oxide synthase, inducible | NOS2 |  |
| Licochalcone B | Prostaglandin G/H synthase 1 | PTGS1 |  |
| Licochalcone B | Estrogen receptor | ESR1 |  |
| Licochalcone B | Androgen receptor | AR |  |
| Licochalcone B | Peroxisome proliferator activated receptor gamma | PPARG |  |
| Licochalcone B | Prostaglandin G/H synthase 2 | PTGS2 |  |
| Licochalcone B | Beta-2 adrenergic receptor | ADRB2 |  |
| Licochalcone B | Estrogen receptor beta | ESR2 |  |
| Licochalcone B | Mitogen-activated protein kinase 14 | MAPK14 |  |
| Licochalcone B | Glycogen synthase kinase-3 beta | GSK3B |  |
| Licochalcone B | Serine/threonine-protein kinase Chk1 | CHEK1 |  |
| Licochalcone B | Cyclin-A2 | CCNA2 |  |
| licochalcone G | Nitric oxide synthase, inducible | NOS2 |  |
| licochalcone G | Estrogen receptor | ESR1 |  |
| licochalcone G | Androgen receptor | AR |  |
| licochalcone G | Peroxisome proliferator activated receptor gamma | PPARG |  |
| licochalcone G | Prostaglandin G/H synthase 2 | PTGS2 |  |
| licochalcone G | Vascular endothelial growth factor receptor 2 | KDR |  |
| licochalcone G | Estrogen receptor beta | ESR2 |  |
| licochalcone G | Mitogen-activated protein kinase 14 | MAPK14 |  |
| licochalcone G | Glycogen synthase kinase-3 beta | GSK3B |  |
| licochalcone G | Cyclin-A2 | CCNA2 |  |
| licochalcone G | Nuclear receptor coactivator 2 | NCOA2 |  |
| Licocoumarone | Estrogen receptor | ESR1 |  |
| Licocoumarone | Androgen receptor | AR |  |
| Licocoumarone | Estrogen receptor beta | ESR2 |  |
| Licocoumarone | Glycogen synthase kinase-3 beta | GSK3B |  |
| Licocoumarone | Cyclin-A2 | CCNA2 |  |
| licoisoflavanone | Nitric oxide synthase, inducible | NOS2 |  |
| licoisoflavanone | Prostaglandin G/H synthase 1 | PTGS1 |  |
| licoisoflavanone | Estrogen receptor | ESR1 |  |
| licoisoflavanone | Androgen receptor | AR |  |
| licoisoflavanone | Sodium channel protein type 5 subunit alpha | SCN5A |  |
| licoisoflavanone | Peroxisome proliferator activated receptor gamma | PPARG |  |
| licoisoflavanone | Prostaglandin G/H synthase 2 | PTGS2 |  |
| licoisoflavanone | Coagulation factor VII | F7 |  |
| licoisoflavanone | Acetylcholinesterase | ACHE |  |
| licoisoflavanone | Estrogen receptor beta | ESR2 |  |
| licoisoflavanone | Glycogen synthase kinase-3 beta | GSK3B |  |
| licoisoflavanone | Trypsin-1 | PRSS1 |  |
| licoisoflavanone | Cyclin-A2 | CCNA2 |  |
| licoisoflavanone | Nuclear receptor coactivator 1 | NCOA1 |  |
| Licoisoflavone | Nitric oxide synthase, inducible | NOS2 |  |
| Licoisoflavone | Estrogen receptor | ESR1 |  |
| Licoisoflavone | Androgen receptor | AR |  |
| Licoisoflavone | Peroxisome proliferator activated receptor gamma | PPARG |  |
| Licoisoflavone | Prostaglandin G/H synthase 2 | PTGS2 |  |
| Licoisoflavone | Vascular endothelial growth factor receptor 2 | KDR |  |
| Licoisoflavone | Mitogen-activated protein kinase 14 | MAPK14 |  |
| Licoisoflavone | Serine/threonine-protein kinase Chk1 | CHEK1 |  |
| Licoisoflavone | Trypsin-1 | PRSS1 |  |
| Licoisoflavone | Cyclin-A2 | CCNA2 |  |
| Licoisoflavone | Nuclear receptor coactivator 2 | NCOA2 |  |
| Licoisoflavone B | Nitric oxide synthase, inducible | NOS2 |  |
| Licoisoflavone B | Estrogen receptor | ESR1 |  |
| Licoisoflavone B | Androgen receptor | AR |  |
| Licoisoflavone B | Peroxisome proliferator activated receptor gamma | PPARG |  |
| Licoisoflavone B | Prostaglandin G/H synthase 2 | PTGS2 |  |
| Licoisoflavone B | Acetylcholinesterase | ACHE |  |
| Licoisoflavone B | Estrogen receptor beta | ESR2 |  |
| Licoisoflavone B | Glycogen synthase kinase-3 beta | GSK3B |  |
| Licoisoflavone B | Serine/threonine-protein kinase Chk1 | CHEK1 |  |
| Licoisoflavone B | Trypsin-1 | PRSS1 |  |
| Licoisoflavone B | Cyclin-A2 | CCNA2 |  |
| licopyranocoumarin | Nitric oxide synthase, inducible | NOS2 |  |
| licopyranocoumarin | Estrogen receptor | ESR1 |  |
| licopyranocoumarin | Androgen receptor | AR |  |
| licopyranocoumarin | Peroxisome proliferator activated receptor gamma | PPARG |  |
| licopyranocoumarin | Prostaglandin G/H synthase 2 | PTGS2 |  |
| licopyranocoumarin | Coagulation factor VII | F7 |  |
| licopyranocoumarin | Vascular endothelial growth factor receptor 2 | KDR |  |
| licopyranocoumarin | Acetylcholinesterase | ACHE |  |
| licopyranocoumarin | Trypsin-1 | PRSS1 |  |
| licopyranocoumarin | Cyclin-A2 | CCNA2 |  |
| Licoricone | Nitric oxide synthase, inducible | NOS2 |  |
| Licoricone | Potassium voltage-gated channel subfamily H member 2 | KCNH2 |  |
| Licoricone | Estrogen receptor | ESR1 |  |
| Licoricone | Androgen receptor | AR |  |
| Licoricone | Peroxisome proliferator activated receptor gamma | PPARG |  |
| Licoricone | Prostaglandin G/H synthase 2 | PTGS2 |  |
| Licoricone | Vascular endothelial growth factor receptor 2 | KDR |  |
| Licoricone | Serine/threonine-protein kinase Chk1 | CHEK1 |  |
| Licoricone | Trypsin-1 | PRSS1 |  |
| Licoricone | Nuclear receptor coactivator 2 | NCOA2 |  |
| liquiritin | Coagulation factor VII | F7 |  |
| liquiritin | Prostaglandin G/H synthase 2 | PTGS2 |  |
| liquiritin | Vascular endothelial growth factor receptor 2 | KDR |  |
| liquiritin | Superoxide dismutase [Cu-Zn] | SOD1 |  |
| Lupiwighteone | Nitric oxide synthase, inducible | NOS2 |  |
| Lupiwighteone | Estrogen receptor | ESR1 |  |
| Lupiwighteone | Androgen receptor | AR |  |
| Lupiwighteone | Sodium channel protein type 5 subunit alpha | SCN5A |  |
| Lupiwighteone | Peroxisome proliferator activated receptor gamma | PPARG |  |
| Lupiwighteone | Prostaglandin G/H synthase 2 | PTGS2 |  |
| Lupiwighteone | Estrogen receptor beta | ESR2 |  |
| Lupiwighteone | Mitogen-activated protein kinase 14 | MAPK14 |  |
| Lupiwighteone | Glycogen synthase kinase-3 beta | GSK3B |  |
| Lupiwighteone | Serine/threonine-protein kinase Chk1 | CHEK1 |  |
| Lupiwighteone | Trypsin-1 | PRSS1 |  |
| Lupiwighteone | Cyclin-A2 | CCNA2 |  |
| Lupiwighteone | Nuclear receptor coactivator 2 | NCOA2 |  |
| Mairin | Progesterone receptor | PGR |  |
| Mairin | Progesterone receptor | PGR |  |
| Mairin | Progesterone receptor | PGR |  |
| Mauritine D | Sodium channel protein type 5 subunit alpha | SCN5A |  |
| Mauritine D | Prostaglandin G/H synthase 2 | PTGS2 |  |
| Medicarpin | Nitric oxide synthase, inducible | NOS2 |  |
| Medicarpin | Prostaglandin G/H synthase 1 | PTGS1 |  |
| Medicarpin | Muscarinic acetylcholine receptor M3 | CHRM3 |  |
| Medicarpin | Muscarinic acetylcholine receptor M1 | CHRM1 |  |
| Medicarpin | Estrogen receptor | ESR1 |  |
| Medicarpin | Sodium channel protein type 5 subunit alpha | SCN5A |  |
| Medicarpin | Muscarinic acetylcholine receptor M5 | CHRM5 |  |
| Medicarpin | Prostaglandin G/H synthase 2 | PTGS2 |  |
| Medicarpin | Muscarinic acetylcholine receptor M4 | CHRM4 |  |
| Medicarpin | Retinoic acid receptor RXR-alpha | RXRA |  |
| Medicarpin | Alpha-1A adrenergic receptor | ADRA1A |  |
| Medicarpin | Muscarinic acetylcholine receptor M2 | CHRM2 |  |
| Medicarpin | Alpha-1B adrenergic receptor | ADRA1B |  |
| Medicarpin | Sodium-dependent dopamine transporter | SLC6A3 |  |
| Medicarpin | Beta-2 adrenergic receptor | ADRB2 |  |
| Medicarpin | Sodium-dependent serotonin transporter | SLC6A4 |  |
| Medicarpin | Mu-type opioid receptor | OPRM1 |  |
| Medicarpin | Estrogen receptor beta | ESR2 |  |
| Medicarpin | Mitogen-activated protein kinase 10 | MAPK10 |  |
| Medicarpin | Trypsin-1 | PRSS1 |  |
| Medicarpin | Cyclin-A2 | CCNA2 |  |
| Medicarpin | Delta-type opioid receptor | OPRD1 |  |
| Medicarpin | Alpha-1D adrenergic receptor | ADRA1D |  |
| naringenin | Prostaglandin G/H synthase 1 | PTGS1 |  |
| naringenin | Estrogen receptor | ESR1 |  |
| naringenin | Prostaglandin G/H synthase 2 | PTGS2 |  |
| naringenin | Transcription factor p65 | RELA |  |
| naringenin | RAC-alpha serine/threonine-protein kinase | AKT1 |  |
| naringenin | Apoptosis regulator Bcl-2 | BCL2 |  |
| naringenin | Mitogen-activated protein kinase 3 | MAPK3 |  |
| naringenin | Mitogen-activated protein kinase 1 | MAPK1 |  |
| naringenin | Caspase-3 | CASP3 |  |
| naringenin | Fatty acid synthase | FASN |  |
| naringenin | Low-density lipoprotein receptor | LDLR |  |
| naringenin | Superoxide dismutase [Cu-Zn] | SOD1 |  |
| naringenin | Catalase | CAT |  |
| naringenin | Peroxisome proliferator-activated receptor gamma | PPARG |  |
| naringenin | Microsomal triglyceride transfer protein large subunit | MTTP |  |
| naringenin | Apolipoprotein B-100 | APOB |  |
| naringenin | Phospholipase B1, membrane-associated | PLB1 |  |
| naringenin | 3-hydroxy-3-methylglutaryl-coenzyme A reductase | HMGCR |  |
| naringenin | Glutathione S-transferase P | GSTP1 |  |
| naringenin | UDP-glucuronosyltransferase 1-1 | UGT1A1 |  |
| naringenin | Peroxisome proliferator-activated receptor alpha | PPARA |  |
| naringenin | Sterol regulatory element-binding protein 1 | SREBF1 |  |
| naringenin | Glutathione reductase, mitochondrial | GSR |  |
| naringenin | Multidrug resistance-associated protein 1 | ABCC1 |  |
| naringenin | Adiponectin | ADIPOQ |  |
| naringenin | Sterol O-acyltransferase 2 | SOAT2 |  |
| naringenin | Aldo-keto reductase family 1 member C1 | AKR1C1 |  |
| naringenin | Aspartate aminotransferase, cytoplasmic | GOT1 |  |
| naringenin | 4-aminobutyrate aminotransferase, mitochondrial | ABAT |  |
| naringenin | Liver carboxylesterase 1 | CES1 |  |
| naringenin | Sterol O-acyltransferase 1 | SOAT1 |  |
| Nuciferin | Prostaglandin G/H synthase 1 | PTGS1 |  |
| Nuciferin | Muscarinic acetylcholine receptor M3 | CHRM3 |  |
| Nuciferin | Muscarinic acetylcholine receptor M1 | CHRM1 |  |
| Nuciferin | Androgen receptor | AR |  |
| Nuciferin | Sodium channel protein type 5 subunit alpha | SCN5A |  |
| Nuciferin | Muscarinic acetylcholine receptor M5 | CHRM5 |  |
| Nuciferin | Prostaglandin G/H synthase 2 | PTGS2 |  |
| Nuciferin | 5-hydroxytryptamine receptor 3A | HTR3A |  |
| Nuciferin | Muscarinic acetylcholine receptor M4 | CHRM4 |  |
| Nuciferin | Retinoic acid receptor RXR-alpha | RXRA |  |
| Nuciferin | Delta-type opioid receptor | OPRD1 |  |
| Nuciferin | Acetylcholinesterase | ACHE |  |
| Nuciferin | Sodium-dependent noradrenaline transporter | SLC6A2 |  |
| Nuciferin | Alpha-1A adrenergic receptor | ADRA1A |  |
| Nuciferin | Alpha-2B adrenergic receptor | ADRA2B |  |
| Nuciferin | Alpha-1B adrenergic receptor | ADRA1B |  |
| Nuciferin | Sodium-dependent dopamine transporter | SLC6A3 |  |
| Nuciferin | Beta-2 adrenergic receptor | ADRB2 |  |
| Nuciferin | Alpha-1D adrenergic receptor | ADRA1D |  |
| Nuciferin | Neuronal acetylcholine receptor subunit alpha-2 | CHRNA2 |  |
| Nuciferin | Sodium-dependent serotonin transporter | SLC6A4 |  |
| Nuciferin | D(2) dopamine receptor | DRD2 |  |
| Nuciferin | Mu-type opioid receptor | OPRM1 |  |
| Nuciferin | Gamma-aminobutyric acid receptor subunit alpha-1 | GABRA1 |  |
| Odoratin | Nitric oxide synthase, inducible | NOS2 |  |
| Odoratin | Prostaglandin G/H synthase 1 | PTGS1 |  |
| Odoratin | Estrogen receptor | ESR1 |  |
| Odoratin | Androgen receptor | AR |  |
| Odoratin | Sodium channel protein type 5 subunit alpha | SCN5A |  |
| Odoratin | Peroxisome proliferator activated receptor gamma | PPARG |  |
| Odoratin | Prostaglandin G/H synthase 2 | PTGS2 |  |
| Odoratin | Retinoic acid receptor RXR-alpha | RXRA |  |
| Odoratin | Estrogen receptor beta | ESR2 |  |
| Odoratin | Mitogen-activated protein kinase 14 | MAPK14 |  |
| Odoratin | Glycogen synthase kinase-3 beta | GSK3B |  |
| Odoratin | Serine/threonine-protein kinase Chk1 | CHEK1 |  |
| Odoratin | Trypsin-1 | PRSS1 |  |
| Odoratin | Cyclin-A2 | CCNA2 |  |
| Odoratin | Nuclear receptor coactivator 2 | NCOA2 |  |
| paeoniflorgenone | Gamma-aminobutyric acid receptor subunit alpha-1 | GABRA1 |  |
| paeoniflorin | Tumor necrosis factor | TNFSF15 |  |
| paeoniflorin | Interleukin-6 | IL6 |  |
| paeoniflorin | Monocyte differentiation antigen CD14 | CD14 |  |
| paeoniflorin | Lipopolysaccharide-binding protein | LBP |  |
| Phaseol | Estrogen receptor | ESR1 |  |
| Phaseol | Androgen receptor | AR |  |
| Phaseol | Peroxisome proliferator activated receptor gamma | PPARG |  |
| Phaseol | Prostaglandin G/H synthase 2 | PTGS2 |  |
| Phaseol | Vascular endothelial growth factor receptor 2 | KDR |  |
| Phaseol | Mitogen-activated protein kinase 14 | MAPK14 |  |
| Phaseol | Glycogen synthase kinase-3 beta | GSK3B |  |
| Phaseol | Serine/threonine-protein kinase Chk1 | CHEK1 |  |
| Phaseol | Cyclin-A2 | CCNA2 |  |
| Phaseolinisoflavan | Nitric oxide synthase, inducible | NOS2 |  |
| Phaseolinisoflavan | Muscarinic acetylcholine receptor M1 | CHRM1 |  |
| Phaseolinisoflavan | Estrogen receptor | ESR1 |  |
| Phaseolinisoflavan | Androgen receptor | AR |  |
| Phaseolinisoflavan | Sodium channel protein type 5 subunit alpha | SCN5A |  |
| Phaseolinisoflavan | Peroxisome proliferator activated receptor gamma | PPARG |  |
| Phaseolinisoflavan | Prostaglandin G/H synthase 2 | PTGS2 |  |
| Phaseolinisoflavan | Retinoic acid receptor RXR-alpha | RXRA |  |
| Phaseolinisoflavan | Acetylcholinesterase | ACHE |  |
| Phaseolinisoflavan | Alpha-1B adrenergic receptor | ADRA1B |  |
| Phaseolinisoflavan | Beta-2 adrenergic receptor | ADRB2 |  |
| Phaseolinisoflavan | Estrogen receptor beta | ESR2 |  |
| Phaseolinisoflavan | Mitogen-activated protein kinase 14 | MAPK14 |  |
| Phaseolinisoflavan | Glycogen synthase kinase-3 beta | GSK3B |  |
| Phaseolinisoflavan | Serine/threonine-protein kinase Chk1 | CHEK1 |  |
| Phaseolinisoflavan | Trypsin-1 | PRSS1 |  |
| Phaseolinisoflavan | Cyclin-A2 | CCNA2 |  |
| Phaseolinisoflavan | Nuclear receptor coactivator 1 | NCOA1 |  |
| poriferast-5-en-3beta-ol | Progesterone receptor | PGR |  |
| poriferast-5-en-3beta-ol | Nuclear receptor coactivator 2 | NCOA2 |  |
| quercetin | Prostaglandin G/H synthase 1 | PTGS1 |  |
| quercetin | Androgen receptor | AR |  |
| quercetin | Peroxisome proliferator activated receptor gamma | PPARG |  |
| quercetin | Prostaglandin G/H synthase 2 | PTGS2 |  |
| quercetin | Nuclear receptor coactivator 2 | NCOA2 |  |
| quercetin | Aldose reductase | AKR1B1 |  |
| quercetin | Trypsin-1 | PRSS1 |  |
| quercetin | Potassium voltage-gated channel subfamily H member 2 | KCNH2 |  |
| quercetin | Sodium channel protein type 5 subunit alpha | SCN5A |  |
| quercetin | Beta-2 adrenergic receptor | ADRB2 |  |
| quercetin | Stromelysin-1 | MMP3 |  |
| quercetin | Coagulation factor VII | F7 |  |
| quercetin | Retinoic acid receptor RXR-alpha | RXRA |  |
| quercetin | Acetylcholinesterase | ACHE |  |
| quercetin | Gamma-aminobutyric acid receptor subunit alpha-1 | GABRA1 |  |
| quercetin | Amine oxidase [flavin-containing] B | MAOB |  |
| quercetin | Transcription factor p65 | RELA |  |
| quercetin | Epidermal growth factor receptor | EGFR |  |
| quercetin | RAC-alpha serine/threonine-protein kinase | AKT1 |  |
| quercetin | Vascular endothelial growth factor A | VEGFA |  |
| quercetin | G1/S-specific cyclin-D1 | CCND1 |  |
| quercetin | Apoptosis regulator Bcl-2 | BCL2 |  |
| quercetin | Bcl-2-like protein 1 | BCL2L1 |  |
| quercetin | Proto-oncogene c-Fos | FOS |  |
| quercetin | Cyclin-dependent kinase inhibitor 1 | CDKN1A |  |
| quercetin | Eukaryotic translation initiation factor 6 | EIF6 |  |
| quercetin | Apoptosis regulator BAX | BAX |  |
| quercetin | Caspase-9 | CASP9 |  |
| quercetin | Urokinase-type plasminogen activator | PLAU |  |
| quercetin | 72 kDa type IV collagenase | MMP2 |  |
| quercetin | Matrix metalloproteinase-9 | MMP9 |  |
| quercetin | Mitogen-activated protein kinase 1 | MAPK1 |  |
| quercetin | Interleukin-10 | IL10 |  |
| quercetin | Pro-epidermal growth factor | EGF |  |
| quercetin | Retinoblastoma-associated protein | RB1 |  |
| quercetin | Tumor necrosis factor | TNFSF15 |  |
| quercetin | Transcription factor AP-1 | JUN |  |
| quercetin | Interleukin-6 | IL6 |  |
| quercetin | Activator of 90 kDa heat shock protein ATPase homolog 1 | AHSA1 |  |
| quercetin | Caspase-3 | CASP3 |  |
| quercetin | Cellular tumor antigen p53 | TP63 |  |
| quercetin | ETS domain-containing protein Elk-1 | ELK1 |  |
| quercetin | NF-kappa-B inhibitor alpha | NFKBIA |  |
| quercetin | NADPH--cytochrome P450 reductase | POR |  |
| quercetin | Ornithine decarboxylase | ODC1 |  |
| quercetin | Caspase-8 | CASP8 |  |
| quercetin | DNA topoisomerase 1 | TOP1 |  |
| quercetin | RAF proto-oncogene serine/threonine-protein kinase | RAF1 |  |
| quercetin | Superoxide dismutase [Cu-Zn] | SOD1 |  |
| quercetin | Protein kinase C alpha type | PRKCA |  |
| quercetin | Interstitial collagenase | MMP1 |  |
| quercetin | Hypoxia-inducible factor 1-alpha | HIF1A |  |
| quercetin | Signal transducer and activator of transcription 1-alpha/beta | STAT1 |  |
| quercetin | Protein CBFA2T1 | RUNX1T1 |  |
| quercetin | Receptor tyrosine-protein kinase erbB-2 | ERBB2 |  |
| quercetin | Peroxisome proliferator-activated receptor gamma | PPARG |  |
| quercetin | Acetyl-CoA carboxylase 1 | ACACA |  |
| quercetin | Heme oxygenase 1 | HMOX1 |  |
| quercetin | Cytochrome P450 3A4 | CYP3A4 |  |
| quercetin | Cytochrome P450 1A2 | CYP1A2 |  |
| quercetin | Caveolin-1 | CAV1 |  |
| quercetin | Myc proto-oncogene protein | MYC |  |
| quercetin | Tissue factor | F3 |  |
| quercetin | Gap junction alpha-1 protein | GJA1 |  |
| quercetin | Cytochrome P450 1A1 | CYP1A1 |  |
| quercetin | Intercellular adhesion molecule 1 | ICAM1 |  |
| quercetin | Interleukin-1 beta | IL1B |  |
| quercetin | C-C motif chemokine 2 | CCL2 |  |
| quercetin | E-selectin | SELE |  |
| quercetin | Vascular cell adhesion protein 1 | VCAM1 |  |
| quercetin | Prostaglandin E2 receptor EP3 subtype | PTGER3 |  |
| quercetin | Interleukin-8 | CXCL8 |  |
| quercetin | Protein kinase C beta type | PRKCB |  |
| quercetin | Baculoviral IAP repeat-containing protein 5 | BIRC5 |  |
| quercetin | Dual oxidase 2 | DUOX2 |  |
| quercetin | Nitric oxide synthase, endothelial | NOS3 |  |
| quercetin | Heat shock protein beta-1 | HSPB1 |  |
| quercetin | Estrogen sulfotransferase | SULT1E1 |  |
| quercetin | Maltase-glucoamylase, intestinal | MGAM |  |
| quercetin | Interleukin-2 | IL2 |  |
| quercetin | Nuclear receptor subfamily 1 group I member 2 | NR1I2 |  |
| quercetin | Cytochrome P450 1B1 | CYP1B1 |  |
| quercetin | G2/mitotic-specific cyclin-B1 | CCNB1 |  |
| quercetin | Tissue-type plasminogen activator | PLAT |  |
| quercetin | Thrombomodulin | THBD |  |
| quercetin | Plasminogen activator inhibitor 1 | SERPINE1 |  |
| quercetin | Collagen alpha-1(I) chain | COL1A1 |  |
| quercetin | Interferon gamma | IFNG |  |
| quercetin | Arachidonate 5-lipoxygenase | ALOX5 |  |
| quercetin | Interleukin-1 alpha | IL1A |  |
| quercetin | Myeloperoxidase | MPO |  |
| quercetin | DNA topoisomerase 2-alpha | TOP2A |  |
| quercetin | Neutrophil cytosol factor 1 | NCF1 |  |
| quercetin | ATP-binding cassette sub-family G member 2 | ABCG2 |  |
| quercetin | Hyaluronan synthase 2 | HAS2 |  |
| quercetin | Glutathione S-transferase P | GSTP1 |  |
| quercetin | Nuclear factor erythroid 2-related factor 2 | NFE2L2 |  |
| quercetin | NAD(P)H dehydrogenase [quinone] 1 | NQO1 |  |
| quercetin | Poly [ADP-ribose] polymerase 1 | PARP1 |  |
| quercetin | Aryl hydrocarbon receptor | AHR |  |
| quercetin | 26S proteasome non-ATPase regulatory subunit 3 | PSMD3 |  |
| quercetin | Solute carrier family 2, facilitated glucose transporter member 4 | SLC2A4 |  |
| quercetin | Collagen alpha-1(III) chain | COL3A1 |  |
| quercetin | C-X-C motif chemokine 11 | CXCL11 |  |
| quercetin | C-X-C motif chemokine 2 | CXCL2 |  |
| quercetin | DDB1- and CUL4-associated factor 5 | DCAF5 |  |
| quercetin | Nuclear receptor subfamily 1 group I member 3 | NR1I3 |  |
| quercetin | Serine/threonine-protein kinase Chk2 | CHEK2 |  |
| quercetin | Insulin receptor | INSR |  |
| quercetin | Claudin-4 | CLDN4 |  |
| quercetin | Peroxisome proliferator-activated receptor alpha | PPARA |  |
| quercetin | Peroxisome proliferator-activated receptor delta | PPARD |  |
| quercetin | Heat shock factor protein 1 | HSF1 |  |
| quercetin | C-reactive protein | CRP |  |
| quercetin | C-X-C motif chemokine 10 | CXCL10 |  |
| quercetin | Inhibitor of nuclear factor kappa-B kinase subunit alpha | CHUK |  |
| quercetin | Osteopontin | SPP1 |  |
| quercetin | Runt-related transcription factor 2 | RUNX2 |  |
| quercetin | Ras association domain-containing protein 1 | RASSF1 |  |
| quercetin | Transcription factor E2F1 | E2F1 |  |
| quercetin | Transcription factor E2F2 | E2F2 |  |
| quercetin | Prostatic acid phosphatase | ACPP |  |
| quercetin | Cathepsin D | CTSD |  |
| quercetin | Insulin-like growth factor-binding protein 3 | IGFBP3 |  |
| quercetin | Insulin-like growth factor II | IGF2 |  |
| quercetin | CD40 ligand | CD40LG |  |
| quercetin | Interferon regulatory factor 1 | IRF1 |  |
| quercetin | Receptor tyrosine-protein kinase erbB-3 | ERBB3 |  |
| quercetin | Serum paraoxonase/arylesterase 1 | PON1 |  |
| quercetin | Type I iodothyronine deiodinase | DIO1 |  |
| quercetin | Procollagen C-endopeptidase enhancer 1 | PCOLCE |  |
| quercetin | Puromycin-sensitive aminopeptidase | NPEPPS |  |
| quercetin | Hexokinase-2 | HK2 |  |
| quercetin | Ras GTPase-activating protein 1 | RASA1 |  |
| quercetin | Glutathione S-transferase Mu 1 | GSTM1 |  |
| quercetin | Glutathione S-transferase Mu 2 | GSTM2 |  |
| quercetin | Prostaglandin G/H synthase 1 | PTGS1 |  |
| quercetin | Androgen receptor | AR |  |
| quercetin | Peroxisome proliferator activated receptor gamma | PPARG |  |
| quercetin | Prostaglandin G/H synthase 2 | PTGS2 |  |
| quercetin | Nuclear receptor coactivator 2 | NCOA2 |  |
| quercetin | Aldose reductase | AKR1B1 |  |
| quercetin | Trypsin-1 | PRSS1 |  |
| quercetin | Potassium voltage-gated channel subfamily H member 2 | KCNH2 |  |
| quercetin | Sodium channel protein type 5 subunit alpha | SCN5A |  |
| quercetin | Beta-2 adrenergic receptor | ADRB2 |  |
| quercetin | Stromelysin-1 | MMP3 |  |
| quercetin | Coagulation factor VII | F7 |  |
| quercetin | Retinoic acid receptor RXR-alpha | RXRA |  |
| quercetin | Acetylcholinesterase | ACHE |  |
| quercetin | Gamma-aminobutyric acid receptor subunit alpha-1 | GABRA1 |  |
| quercetin | Amine oxidase [flavin-containing] B | MAOB |  |
| quercetin | Transcription factor p65 | RELA |  |
| quercetin | Epidermal growth factor receptor | EGFR |  |
| quercetin | RAC-alpha serine/threonine-protein kinase | AKT1 |  |
| quercetin | Vascular endothelial growth factor A | VEGFA |  |
| quercetin | G1/S-specific cyclin-D1 | CCND1 |  |
| quercetin | Apoptosis regulator Bcl-2 | BCL2 |  |
| quercetin | Bcl-2-like protein 1 | BCL2L1 |  |
| quercetin | Proto-oncogene c-Fos | FOS |  |
| quercetin | Cyclin-dependent kinase inhibitor 1 | CDKN1A |  |
| quercetin | Eukaryotic translation initiation factor 6 | EIF6 |  |
| quercetin | Apoptosis regulator BAX | BAX |  |
| quercetin | Caspase-9 | CASP9 |  |
| quercetin | Urokinase-type plasminogen activator | PLAU |  |
| quercetin | 72 kDa type IV collagenase | MMP2 |  |
| quercetin | Matrix metalloproteinase-9 | MMP9 |  |
| quercetin | Mitogen-activated protein kinase 1 | MAPK1 |  |
| quercetin | Interleukin-10 | IL10 |  |
| quercetin | Pro-epidermal growth factor | EGF |  |
| quercetin | Retinoblastoma-associated protein | RB1 |  |
| quercetin | Tumor necrosis factor | TNFSF15 |  |
| quercetin | Transcription factor AP-1 | JUN |  |
| quercetin | Interleukin-6 | IL6 |  |
| quercetin | Activator of 90 kDa heat shock protein ATPase homolog 1 | AHSA1 |  |
| quercetin | Caspase-3 | CASP3 |  |
| quercetin | Cellular tumor antigen p53 | TP63 |  |
| quercetin | ETS domain-containing protein Elk-1 | ELK1 |  |
| quercetin | NF-kappa-B inhibitor alpha | NFKBIA |  |
| quercetin | NADPH--cytochrome P450 reductase | POR |  |
| quercetin | Ornithine decarboxylase | ODC1 |  |
| quercetin | Caspase-8 | CASP8 |  |
| quercetin | DNA topoisomerase 1 | TOP1 |  |
| quercetin | RAF proto-oncogene serine/threonine-protein kinase | RAF1 |  |
| quercetin | Superoxide dismutase [Cu-Zn] | SOD1 |  |
| quercetin | Protein kinase C alpha type | PRKCA |  |
| quercetin | Interstitial collagenase | MMP1 |  |
| quercetin | Hypoxia-inducible factor 1-alpha | HIF1A |  |
| quercetin | Signal transducer and activator of transcription 1-alpha/beta | STAT1 |  |
| quercetin | Protein CBFA2T1 | RUNX1T1 |  |
| quercetin | Receptor tyrosine-protein kinase erbB-2 | ERBB2 |  |
| quercetin | Peroxisome proliferator-activated receptor gamma | PPARG |  |
| quercetin | Acetyl-CoA carboxylase 1 | ACACA |  |
| quercetin | Heme oxygenase 1 | HMOX1 |  |
| quercetin | Cytochrome P450 3A4 | CYP3A4 |  |
| quercetin | Cytochrome P450 1A2 | CYP1A2 |  |
| quercetin | Caveolin-1 | CAV1 |  |
| quercetin | Myc proto-oncogene protein | MYC |  |
| quercetin | Tissue factor | F3 |  |
| quercetin | Gap junction alpha-1 protein | GJA1 |  |
| quercetin | Cytochrome P450 1A1 | CYP1A1 |  |
| quercetin | Intercellular adhesion molecule 1 | ICAM1 |  |
| quercetin | Interleukin-1 beta | IL1B |  |
| quercetin | C-C motif chemokine 2 | CCL2 |  |
| quercetin | E-selectin | SELE |  |
| quercetin | Vascular cell adhesion protein 1 | VCAM1 |  |
| quercetin | Prostaglandin E2 receptor EP3 subtype | PTGER3 |  |
| quercetin | Interleukin-8 | CXCL8 |  |
| quercetin | Protein kinase C beta type | PRKCB |  |
| quercetin | Baculoviral IAP repeat-containing protein 5 | BIRC5 |  |
| quercetin | Dual oxidase 2 | DUOX2 |  |
| quercetin | Nitric oxide synthase, endothelial | NOS3 |  |
| quercetin | Heat shock protein beta-1 | HSPB1 |  |
| quercetin | Estrogen sulfotransferase | SULT1E1 |  |
| quercetin | Maltase-glucoamylase, intestinal | MGAM |  |
| quercetin | Interleukin-2 | IL2 |  |
| quercetin | Nuclear receptor subfamily 1 group I member 2 | NR1I2 |  |
| quercetin | Cytochrome P450 1B1 | CYP1B1 |  |
| quercetin | G2/mitotic-specific cyclin-B1 | CCNB1 |  |
| quercetin | Tissue-type plasminogen activator | PLAT |  |
| quercetin | Thrombomodulin | THBD |  |
| quercetin | Plasminogen activator inhibitor 1 | SERPINE1 |  |
| quercetin | Collagen alpha-1(I) chain | COL1A1 |  |
| quercetin | Interferon gamma | IFNG |  |
| quercetin | Arachidonate 5-lipoxygenase | ALOX5 |  |
| quercetin | Interleukin-1 alpha | IL1A |  |
| quercetin | Myeloperoxidase | MPO |  |
| quercetin | DNA topoisomerase 2-alpha | TOP2A |  |
| quercetin | Neutrophil cytosol factor 1 | NCF1 |  |
| quercetin | ATP-binding cassette sub-family G member 2 | ABCG2 |  |
| quercetin | Hyaluronan synthase 2 | HAS2 |  |
| quercetin | Glutathione S-transferase P | GSTP1 |  |
| quercetin | Nuclear factor erythroid 2-related factor 2 | NFE2L2 |  |
| quercetin | NAD(P)H dehydrogenase [quinone] 1 | NQO1 |  |
| quercetin | Poly [ADP-ribose] polymerase 1 | PARP1 |  |
| quercetin | Aryl hydrocarbon receptor | AHR |  |
| quercetin | 26S proteasome non-ATPase regulatory subunit 3 | PSMD3 |  |
| quercetin | Solute carrier family 2, facilitated glucose transporter member 4 | SLC2A4 |  |
| quercetin | Collagen alpha-1(III) chain | COL3A1 |  |
| quercetin | C-X-C motif chemokine 11 | CXCL11 |  |
| quercetin | C-X-C motif chemokine 2 | CXCL2 |  |
| quercetin | DDB1- and CUL4-associated factor 5 | DCAF5 |  |
| quercetin | Nuclear receptor subfamily 1 group I member 3 | NR1I3 |  |
| quercetin | Serine/threonine-protein kinase Chk2 | CHEK2 |  |
| quercetin | Insulin receptor | INSR |  |
| quercetin | Claudin-4 | CLDN4 |  |
| quercetin | Peroxisome proliferator-activated receptor alpha | PPARA |  |
| quercetin | Peroxisome proliferator-activated receptor delta | PPARD |  |
| quercetin | Heat shock factor protein 1 | HSF1 |  |
| quercetin | C-reactive protein | CRP |  |
| quercetin | C-X-C motif chemokine 10 | CXCL10 |  |
| quercetin | Inhibitor of nuclear factor kappa-B kinase subunit alpha | CHUK |  |
| quercetin | Osteopontin | SPP1 |  |
| quercetin | Runt-related transcription factor 2 | RUNX2 |  |
| quercetin | Ras association domain-containing protein 1 | RASSF1 |  |
| quercetin | Transcription factor E2F1 | E2F1 |  |
| quercetin | Transcription factor E2F2 | E2F2 |  |
| quercetin | Prostatic acid phosphatase | ACPP |  |
| quercetin | Cathepsin D | CTSD |  |
| quercetin | Insulin-like growth factor-binding protein 3 | IGFBP3 |  |
| quercetin | Insulin-like growth factor II | IGF2 |  |
| quercetin | CD40 ligand | CD40LG |  |
| quercetin | Interferon regulatory factor 1 | IRF1 |  |
| quercetin | Receptor tyrosine-protein kinase erbB-3 | ERBB3 |  |
| quercetin | Serum paraoxonase/arylesterase 1 | PON1 |  |
| quercetin | Type I iodothyronine deiodinase | DIO1 |  |
| quercetin | Procollagen C-endopeptidase enhancer 1 | PCOLCE |  |
| quercetin | Puromycin-sensitive aminopeptidase | NPEPPS |  |
| quercetin | Hexokinase-2 | HK2 |  |
| quercetin | Ras GTPase-activating protein 1 | RASA1 |  |
| quercetin | Glutathione S-transferase Mu 1 | GSTM1 |  |
| quercetin | Glutathione S-transferase Mu 2 | GSTM2 |  |
| Quercetin der. | Nitric oxide synthase, inducible | NOS2 |  |
| Quercetin der. | Prostaglandin G/H synthase 1 | PTGS1 |  |
| Quercetin der. | Estrogen receptor | ESR1 |  |
| Quercetin der. | Androgen receptor | AR |  |
| Quercetin der. | Sodium channel protein type 5 subunit alpha | SCN5A |  |
| Quercetin der. | Peroxisome proliferator activated receptor gamma | PPARG |  |
| Quercetin der. | Prostaglandin G/H synthase 2 | PTGS2 |  |
| Quercetin der. | Estrogen receptor beta | ESR2 |  |
| Quercetin der. | Mitogen-activated protein kinase 14 | MAPK14 |  |
| Quercetin der. | Glycogen synthase kinase-3 beta | GSK3B |  |
| Quercetin der. | Trypsin-1 | PRSS1 |  |
| Quercetin der. | Nuclear receptor coactivator 2 | NCOA2 |  |
| Ruvoside_qt | Mineralocorticoid receptor | NR3C2 |  |
| Ruvoside_qt | Nuclear receptor coactivator 1 | NCOA1 |  |
| Semilicoisoflavone B | Nitric oxide synthase, inducible | NOS2 |  |
| Semilicoisoflavone B | Estrogen receptor | ESR1 |  |
| Semilicoisoflavone B | Androgen receptor | AR |  |
| Semilicoisoflavone B | Sodium channel protein type 5 subunit alpha | SCN5A |  |
| Semilicoisoflavone B | Peroxisome proliferator activated receptor gamma | PPARG |  |
| Semilicoisoflavone B | Prostaglandin G/H synthase 2 | PTGS2 |  |
| Semilicoisoflavone B | Coagulation factor VII | F7 |  |
| Semilicoisoflavone B | Acetylcholinesterase | ACHE |  |
| Semilicoisoflavone B | Glycogen synthase kinase-3 beta | GSK3B |  |
| Semilicoisoflavone B | Serine/threonine-protein kinase Chk1 | CHEK1 |  |
| Semilicoisoflavone B | Trypsin-1 | PRSS1 |  |
| shinpterocarpin | Nitric oxide synthase, inducible | NOS2 |  |
| shinpterocarpin | Prostaglandin G/H synthase 1 | PTGS1 |  |
| shinpterocarpin | Muscarinic acetylcholine receptor M3 | CHRM3 |  |
| shinpterocarpin | Potassium voltage-gated channel subfamily H member 2 | KCNH2 |  |
| shinpterocarpin | Muscarinic acetylcholine receptor M1 | CHRM1 |  |
| shinpterocarpin | Estrogen receptor | ESR1 |  |
| shinpterocarpin | Androgen receptor | AR |  |
| shinpterocarpin | Sodium channel protein type 5 subunit alpha | SCN5A |  |
| shinpterocarpin | Peroxisome proliferator activated receptor gamma | PPARG |  |
| shinpterocarpin | Prostaglandin G/H synthase 2 | PTGS2 |  |
| shinpterocarpin | 5-hydroxytryptamine receptor 3A | HTR3A |  |
| shinpterocarpin | Retinoic acid receptor RXR-alpha | RXRA |  |
| shinpterocarpin | Delta-type opioid receptor | OPRD1 |  |
| shinpterocarpin | Alpha-1B adrenergic receptor | ADRA1B |  |
| shinpterocarpin | Beta-2 adrenergic receptor | ADRB2 |  |
| shinpterocarpin | Alpha-1D adrenergic receptor | ADRA1D |  |
| shinpterocarpin | Mu-type opioid receptor | OPRM1 |  |
| shinpterocarpin | Estrogen receptor beta | ESR2 |  |
| shinpterocarpin | Mitogen-activated protein kinase 14 | MAPK14 |  |
| shinpterocarpin | Glycogen synthase kinase-3 beta | GSK3B |  |
| shinpterocarpin | Retinoic acid receptor RXR-beta | RXRB |  |
| shinpterocarpin | Trypsin-1 | PRSS1 |  |
| shinpterocarpin | Cyclin-A2 | CCNA2 |  |
| shinpterocarpin | Nuclear receptor coactivator 1 | NCOA1 |  |
| Sigmoidin-B | Estrogen receptor | ESR1 |  |
| Sigmoidin-B | Prostaglandin G/H synthase 2 | PTGS2 |  |
| Sigmoidin-B | Vascular endothelial growth factor receptor 2 | KDR |  |
| sitosterol | Progesterone receptor | PGR |  |
| sitosterol | Nuclear receptor coactivator 2 | NCOA2 |  |
| sitosterol | Mineralocorticoid receptor | NR3C2 |  |
| sitosterol | Progesterone receptor | PGR |  |
| sitosterol | Nuclear receptor coactivator 2 | NCOA2 |  |
| sitosterol | Mineralocorticoid receptor | NR3C2 |  |
| sitosterol | Progesterone receptor | PGR |  |
| sitosterol | Nuclear receptor coactivator 2 | NCOA2 |  |
| sitosterol | Mineralocorticoid receptor | NR3C2 |  |
| stepharine | Prostaglandin G/H synthase 1 | PTGS1 |  |
| stepharine | Muscarinic acetylcholine receptor M3 | CHRM3 |  |
| stepharine | Muscarinic acetylcholine receptor M1 | CHRM1 |  |
| stepharine | D(1B) dopamine receptor | DRD5 |  |
| stepharine | Sodium channel protein type 5 subunit alpha | SCN5A |  |
| stepharine | Muscarinic acetylcholine receptor M5 | CHRM5 |  |
| stepharine | Prostaglandin G/H synthase 2 | PTGS2 |  |
| stepharine | 5-hydroxytryptamine receptor 3A | HTR3A |  |
| stepharine | Muscarinic acetylcholine receptor M4 | CHRM4 |  |
| stepharine | Retinoic acid receptor RXR-alpha | RXRA |  |
| stepharine | Delta-type opioid receptor | OPRD1 |  |
| stepharine | Sodium-dependent noradrenaline transporter | SLC6A2 |  |
| stepharine | Alpha-1A adrenergic receptor | ADRA1A |  |
| stepharine | Muscarinic acetylcholine receptor M2 | CHRM2 |  |
| stepharine | Alpha-2B adrenergic receptor | ADRA2B |  |
| stepharine | Alpha-1B adrenergic receptor | ADRA1B |  |
| stepharine | Sodium-dependent dopamine transporter | SLC6A3 |  |
| stepharine | Beta-2 adrenergic receptor | ADRB2 |  |
| stepharine | Neuronal acetylcholine receptor subunit alpha-2 | CHRNA2 |  |
| stepharine | Sodium-dependent serotonin transporter | SLC6A4 |  |
| stepharine | D(2) dopamine receptor | DRD2 |  |
| stepharine | Mu-type opioid receptor | OPRM1 |  |
| stepharine | Gamma-aminobutyric acid receptor subunit alpha-1 | GABRA1 |  |
| Stepholidine | Prostaglandin G/H synthase 1 | PTGS1 |  |
| Stepholidine | Muscarinic acetylcholine receptor M3 | CHRM3 |  |
| Stepholidine | Potassium voltage-gated channel subfamily H member 2 | KCNH2 |  |
| Stepholidine | Muscarinic acetylcholine receptor M1 | CHRM1 |  |
| Stepholidine | D(1B) dopamine receptor | DRD5 |  |
| Stepholidine | Sodium channel protein type 5 subunit alpha | SCN5A |  |
| Stepholidine | Muscarinic acetylcholine receptor M5 | CHRM5 |  |
| Stepholidine | Prostaglandin G/H synthase 2 | PTGS2 |  |
| Stepholidine | Alpha-2C adrenergic receptor | ADRA2C |  |
| Stepholidine | D(4) dopamine receptor | DRD4 |  |
| Stepholidine | Muscarinic acetylcholine receptor M4 | CHRM4 |  |
| Stepholidine | Retinoic acid receptor RXR-alpha | RXRA |  |
| Stepholidine | Delta-type opioid receptor | OPRD1 |  |
| Stepholidine | Muscarinic acetylcholine receptor M2 | CHRM2 |  |
| Stepholidine | Alpha-2B adrenergic receptor | ADRA2B |  |
| Stepholidine | Alpha-1B adrenergic receptor | ADRA1B |  |
| Stepholidine | Sodium-dependent dopamine transporter | SLC6A3 |  |
| Stepholidine | Beta-2 adrenergic receptor | ADRB2 |  |
| Stepholidine | Alpha-1D adrenergic receptor | ADRA1D |  |
| Stepholidine | Sodium-dependent serotonin transporter | SLC6A4 |  |
| Stepholidine | Mu-type opioid receptor | OPRM1 |  |
| Stigmasterol | Progesterone receptor | PGR |  |
| Stigmasterol | Mineralocorticoid receptor | NR3C2 |  |
| Stigmasterol | Nuclear receptor coactivator 2 | NCOA2 |  |
| Stigmasterol | Alcohol dehydrogenase 1C | ADH1C |  |
| Stigmasterol | Retinoic acid receptor RXR-alpha | RXRA |  |
| Stigmasterol | Nuclear receptor coactivator 1 | NCOA1 |  |
| Stigmasterol | Prostaglandin G/H synthase 1 | PTGS1 |  |
| Stigmasterol | Prostaglandin G/H synthase 2 | PTGS2 |  |
| Stigmasterol | Alpha-2A adrenergic receptor | ADRA2A |  |
| Stigmasterol | Sodium-dependent noradrenaline transporter | SLC6A2 |  |
| Stigmasterol | Sodium-dependent dopamine transporter | SLC6A3 |  |
| Stigmasterol | Beta-2 adrenergic receptor | ADRB2 |  |
| Stigmasterol | Aldose reductase | AKR1B1 |  |
| Stigmasterol | Urokinase-type plasminogen activator | PLAU |  |
| Stigmasterol | Leukotriene A-4 hydrolase | LTA4H |  |
| Stigmasterol | Amine oxidase [flavin-containing] B | MAOB |  |
| Stigmasterol | Amine oxidase [flavin-containing] A | MAOA |  |
| Stigmasterol | Chymotrypsinogen B | CTRB1 |  |
| Stigmasterol | Muscarinic acetylcholine receptor M3 | CHRM3 |  |
| Stigmasterol | Muscarinic acetylcholine receptor M1 | CHRM1 |  |
| Stigmasterol | Beta-1 adrenergic receptor | ADRB1 |  |
| Stigmasterol | Sodium channel protein type 5 subunit alpha | SCN5A |  |
| Stigmasterol | Alpha-1A adrenergic receptor | ADRA1A |  |
| Stigmasterol | Muscarinic acetylcholine receptor M2 | CHRM2 |  |
| Stigmasterol | Alpha-1B adrenergic receptor | ADRA1B |  |
| Stigmasterol | Gamma-aminobutyric acid receptor subunit alpha-1 | GABRA1 |  |
| Stigmasterol | Progesterone receptor | PGR |  |
| Stigmasterol | Mineralocorticoid receptor | NR3C2 |  |
| Stigmasterol | Nuclear receptor coactivator 2 | NCOA2 |  |
| Stigmasterol | Alcohol dehydrogenase 1C | ADH1C |  |
| Stigmasterol | Retinoic acid receptor RXR-alpha | RXRA |  |
| Stigmasterol | Nuclear receptor coactivator 1 | NCOA1 |  |
| Stigmasterol | Prostaglandin G/H synthase 1 | PTGS1 |  |
| Stigmasterol | Prostaglandin G/H synthase 2 | PTGS2 |  |
| Stigmasterol | Alpha-2A adrenergic receptor | ADRA2A |  |
| Stigmasterol | Sodium-dependent noradrenaline transporter | SLC6A2 |  |
| Stigmasterol | Sodium-dependent dopamine transporter | SLC6A3 |  |
| Stigmasterol | Beta-2 adrenergic receptor | ADRB2 |  |
| Stigmasterol | Aldose reductase | AKR1B1 |  |
| Stigmasterol | Urokinase-type plasminogen activator | PLAU |  |
| Stigmasterol | Leukotriene A-4 hydrolase | LTA4H |  |
| Stigmasterol | Amine oxidase [flavin-containing] B | MAOB |  |
| Stigmasterol | Amine oxidase [flavin-containing] A | MAOA |  |
| Stigmasterol | Chymotrypsinogen B | CTRB1 |  |
| Stigmasterol | Muscarinic acetylcholine receptor M3 | CHRM3 |  |
| Stigmasterol | Muscarinic acetylcholine receptor M1 | CHRM1 |  |
| Stigmasterol | Beta-1 adrenergic receptor | ADRB1 |  |
| Stigmasterol | Sodium channel protein type 5 subunit alpha | SCN5A |  |
| Stigmasterol | Alpha-1A adrenergic receptor | ADRA1A |  |
| Stigmasterol | Muscarinic acetylcholine receptor M2 | CHRM2 |  |
| Stigmasterol | Alpha-1B adrenergic receptor | ADRA1B |  |
| Stigmasterol | Gamma-aminobutyric acid receptor subunit alpha-1 | GABRA1 |  |
| taxifolin | Prostaglandin G/H synthase 1 | PTGS1 |  |
| taxifolin | Prostaglandin G/H synthase 2 | PTGS2 |  |
| taxifolin | Retinoic acid receptor RXR-alpha | RXRA |  |
| taxifolin | Aldose reductase | AKR1B1 |  |
| taxifolin | Transcription factor p65 | RELA |  |
| taxifolin | Intercellular adhesion molecule 1 | ICAM1 |  |
| taxifolin | Diacylglycerol O-acyltransferase 2 | DGAT2 |  |
| taxifolin | Microsomal triglyceride transfer protein large subunit | MTTP |  |
| taxifolin | Apolipoprotein B-100 | APOB |  |
| Vestitol | Nitric oxide synthase, inducible | NOS2 |  |
| Vestitol | Prostaglandin G/H synthase 1 | PTGS1 |  |
| Vestitol | Muscarinic acetylcholine receptor M1 | CHRM1 |  |
| Vestitol | Estrogen receptor | ESR1 |  |
| Vestitol | Androgen receptor | AR |  |
| Vestitol | Sodium channel protein type 5 subunit alpha | SCN5A |  |
| Vestitol | Peroxisome proliferator activated receptor gamma | PPARG |  |
| Vestitol | Prostaglandin G/H synthase 2 | PTGS2 |  |
| Vestitol | Muscarinic acetylcholine receptor M4 | CHRM4 |  |
| Vestitol | Retinoic acid receptor RXR-alpha | RXRA |  |
| Vestitol | Alpha-1A adrenergic receptor | ADRA1A |  |
| Vestitol | Alpha-1B adrenergic receptor | ADRA1B |  |
| Vestitol | Sodium-dependent dopamine transporter | SLC6A3 |  |
| Vestitol | Beta-2 adrenergic receptor | ADRB2 |  |
| Vestitol | Sodium-dependent serotonin transporter | SLC6A4 |  |
| Vestitol | Estrogen receptor beta | ESR2 |  |
| Vestitol | Mitogen-activated protein kinase 14 | MAPK14 |  |
| Vestitol | Glycogen synthase kinase-3 beta | GSK3B |  |
| Vestitol | Serine/threonine-protein kinase Chk1 | CHEK1 |  |
| Vestitol | Trypsin-1 | PRSS1 |  |
| Vestitol | Cyclin-A2 | CCNA2 |  |
| Vestitol | cAMP-dependent protein kinase inhibitor alpha | PKIA |  |
| Xambioona | Nitric oxide synthase, inducible | NOS2 |  |
| Xambioona | Estrogen receptor | ESR1 |  |
| Xambioona | Prostaglandin G/H synthase 2 | PTGS2 |  |
| Xambioona | Estrogen receptor beta | ESR2 |  |
| Xambioona | Nuclear receptor coactivator 2 | NCOA2 |  |
| zizyphus saponin I_qt | Mineralocorticoid receptor | NR3C2 |  |

Supplementary Table 3: Potential targets of hypertension

| Target | Database | Target | Database |
| --- | --- | --- | --- |
| ACE | GeneCards,CTD | ATP1B1 | GeneCards,CTD |
| ACE2 | GeneCards,OMIM,CTD | ATP2B1 | GeneCards,CTD |
| ACVRL1 | GeneCards,OMIM | BDKRB2 | GeneCards,CTD |
| ADD1 | GeneCards,OMIM,CTD | CAT | GeneCards,CTD |
| ADD2 | GeneCards,OMIM | CAV1 | GeneCards,OMIM |
| ADIPOQ | GeneCards,CTD | CCL2 | GeneCards,CTD |
| ADM | GeneCards,OMIM | CCN2 | GeneCards,CTD |
| ADRB1 | GeneCards,CTD | CD36 | OMIM,CTD |
| AGT | GeneCards,CTD | CORIN | GeneCards,OMIM |
| AGTR1 | GeneCards,OMIM,CTD | CPS1 | GeneCards,OMIM |
| ALB | GeneCards,CTD | CRP | GeneCards,CTD |
| APOA1 | GeneCards,CTD | CYBA | GeneCards,CTD |
| ATP1A1 | OMIM,CTD | CYP17A1 | GeneCards,OMIM,CTD |
| F12 | GeneCards,CTD | CYP3A5 | GeneCards,OMIM,CTD |
| FBN1 | GeneCards,OMIM | CYP4A11 | GeneCards,OMIM |
| FN1 | GeneCards,CTD | DGUOK | GeneCards,OMIM |
| FOS | GeneCards,CTD | EDN1 | GeneCards,CTD |
| GNAS | GeneCards,CTD | EDNRA | GeneCards,CTD |
| GNB3 | GeneCards,OMIM,CTD | ELN | GeneCards,OMIM,CTD |
| GRK2 | OMIM,CTD | EPO | GeneCards,CTD |
| GRK4 | GeneCards,OMIM | ERAP1 | OMIM,CTD |
|  |  |  |  |
| HSD11B1 | GeneCards,CTD | NEDD4L | GeneCards,CTD |
| HSD11B2 | GeneCards,OMIM,CTD | NOS2 | GeneCards,CTD |
| HTR2B | OMIM,CTD | NOS3 | GeneCards,CTD |
| ICAM1 | GeneCards,CTD | NPPA | GeneCards,OMIM,CTD |
| IGF1 | GeneCards,CTD | NPPB | GeneCards,CTD |
| IL1B | GeneCards,CTD | NPR1 | OMIM,CTD |
| IL6 | GeneCards,CTD | NR3C1 | GeneCards,CTD |
| INS | GeneCards,CTD | NR3C2 | GeneCards,OMIM,CTD |
| KCNJ5 | GeneCards,OMIM | OLR1 | GeneCards,CTD |
| KCNK3 | GeneCards,OMIM | PDE3A | GeneCards,OMIM,CTD |
| KCNMB1 | GeneCards,OMIM,CTD | PGF | GeneCards,OMIM |
| KLHL3 | GeneCards,OMIM | PLAT | GeneCards,CTD |
| KNG1 | GeneCards,CTD | POMC | GeneCards,CTD |
| LEP | GeneCards,OMIM,CTD | PPARG | GeneCards,OMIM,CTD |
| LEPR | GeneCards,CTD | PPBP | GeneCards,CTD |
| LPL | GeneCards,CTD | PTGIS | GeneCards,CTD |
| MMP2 | GeneCards,CTD | REN | GeneCards,OMIM,CTD |
| MMP9 | GeneCards,CTD | RGS2 | GeneCards,OMIM,CTD |
| NCF1 | OMIM,CTD | RGS5 | GeneCards,CTD |
| SERPINE1 | GeneCards,CTD | SARS2 | GeneCards,OMIM |
| SLC12A3 | GeneCards,CTD | SCNN1A | GeneCards,OMIM,CTD |
| SLC6A4 | GeneCards,CTD | SCNN1B | GeneCards,OMIM,CTD |
| SMAD9 | GeneCards,OMIM | SCNN1G | GeneCards,OMIM,CTD |
| SOD1 | GeneCards,CTD | SELP | GeneCards,CTD |
| STK39 | GeneCards,CTD | ENG | GeneCards |
| TBX4 | GeneCards,OMIM | BMPR2 | GeneCards |
| TGFB1 | GeneCards,OMIM,CTD | PKD1 | GeneCards |
| TIMP1 | GeneCards,CTD | THBD | GeneCards |
| TNF | GeneCards,CTD | ADRB2 | GeneCards |
| TP53 | GeneCards,CTD | APOB | GeneCards |
| UMOD | GeneCards,CTD | ADRB3 | GeneCards |
| UTS2 | GeneCards,CTD | B2M | GeneCards |
| VCAM1 | GeneCards,CTD | GBA | GeneCards |
| VEGFA | GeneCards,OMIM | COMT | GeneCards |
| VIP | GeneCards,OMIM | PKD2 | GeneCards |
| VWF | GeneCards,CTD | INSR | GeneCards |
| WNK1 | GeneCards,OMIM,CTD | MGP | GeneCards |
| WNK4 | GeneCards,OMIM,CTD | RETN | GeneCards |
| YY1AP1 | OMIM,CTD | MEN1 | GeneCards |
| HYT8 | GeneCards | IRS1 | GeneCards |
| HYT1 | GeneCards | FOXF1 | GeneCards |
| DARS2 | GeneCards | COL4A5 | GeneCards |
| GCGR | GeneCards | MTHFR | GeneCards |
| HYT2 | GeneCards | SMAD4 | GeneCards |
| HYT5 | GeneCards | F5 | GeneCards |
| HYT6 | GeneCards | HYT4 | GeneCards |
| HYT7 | GeneCards | HYT3 | GeneCards |
| RET | GeneCards | ENPP1 | GeneCards |
| ABCA3 | GeneCards | ENPEP | GeneCards |
| EIF2AK4 | GeneCards | NOTCH1 | GeneCards |
| MIR155 | GeneCards | FLT1 | GeneCards |
| INF2 | GeneCards | CACNA1H | GeneCards |
| JAK2 | GeneCards | BBS2 | GeneCards |
| CUL3 | GeneCards | LMNA | GeneCards |
| SELE | GeneCards | F3 | GeneCards |
| SDHB | GeneCards | EDN2 | GeneCards |
| PMS2 | GeneCards | RPL5 | GeneCards |
| VHL | GeneCards | NPY | GeneCards |
| PKHD1 | GeneCards | SERPINC1 | GeneCards |
| F2 | GeneCards | CYP21A2 | GeneCards |
| H19 | GeneCards | NFU1 | GeneCards |
| SGK1 | GeneCards | NPR3 | GeneCards |
| SDHD | GeneCards | HLA-DRB1 | GeneCards |
| EDNRB | GeneCards | PDE5A | GeneCards |
| CHGA | GeneCards | BBS1 | GeneCards |
| MIR204 | GeneCards | CST3 | GeneCards |
| FGA | GeneCards | COMMD5 | OMIM |
| TMEM67 | GeneCards | BMPR2 | OMIM |
| THPO | GeneCards | GUCY1A3 | OMIM |
| SDHC | GeneCards | NOTCH3 | OMIM |
| NF1 | GeneCards | CALCRL | OMIM |
| BMP6 | GeneCards | SMAD1 | OMIM |
| APLNR | GeneCards | RAMP2 | OMIM |
| MT-ND1 | GeneCards | AKT1 | OMIM，Drugbank |
| CLCN2 | GeneCards | ACSM3 | OMIM |
| ACTA2 | GeneCards | ALOX5 | OMIM |
| APLN | GeneCards | TRPC6 | OMIM |
| PRL | GeneCards | SLC9A1 | OMIM |
| NISCH | GeneCards | LRP6 | OMIM |
| PON1 | GeneCards | KYNU | OMIM |
| STOX1 | GeneCards | NOS2A | OMIM |
| XDH | GeneCards | TNFRSF1B | OMIM |
| LPA | GeneCards | ABCC6 | OMIM |
| PRKAR1A | GeneCards | APOL1 | OMIM |
| CACNA1D | GeneCards | SLC39A12 | OMIM |
| SLC12A1 | GeneCards | MMACHC | OMIM |
| TGFB3 | GeneCards | MEX3C | OMIM |
| BBS4 | GeneCards | ABCC1 | CTD |
| SPP1 | GeneCards | ADCY5 | CTD |
| ARMC5 | GeneCards | ADRA2A | CTD |
| DBH | GeneCards | AGTR1B | CTD |
| PEE1 | GeneCards | AHR | CTD |
| TGFBR1 | GeneCards | ALAD | CTD |
| JUN | CTD | ALOX15 | CTD |
| KCNJ1 | CTD | ANXA1 | CTD |
| KLK1 | CTD | AOC1 | CTD |
| LHB | CTD | AOC3 | CTD |
| LTF | CTD | AQP1 | CTD |
| MC2R | CTD | AR | CTD |
| MCPT1 | CTD | ATOX1 | CTD |
| MDH1 | CTD | ATP1A2 | CTD |
| MOXD2 | CTD | ATP2B3 | CTD |
| MTOR | CTD | AVP | CTD |
| MYD88 | CTD | BCL2 | CTD |
| MYH9 | CTD | CACNA1C | CTD |
| NOX4 | CTD | CACNA2D1 | CTD |
| NPTN | CTD | CALY | CTD |
| ORAI1 | CTD | CBS | CTD |
| OXSR1 | CTD | CELA2A | CTD |
| OXT | CTD | CETP | CTD |
| PLG | CTD | CLCNKA | CTD |
| PPARA | CTD | CMA1 | CTD |
| PRKCD | CTD | COL3A1 | CTD |
| PROC | CTD | CRHR2 | CTD |
| PTEN | CTD | CXCL2 | CTD |
| PTGS2 | CTD | CXCR2 | CTD |
| PTH | CTD | CYBB | CTD |
| PTPN1 | CTD | CYP1A1 | CTD |
| RALBP1 | CTD | CYP4A10 | CTD |
| REG3G | CTD | DRD2 | CTD |
| RELA | CTD | DUSP5 | CTD |
| REN2 | CTD | EDN3 | CTD |
| RPS6KB1 | CTD | F11 | CTD |
| SLC12A2 | CTD | FXYD2 | CTD |
| SLC6A18 | CTD | GAL | CTD |
| SLC6A2 | CTD | GCG | CTD |
| SLC9A3R2 | CTD | GCH1 | CTD |
| SOD2 | CTD | GCLC | CTD |
| SOD3 | CTD | GJA1 | CTD |
| STIM1 | CTD | GJA4 | CTD |
| STK11 | CTD | GJA5 | CTD |
| TACR1 | CTD | GJC1 | CTD |
| TGM2 | CTD | GLP1R | CTD |
| TH | CTD | GNRH1 | CTD |
| TLR4 | CTD | GP1BA | CTD |
| TPM1 | CTD | GPX1 | CTD |
| TRH | CTD | GSK3B | CTD |
| TRPC3 | CTD | GSTA5 | CTD |
| TRPC5 | CTD | GSTP1 | CTD |
| UCN | CTD | GSTT1 | CTD |
| UCP2 | CTD | GSTT2 | CTD |
| VAV3 | CTD | GUCY1A1 | CTD |
| VDR | CTD | HIF1A | CTD |
| IKBKB | CTD | HMOX1 | CTD |
| INPPL1 | CTD | HP | CTD |
| ITM2B | CTD | HRH3 | CTD |
| IER3 | CTD |  |  |
